# Past, present, and future of precision determinations of the QCD coupling from lattice QCD

**DOI:** 10.1140/epja/s10050-021-00381-3

**Published:** 2021-02-22

**Authors:** Mattia Dalla Brida

**Affiliations:** 1 Dipartimento di Fisica, Università di Milano-Bicocca, Piazza della Scienza 3, 20126 Milan, Italy; 2 INFN, Sezione di Milano-Bicocca, Piazza della Scienza 3, 20126 Milan, Italy; 3 Department of Physics, University of Cyprus, P.O. Box 20537, 1678 Nicosia, Cyprus

## Abstract

Non-perturbative scale-dependent renormalization problems are ubiquitous in lattice QCD as they enter many relevant phenomenological applications. They require solving non-perturbatively the renormalization group equations for the QCD parameters and matrix elements of interest in order to relate their non-perturbative determinations at low energy to their high-energy counterparts needed for phenomenology. Bridging the large energy separation between the hadronic and perturbative regimes of QCD, however, is a notoriously difficult task. In this contribution we focus on the case of the QCD coupling. We critically address the common challenges that state-of-the-art lattice determinations have to face in order to be significantly improved. In addition, we review a novel strategy that has been recently put forward in order to solve this non-perturbative renormalization problem and discuss its implications for future precision determinations. The new ideas exploit the decoupling of heavy quarks to match ${N_{\mathrm{f}}}$-flavor QCD and the pure Yang–Mills theory. Through this matching the computation of the non-perturbative running of the coupling in QCD can be shifted to the computationally much easier to solve pure-gauge theory. We shall present results for the determination of the $\varLambda$-parameter of ${N_{\mathrm{f}}}=3$-flavor QCD where this strategy has been applied and proven successful. The results demonstrate that these techniques have the potential to unlock unprecedented precision determinations of the QCD coupling from the lattice. The ideas are moreover quite general and can be considered to solve other non-perturbative renormalization problems.

## Introduction

Renormalization is a fundamental step in order to extract (meaningful) phenomenologically relevant results from lattice QCD calculations. For the lattice theorist it is natural to renormalize the bare parameters of the lattice QCD Lagrangian and the composite operators of interest in terms of some hadronic renormalization schemes (cf. Refs. [1, 2]). In order to make the determinations accessible to phenomenologists, however, it is often necessary to translate the results obtained in the chosen hadronic schemes to results in the (perturbative) schemes and at the scales commonly considered in phenomenology. In practice, this requires the determination of the non-perturbative renormalization group (RG) running of the renormalized QCD parameters and operators in some convenient intermediate scheme, from the hadronic scales where they were originally defined, up to some high-energy scale, where perturbation theory eventually applies and a matching to phenomenological schemes can be performed.

Over the last decade or so, lattice QCD has entered a precision era for an increasingly large set of quantities (cf. Ref. [3]). Renormalization is a relevant part of many of these computations where it can significantly impact the quality of the final results. Hence, as we are forced to become more aware of all possible sources of uncertainties in the determination of the bare lattice quantities, the same care must be reserved to their renormalization. In particular, as any other lattice calculation, besides the statistical errors the determinations of renormalized parameters and operators have their systematics to deal with, i.e. discretization effects, finite-volume effects, quark-mass effects, and, when a matching to phenomenological schemes is necessary, also perturbative uncertainties. It is therefore important that the development in strategies to compute (bare) lattice quantities is accompanied with new ideas to improve their renormalization, so to guarantee a precise and robust end result.

An extreme example of this situation, if we can call it this way, is the determination of the QCD parameters. In this case we can say that the problem is entirely a renormalization problem, which, however, has very important phenomenological applications. On the lattice, the QCD coupling and quark masses are renormalized in terms of hadronic masses and decay constants, while in phenomenology the QCD parameters are needed at energies of the order of a hundred $\mathrm{GeV}$ and above. One would thus think that lattice QCD is not the right tool for providing this information given the very high energies involved. It appears more natural indeed to obtain these parameters directly from high-energy quantities, rather than from the hadronic spectrum. As we shall recall later in this contribution, this is actually not the case, as lattice techniques offer an ideal framework for these computations.

For the last 10–15 years, lattice QCD has consistently delivered some of the most precise determinations for the QCD parameters, as in particular for the QCD coupling $\alpha _{\mathrm{s}}$ (see e.g. Refs. [3–5]).^1^ The current world average for the QCD coupling evaluated for reference at the *Z*-boson pole mass $M_Z$ is $\alpha _{\mathrm{s}}(M_Z)=0.1179(10)$ [5], and has a precision of about 0.8%. The lattice determinations alone give $\alpha _{\mathrm{s}}(M_Z)=0.1182(8)$ [3], and are the most precise subcategory of those considered by the PDG. Besides the high precision of the individual state-of-the-art determinations, it is important to emphasize also their overall consistency. This is a rather non-trivial result considering the fact that even though all lattice determinations share some common systematics, these are probed quite differently by considering very different strategies [3]. It is fair to say that such a variety of approaches within a PDG subcategory is in fact unique [5].

Despite the tremendous efforts on and off the lattice, however, the current uncertainty on $\alpha _{\mathrm{s}}$ is still large. It is one of the largest sources of uncertainty in several key processes, particularly so within the Higgs sector, and it is expected to be a limiting factor in many high-precision studies at future colliders (see e.g. Refs. [4, 6]). An uncertainty on $\alpha _{\mathrm{s}}(M_Z)$ comfortably below the percent level is desired for precision applications. For these reasons, there are plans for future phenomenological determinations of $\alpha _{\mathrm{s}}(M_Z)$ aiming at reaching the extremely competitive accuracy of 0.2% using high-luminosity high-energy data (see e.g. Refs. [7–10]). The lattice community needs to meet the challenge.

Reducing the current uncertainties on lattice determinations of $\alpha _{\mathrm{s}}$ by such an important factor is not easy. Similarly to several phenomenological determinations most lattice determinations of $\alpha _{\mathrm{s}}$ are currently limited by systematic uncertainties related to the use of perturbation theory at relatively low scales [3]. The issue is due to the fact that reaching high energy on the lattice requires small lattice spacings to be simulated and this is in general difficult without a dedicated strategy.

A way around this has been known since a long time and it is based on the concepts of finite-volume renormalization schemes and finite-size scaling (or step-scaling) techniques [11, 12]. The methods have been recently applied for obtaining one of the most precise determinations of $\alpha _{\mathrm{s}}$ [13]. The key feature of the approach is that it allows for reaching high energy with all systematics under control. This puts the lattice determinations in the privileged position of being able to reach in a clean and controlled way high energies fully non-perturbatively. The systematics due to the application of perturbation theory, in particular, can be entirely avoided at the expenses of the statistical errors accumulated in running from low up to high-enough energy. The net advantage of this situation is that differently from systematic uncertainties, statistical errors can be straightforwardly reduced. Nonetheless, a reduction of the current uncertainties on $\alpha _{\mathrm{s}}$ by an important factor is yet a computationally expensive task, even employing a step-scaling strategy (cf. Ref. [13]).

In this contribution we want to review the recent proposal made in Ref. [14] which may allow for such error reduction in a substantially cheaper way. The key feature of this proposal is that one can replace the computation of the RG running of the coupling in ${N_{\mathrm{f}}}$-flavor QCD with that in the pure-gauge theory. It is clear that, regardless of the chosen strategy, this allows for a substantial simplification of the problem.

In short, the idea is built on three main steps and exploits the decoupling of heavy quarks in a couple of ways. In the first step, heavy-quark decoupling is used to connect a low-energy scale $\mu _{\mathrm{dec}}$ in ${N_{\mathrm{f}}}$-flavor QCD with the corresponding scale in the pure-gauge theory. This is achieved through the computation of a massive renormalized coupling in an (unphysical) theory with ${N_{\mathrm{f}}}$ heavy quarks of mass $M\gg \mu _{\mathrm{dec}}$. In a second step, by computing the non-perturbative RG running in the pure Yang-Mills theory of a convenient coupling one obtains the pure-gauge $\varLambda$-parameter in units of $\mu _{\mathrm{dec}}$, i.e. $\varLambda ^{({N_{\mathrm{f}}}=0)}_{\overline{\mathrm{MS}}}/\mu _{\mathrm{dec}}$. Finally, perturbative decoupling relations are invoked at a scale $\mu \approx M$ to estimate the ratio of $\varLambda$-parameters in the ${N_{\mathrm{f}}}$-flavor and pure Yang-Mills theory, that is, $\varLambda ^{({N_{\mathrm{f}}}=0)}_{\overline{\mathrm{MS}}}/ \varLambda ^{({N_{\mathrm{f}}})}_{\overline{\mathrm{MS}}}$. Putting these steps together, one obtains $\varLambda ^{({N_{\mathrm{f}}})}_{\overline{\mathrm{MS}}}/\mu _{\mathrm{dec}}$, and given the physical value of $\mu _{\mathrm{dec}}$ finds $\varLambda ^{({N_{\mathrm{f}}})}_{\overline{\mathrm{MS}}}$. Considering ${N_{\mathrm{f}}}=3$ or 4, once $\varLambda ^{({N_{\mathrm{f}}})}_{\overline{\mathrm{MS}}}$ is determined one proceeds as usual and applies perturbative decoupling relations at the charm and/or bottom quark-mass scale to estimate $\varLambda ^{({N_{\mathrm{f}}}=5)}_{\overline{\mathrm{MS}}}$ and from it $\alpha _{\mathrm{s}}(M_Z)$.

The strategy has already been proven successful in the determination of $\varLambda ^{({N_{\mathrm{f}}}=3)}_{\overline{\mathrm{MS}}}$ [14]. The ideas presented in this reference are however general and may be applied to solve other non-perturbative scale-dependent renormalization problems that face analogous challenges.

The outline of this contribution is the following.

We begin in Sect. 2 by recalling the main challenges in solving scale-dependent renormalization problems on the lattice. The emphasis will be on the determination of the QCD coupling. Besides introducing important concepts for later sections, the presentation gives us the opportunity to discuss some recent interesting determinations. These clearly illustrate the difficulties that state-of-the-art computations of the coupling have to face in order to be significantly improved.

In Sect. 3, we introduce the theory of heavy-quark decoupling and present the results of several recent studies that systematically assessed the size of non-perturbative effects induced by heavy quarks. More precisely, the accuracy of using perturbative decoupling relations to match the $\varLambda$-parameters of different ${N_{\mathrm{f}}}$-flavor theories is investigated, as well as the corrections due to the heavy quarks in low-energy quantities. These studies not only set the foundation for the renormalization strategy based on decoupling, but also establish the precision at which $\alpha _{\mathrm{s}}$ can be obtained from results in ${N_{\mathrm{f}}}=3$ QCD.

In Sect. 4, the application of heavy-quark decoupling to the determination of the ${N_{\mathrm{f}}}$-flavor QCD coupling is described in detail and the results of Ref. [14] for $\varLambda ^{({N_{\mathrm{f}}}=3)}_{\overline{\mathrm{MS}}}$ are presented. We conclude in Sect. 5 with some comments on the future prospects for $\alpha _{\mathrm{s}}$ determinations in view of this new strategy.

We care to note that it is not the aim of the present contribution to discuss in detail the many different lattice approaches that are currently considered to determine the QCD coupling. In particular, we do not provide a complete account of all recent determinations. For such a discussion, we refer the interested reader to the comprehensive work of FLAG [3] and to other interesting recent reviews (see e.g. Refs. [15, 16]).

## Precision determinations: the case of $\alpha _{\mathrm{s}}$

Before presenting the renormalization ideas based on decoupling, we believe it is important to put these into context. The aim of these strategies, in fact, is not simply that of providing alternative ways to solve non-perturbative scale-dependent renormalization problems. The goal is to develop a framework that will allow us to improve significantly our control over the current most relevant uncertainties. In this section, we thus want to recall what the main challenges are in solving this class of problems and which are the common approaches that are used to tackle them. Many of the concepts and observations that will be presented are for the most part well known. However, these issues are now more current than ever given the high precision that lattice QCD calculations have achieved, in particular in the determination of the QCD parameters. For this reason, we think it is important to address them also here. This gives us the opportunity to discuss some new insight that has been gathered from several recent high-precision studies, as well as introducing key concepts for later sections.

As anticipated, the discussion will focus on the case of the QCD coupling $\alpha _{\mathrm{s}}$. This allow us to analyze in easier terms the main challenges that we need to face in high-precision non-perturbative determinations of RG runnings while capturing all the relevant issues. Moreover, lattice determinations of the strong coupling are a distinct case of competitive calculations which have the potential to deliver unprecedentedly precise results for a very relevant and fundamental quantity. Making a significant progress over the present state-of-the-art determinations by mere brute force, however, is extremely demanding from the computational point of view. It is therefore mandatory to develop new strategies with the clear scope of improving our control on all sources of uncertainty.

### Determinations of $\alpha _{\mathrm{s}}$ on and off the lattice

As already mentioned, since more than a decade lattice QCD is providing the Particle Physics community with the most accurate determinations of $\alpha _{\mathrm{s}}$ (see Refs. [3–5]). The reason behind this is that, as we shall recall, lattice determinations have some important advantages over their phenomenological counterparts (see e.g. Refs. [2, 6, 15] for some reviews).

Any determination of $\alpha _{\mathrm{s}}$, whether on the lattice or not, relies on the following basic strategy. One considers a short-distance observable ${{\mathcal {O}}}(q)$ which depends on a characteristic energy scale *q*. In the limit where $q\rightarrow \infty$, this observable is compared with its theoretical prediction, ${{\mathcal {O}}}_{\mathrm{th}}(q)$, in terms of a perturbative expansion^2^

1 $$\begin{aligned} {{\mathcal {O}}}_{\mathrm{th}}(q)\!=\!\sum _{n=0}^N k_n(s)\alpha _{\mathrm{s}}^n(\mu ) \!+\!\mathrm{O}(\alpha _{\mathrm{s}}^{N+1})\!+\!\mathrm{O}\bigg ({\varLambda ^p\over q^p}\bigg ), \ \mu \!=\!{q\over s}\,.\nonumber \\ \end{aligned}$$

The functions $k_n(s)$ appearing in this equation are the coefficient functions defining the perturbative series. They are known up to some order *N* and depend on the scale factor, $s>0$, that relates the renormalization scale $\mu$ at which $\alpha _{\mathrm{s}}$ is extracted with the scale *q*. The basic difference between phenomenological and lattice determinations of $\alpha _{\mathrm{s}}$ is the choice of the observable ${{\mathcal {O}}}(q)$.

Requiring ${{\mathcal {O}}}_{\mathrm{th}}(q)={{\mathcal {O}}}(q)$ for some finite *q*, clearly fixes the value of $\alpha _{\mathrm{s}}(\mu )$ only up to some error. This error comes from several different sources, many of which are common to all types of determinations. First of all, there is the precision $\delta {{\mathcal {O}}}(q)$ to which the observable ${{\mathcal {O}}}(q)$ is known. This of course depends on the relevant statistical and systematic uncertainties associated with the determination of ${{\mathcal {O}}}(q)$. Secondly, there is the effect of the truncation of the perturbative series to a given order, i.e. the size of the $\mathrm{O}(\alpha _{\mathrm{s}}^{N+1})$ terms in Eq. (1). In addition to these there are contaminations from “non-perturbative contributions”. These are represented in Eq. (1) by power corrections to the perturbative expansion of $\mathrm{O}(\varLambda ^p/q^p)$, where $p>0$ and $\varLambda$ is some characteristic non-perturbative scale of QCD.^3^ Thus, regardless of the chosen strategy, an accurate determination of $\alpha _{\mathrm{s}}$ needs to have, *at least*, these general sources of error under control. Note that for the most part these are systematic in nature.

Lattice determinations of $\alpha _{\mathrm{s}}$ are in principle favored in several ways in succeeding at this task. Firstly, on the lattice the QCD parameters are first renormalized in terms of some precisely measured hadronic quantities (e.g. hadron masses, decay constants, etc.), for which experimental uncertainties typically contribute only marginally to the end result. Once these are fixed, one has lots of freedom in choosing an observable ${{\mathcal {O}}}(q)$ as the getaway to extract $\alpha _{\mathrm{s}}$. One can therefore *devise* convenient observables which have small statistical and systematic uncertainties; in particular there is no need for these quantities to be accessible in experiments. Phenomenological determinations of $\alpha _{\mathrm{s}}$, instead, rely on experimental data for the observable ${{\mathcal {O}}}(q)$. It is the typical situation that when *q* becomes large, and therefore many sources of systematic uncertainty in Eq. (1) become small, the experimental errors $\delta {{\mathcal {O}}}(q)$ become large. It is thus difficult to find in general a single experimental quantity ${{\mathcal {O}}}(q)$ that allows one to accurately follow its scale dependence over a wide range of *q*-values. On the lattice, on the other hand, if *carefully* chosen, ${{\mathcal {O}}}(q)$ can be computed precisely from low- up to very high-energy scales. This gives a unique handle on controlling non-perturbative corrections and the contribution of the missing perturbative orders in Eq. (1).

Another advantage for the lattice theorist is that ${{\mathcal {O}}}$ is defined within QCD alone. Consequently, the theoretical description ${{\mathcal {O}}}_{\mathrm{th}}$ of Eq. (1) does not need to include contributions besides those from QCD. In addition, no modeling of hadronization is needed when comparing the observable ${{\mathcal {O}}}$ with its perturbative prediction ${{\mathcal {O}}}_{\mathrm{th}}$. Different is again the situation for phenomenological determinations. In these cases, other Standard Model (SM) contributions may be needed in order to extract $\alpha _{\mathrm{s}}$ and some modeling of hadronization is necessary. Depending on the process, these are known only up to some accuracy and typically depend on the value of other SM parameters as well. The precision one can aim for $\alpha _{\mathrm{s}}$ can therefore be limited by these factors.^4^ Of course, lattice QCD determinations are not entirely exempted from this issue. In this case, however, the problem of disentangling the QCD contributions from “the rest” is confined to the hadronic quantities entering the renormalization of the theory, rather than to the observable ${{\mathcal {O}}}$ itself. As mentioned earlier, the uncertainties on the hadronic quantities have, at present, limited impact on the results for $\alpha _{\mathrm{s}}$.

All current lattice QCD determinations of $\alpha _{\mathrm{s}}$, on the other hand, have to deal with the fact that their calculations are performed with an unphysical number of quark flavors. The bottom and typically also the charm quark are in fact not included in the simulations. This brings up the issue of having to account for their missing contributions. We shall leave this very important discussion aside for the moment and come back to it in detail in the following (see Sect. 3).

### The challenge of reaching high energy

Having for the most part presented the pros that lattice determinations of $\alpha _{\mathrm{s}}$ in principle have, we now address what the main difficulties are in practice.

As any lattice QCD observable, besides the statistical uncertainties, ${{\mathcal {O}}}(q)$ is affected by several systematics that need to be controlled. These include general ones, i.e. discretization errors, finite-volume effects, an unphysical number of quarks, and quark-mass effects, as well as others which depend on the specific choice of ${{\mathcal {O}}}$ and set-up that we make (e.g. excited-state contaminations, finite-temperature effects, Gribov copies, topology freezing, etc.). Finite quark-mass effects are typically not a relevant issue in determinations of the QCD coupling [3]. As anticipated, we then leave the problem of having an unphysical quark-content for later. We also ignore observable specific issues. Here we focus instead on discretization and finite-volume effects. The combination of having the two under control, in fact, can severely restrict the accessible range of *q*-values, if the renormalization strategy is not carefully chosen.

In particular, if one is determined in resolving within the same lattice simulation both the hadronic energy scales relevant for the renormalization of the lattice theory, and the energy scale *q* at which $\alpha _{\mathrm{s}}$ is extracted, then one is necessarily limited by the simultaneous constraints:

2 $$\begin{aligned} L^{-1}\ll \varLambda \quad \text {and} \quad \varLambda \ll q\ll a^{-1}\,. \end{aligned}$$

The first inequality expresses the fact that finite-volume effects must be under control in hadronic quantities. The infrared cutoff set by the finite extent *L* of the lattice must be smaller than the typical non-perturbative scales of QCD, denoted here by $\varLambda$. The second inequality, instead, encodes two separate requirements. On the one hand, the scale *q* must be much lower than the ultraviolet cutoff set by the lattice spacing *a*. In this way, discretization errors in ${{\mathcal {O}}}(q)$ are kept under control, and ${{\mathcal {O}}}(q)$ can be obtained in the continuum limit with controlled errors.^5^ On the other hand, *q* needs to be much larger than the scales $\varLambda$. Only in this situation perturbation theory can reliably be applied to extract $\alpha _{\mathrm{s}}$ by comparing ${{\mathcal {O}}}(q)$ with Eq. (1).

The typical lattices that are simulated today have sizes $L/a\lesssim 100$. Taking for definiteness $m_\pi L=4$, with $m_\pi =140\,\mathrm{MeV}$, the first condition in Eq. (2) implies that for such ensembles $q \ll a^{-1}\approx 3.5\,\mathrm{GeV}$. Of course this is a crude estimate and somewhat higher energies might be achieved by compromising at different stages of the calculation (e.g.  considering heavier pion masses or smaller volumes in order to reach smaller values of *a*, or taking $aq\lesssim 1$). Nonetheless, it is clear that although convenient in practice, considering lattices devised for studying hadronic physics to compute short-distance quantities necessarily poses severe challenges on the feasibility of the approach, as the accessible energies *q* are quite limited.

As a concrete example of this situation, we can point to the recent determination of $\alpha _{\mathrm{s}}$ of Ref. [19]. For their computation the authors employ state-of-the-art large-volume simulations by the CLS initiative [20, 21]. The two-point functions of both axial and vector currents at short-distances are used to extract $\alpha _{\mathrm{s}}$. Given the range of lattice spacings at their disposal, $a\approx 0.04-0.08\,\mathrm{fm}$, the accessible energies for which continuum limit extrapolations could be performed in a controlled way are limited to $q\lesssim 2\,\mathrm{GeV}$.

As we shall see below with explicit examples, a limited range of low *q*-values unavoidably implies a limited attainable precision for $\alpha _{\mathrm{s}}$ as perturbative truncation errors become a major issue.

#### The finite volume is your friend

In order to reach high precision we must tailor the lattice simulations to the problem at hand. Going back to Eq. (2), there is in fact no reason to try to satisfy simultaneously the two conditions as these belong to separate problems. On the one hand, there is the determination of the low-energy quantities used for the hadronic renormalization of the lattice theory, while, on the other hand, there is the determination of ${{\mathcal {O}}}(q)$ for large *q*.

A more natural strategy is therefore to split the problem over several sets of lattice simulations, each one covering a different range of energy scales. In this way, systematic effects can be more easily kept under control, as the relevant conditions will be milder for each individual set of simulations. The way to effectively achieve this in practice is to employ what are known as *finite-volume renormalization schemes* [11]. In this case, the scale *q* at which the observable ${{\mathcal {O}}}(q)$ is evaluated is identified with the inverse linear extent of the finite volume, i.e. $q=L^{-1}$. One may say that, in fact, the observable ${{\mathcal {O}}}$ considered is a *finite-volume effect*. With this choice, one computes the non-perturbative RG running of ${{\mathcal {O}}}(L^{-1})$ by simulating lattices with different physical extent *L*. This strategy goes under the name of *finite-size (or step-)scaling* [11, 12] (see Ref. [2] for a recent account).

More precisely, having fixed the bare QCD parameters through some hadronic quantities, one computes ${{\mathcal {O}}}(L^{-1}_{\mathrm{had}})$ at a low-energy scale $q_{\mathrm{had}}\equiv L^{-1}_{\mathrm{had}} \approx \varLambda$, and determines $L_{\mathrm{had}}$ in physical units. This is achieved by computing $\lim _{a\rightarrow 0}(am_{\mathrm{had}})(L_\mathrm{had}/a)=\mathrm{O}(1)$, where $m_{\mathrm{had}}$ is a known low-energy scale. No large scale separations are involved in this step, and at common bare parameters one can satisfy the conditions $L_\mathrm{had}/a\gg 1$ and $am_{\mathrm{had}}\ll 1$, as well as having finite-volume effects in $m_{\mathrm{had}}$ under control.

Secondly, one computes in the continuum limit the change in ${{\mathcal {O}}}(L^{-1})$ as *L* is varied by a known factor, say, $L\rightarrow L/2$. This step is repeated a number of times *n*, going from each new *L* to the next one. Once the energy scale reached, $q_n=2^n/L_\mathrm{had}$, is large compared to the hadronic scales, perturbation theory can safely be applied to extract $\alpha _{\mathrm{s}}(\mu _{\mathrm{PT}}=q_n/s)$ from the value of ${{\mathcal {O}}}(q_n)$ (cf. Eq. (1)).

It is important to emphasize that, if carefully chosen, the only source of systematic errors that affect the determination of ${{\mathcal {O}}}(L^{-1})$ are discretization effects. In particular, no matter what the scale $q=L^{-1}$ is, discretization effects are under control once $L^{-1}=q\ll a^{-1}$, i.e. $L/a\gg 1$. This approach elegantly exploits the freedom that we have in lattice QCD in choosing the observable ${{\mathcal {O}}}(q)$ to completely circumvent the issue of necessarily having a finite volume. In particular, within this strategy the computational power is entirely invested into controlling a single systematic uncertainty.

In principle there is quite some freedom in choosing the finite-volume observable ${{\mathcal {O}}}(q)$. For the strategy to be successful in practice, however, this should have a number of desirable properties (see e.g. Ref. [2]). First of all, it should be easily and precisely measurable in Monte Carlo simulations. It should be computable in perturbation theory to a sufficiently high-loop order in order to guarantee good precision in extracting $\alpha _{\mathrm{s}}$ through Eq. (1). It should preferably be gauge invariant, in order to avoid issues with Gribov copies once studied non-perturbatively, and also be directly measurable for zero quark masses (see below). Finally, it should have, in general, small lattice artifacts. In fact, it is not straightforward to find a single observable that has all these nice features for any range of *q*-values. This, however, is not a real issue as different observables may be accurately combined in order to cover all the relevant range of scales *q*. We shall see explicit examples of good complementary observables in forthcoming sections.

#### $\varLambda$-parameters, $\beta$-functions, and all that

As we leave the general discussion for entering a more quantitative analysis of the challenges of extracting $\alpha _{\mathrm{s}}$, we find convenient to reformulate the problem in slightly different terms. First of all, it is useful to associate with the observable ${{\mathcal {O}}}$ a renormalized coupling ${\bar{g}}_{{\mathcal {O}}}$ through the relation:

3 $$\begin{aligned}&\alpha _{{\mathcal {O}}}(\mu )\equiv {{\bar{g}}^2_{{\mathcal {O}}}(\mu )\over 4\pi } \equiv {{{\mathcal {O}}}(\mu ) -k_0\over k_1} \overset{\mu \rightarrow \infty }{=} \alpha _{\mathrm{s}}(\mu )\nonumber \\&\quad + {k_2\over k_1}\alpha _{\mathrm{s}}^{2}(\mu )+\ldots \,, \end{aligned}$$

where the coefficients $k_i\equiv k_i(1)$ are those appearing in the perturbative expansion, Eq. (1). This simple procedure defines a non-perturbative, regularization-independent, QCD coupling. In terms of these couplings, the extraction of $\alpha _{\mathrm{s}}$ is interpreted as the perturbative matching between $\alpha _{{{\mathcal {O}}}}$ and $\alpha _{\mathrm{s}}$, where different observables ${{\mathcal {O}}}$ define different renormalization schemes. The common normalization allows us to compare the value of the couplings in different schemes as they approach the high-energy limit. This is useful in assessing the regime of applicability of the perturbative matching as the latter can be characterized by the value that $\alpha _{{{\mathcal {O}}}}$ should reach.

We recall at this point that the non-perturbative couplings studied within lattice QCD are implicitly defined for a given number of quark flavors, ${N_{\mathrm{f}}}$. A more proper notation to use for the couplings is therefore $\alpha _{{{\mathcal {O}}}}^{({N_{\mathrm{f}}})}(\mu )\equiv [{\bar{g}}^{({N_{\mathrm{f}}})}_{{\mathcal {O}}}(\mu )]^2/(4\pi )$, which emphasizes the fact that ${\bar{g}}^{({N_{\mathrm{f}}})}_{{\mathcal {O}}}(\mu )$ must be considered as a coupling within the ${N_{\mathrm{f}}}$-flavor theory. For ease of notation, however, in this section we will often take the liberty of dropping the superscripts ${N_{\mathrm{f}}}$ and leave these understood, unless they are needed to avoid any confusion or for later reference.

Having this noticed, a particularly convenient class of schemes to consider are *mass-independent* (or simply massless) renormalization schemes [22]. These are defined in terms of observables ${{\mathcal {O}}}$ evaluated for vanishing quark masses. As a result, the RG running of these couplings is decoupled from that of the renormalized quark masses and therefore simpler to solve. On the other hand, differently from the physical case of massive schemes, quarks do not decouple in the RG running of massless schemes [23, 24]. Hence, in the ${N_{\mathrm{f}}}$-flavor theory the latter is characterized by a fixed number of active flavors corresponding to ${N_{\mathrm{f}}}$.^6^

To the coupling ${\bar{g}}^{({N_{\mathrm{f}}})}_{{\mathcal {O}}}$ in a given mass-independent scheme we can associate a quark-mass independent $\varLambda$-parameter, $\varLambda ^{({N_{\mathrm{f}}})}_{{\mathcal {O}}}$, defined as,

4 $$\begin{aligned} \begin{aligned} \varLambda ^{({N_{\mathrm{f}}})}_{{\mathcal {O}}}&=\mu \,\varphi ^{({N_{\mathrm{f}}})}_{\mathrm{g,{{\mathcal {O}}}}} ({\bar{g}}^{({N_{\mathrm{f}}})}_{{\mathcal {O}}}(\mu ))\,, \\[1ex] \varphi ^{({N_{\mathrm{f}}})}_{\mathrm{g,{{\mathcal {O}}}}}({\bar{g}})&= (b_0({N_{\mathrm{f}}}){\bar{g}}^2)^{-{b_1({N_{\mathrm{f}}})\over 2b_0({N_{\mathrm{f}}})^2}}\, e^{-{1\over 2b_0({N_{\mathrm{f}}}){\bar{g}}^2}}\times \\[1ex]&\times \exp \bigg \{\!\! -\!\int _{0}^{{\bar{g}}} \mathrm{d}g \bigg [{1\over \beta ^{({N_{\mathrm{f}}})}_{{\mathcal {O}}}(g) } \!+\! {1\over b_0({N_{\mathrm{f}}}) g^3} - {b_1({N_{\mathrm{f}}})\over b_0({N_{\mathrm{f}}})^2 g}\bigg ]\bigg \}\,. \end{aligned} \end{aligned}$$

Through this relation, the value of the coupling ${\bar{g}}^{({N_{\mathrm{f}}})}_{{\mathcal {O}}}(\mu )$ at any renormalization scale $\mu$ is in one-to-one correspondence with $\varLambda ^{({N_{\mathrm{f}}})}_{{\mathcal {O}}}$, provided that the $\beta$-function,

5 $$\begin{aligned} \beta ^{({N_{\mathrm{f}}})}_{{\mathcal {O}}}({\bar{g}}) \equiv \mu {\mathrm{d}{\bar{g}}^{({N_{\mathrm{f}}})}_{{\mathcal {O}}}(\mu )\over \mathrm{d}\mu }\bigg |_{{\bar{g}}}\,, \end{aligned}$$

is known. The $\beta$-function describes the dependence of the coupling on the renormalization scale. In perturbation theory, it has an expansion which at the *N*-loop order reads:

6 $$\begin{aligned} \beta _{{\mathcal {O}}}^{({N_{\mathrm{f}}})\,\mathrm{PT}}({\bar{g}})\equiv -{\bar{g}}^3\sum _{k=0}^{N-1} b_k({N_{\mathrm{f}}})\,{\bar{g}}^{2k}\,, \end{aligned}$$

with

7 $$\begin{aligned} b_0({N_{\mathrm{f}}})= & {} {1\over (4\pi )^{2}}\bigg (11-{2\over 3}{N_{\mathrm{f}}}\bigg )\,,\nonumber \\ b_1({N_{\mathrm{f}}})= & {} {1\over (4\pi )^{4}}\bigg (102-{38\over 3}{N_{\mathrm{f}}}\bigg )\,. \end{aligned}$$

The coefficients $b_0({N_{\mathrm{f}}}),b_1({N_{\mathrm{f}}})$ are universal and shared by all mass-independent renormalization schemes. The scheme dependence only enters through the higher-order coefficients, $b_i({N_{\mathrm{f}}})\equiv b^{{\mathcal {O}}}_i({N_{\mathrm{f}}})$, with $i\ge 2$.

A first compelling property of $\varLambda$-parameters is that, differently from the case of couplings, their scheme dependence is in fact trivial. Leaving the ${N_{\mathrm{f}}}$-dependence implicit and taking the $\varLambda$-parameter in the $\overline{\mathrm{MS}}$-scheme, $\varLambda _{\overline{\mathrm{MS}}}$, as reference, we have that any other $\varLambda$-parameter $\varLambda _{{{\mathcal {O}}}}$ is *exactly* related to $\varLambda _{\overline{\mathrm{MS}}}$ by the relation:

8 $$\begin{aligned} {\varLambda _{\overline{\mathrm{MS}}}\over \varLambda _{{{\mathcal {O}}}}}= \exp \bigg \{{c_1(1)\over 2b_0}\bigg \}\,. \end{aligned}$$

In this equation $c_1(1)$ is the 1-loop coefficient of the perturbative matching relation between the corresponding couplings, which at *M*-loop order reads,

9 $$\begin{aligned} {\bar{g}}^2_{\overline{\mathrm{MS}}}(s\mu )= {\bar{g}}^{2}_{{{\mathcal {O}}}}(\mu ) \Bigg (1+ \sum _{k=1}^{M} c_k(s) {\bar{g}}^{2k}_{{{\mathcal {O}}}}(\mu )\Bigg )\,, \quad s>0\,. \end{aligned}$$

It is clear that the $\varLambda$-parameters are non-perturbatively defined once the corresponding couplings and $\beta$-functions are.^7^ In addition, they are *exact* solutions of the Callan-Symanzik equations [25–27], and therefore RG invariants, i.e. $\mathrm{d}\varLambda _{{\mathcal {O}}}/\mathrm{d}\mu =0$. From a non-perturbative perspective this makes the $\varLambda$-parameters natural quantities to compute, as they provide reference scales for both the low- and high-energy regimes of QCD. We finally stress that, as their corresponding couplings, the $\varLambda$-parameters are defined for a given number of quark flavors, ${N_{\mathrm{f}}}$ (cf. Eq. (4)). Each ${N_{\mathrm{f}}}$-flavor theory therefore has its own $\varLambda$-parameters.

#### Systematic uncertainties in extracting $\varLambda ^{({N_{\mathrm{f}}})}_{\overline{\mathrm{MS}}}$

Once the overall energy scale of the given ${N_{\mathrm{f}}}$-flavor theory has been set,^8^ the non-perturbative value of the coupling, ${\bar{g}}_{\mathrm{PT}}\equiv {\bar{g}}_{{\mathcal {O}}}(\mu _{\mathrm{PT}})$, in any scheme, at some high-energy scale $\mu _{\mathrm{PT}}$, allows for estimating $\varLambda _{\overline{\mathrm{MS}}}^{({N_{\mathrm{f}}})}$. Below we present two commonly employed strategies.

**Strategy 1.** Using Eq. (4) we first get an *asymptotic* estimate for $\varLambda _{{{\mathcal {O}}}}/\mu _{\mathrm{{PT}}}$ as,

10 $$\begin{aligned} {\varLambda _{{{\mathcal {O}}}}\over \mu _{\mathrm{PT}}} = \varphi _{\mathrm{g,{{\mathcal {O}}}}}({\bar{g}}_{\mathrm{PT}}) \overset{{\bar{g}}_\mathrm{PT}\rightarrow 0}{\approx } \varphi ^{\mathrm{PT}}_{\mathrm{g,{{\mathcal {O}}}}} ({\bar{g}}_{\mathrm{PT}})+ \mathrm{O}\big ({\bar{g}}_{\mathrm{PT}}^{2N-2}\big )\,. \end{aligned}$$

In this relation, $\varphi ^{\mathrm{PT}}_{\mathrm{g,{{\mathcal {O}}}}}$ is defined analogously to $\varphi _{\mathrm{g,{{\mathcal {O}}}}}$ of Eq. (4) but with the replacement $\beta _{{\mathcal {O}}}\rightarrow \beta _{{\mathcal {O}}}^{\mathrm{PT}}$, with $\beta _{{\mathcal {O}}}^{\mathrm{PT}}$ the perturbative $\beta$-function of Eq. (6). From the estimate of ${\varLambda _{{{\mathcal {O}}}}/\mu _{\mathrm{PT}}}$, ${\varLambda _{\overline{\mathrm{MS}}}/\mu _{\mathrm{PT}}}$ is obtained, with no further approximation, using Eq. (8). Finally, the knowledge of $\mu _{\mathrm{{PT}}}$ in physical units gives us $\varLambda _{\overline{\mathrm{MS}}}$.

As anticipated by Eq. (10), due to the truncation of $\beta _{{\mathcal {O}}}$ to *N*-loop order in the evaluation of ${\varLambda _{{{\mathcal {O}}}}/\mu _{\mathrm{PT}}}$, our estimate for ${\varLambda _{\overline{\mathrm{MS}}}/\mu _{\mathrm{PT}}}$, comes with a systematic uncertainty of $\mathrm{O}\big ({\bar{g}}_\mathrm{PT}^{2N-2}\big )$. It is important to stress that Eq. (10) is in fact only asymptotic. Similarly to what we discussed about Eq. (1), our estimate for ${\varLambda _{\overline{\mathrm{MS}}}/\mu _{\mathrm{PT}}}$ is in principle also affected by “non-perturbative corrections”, if the coupling ${\bar{g}}_{\mathrm{PT}}$ is not small enough for perturbation theory to be in the regime of applicability. We shall come back to this issue shortly.

**Strategy 2.** A second possibility to estimate $\varLambda _{\overline{\mathrm{MS}}}$ is to first obtain from the non-perturbative value of ${\bar{g}}_{\mathrm{PT}}$ an estimate for ${\bar{g}}_{\overline{\mathrm{MS}}}(s\mu _{\mathrm{{PT}}})$ using the *M*-loop relation, Eq. (9). Given this, we can estimate ${\varLambda _{\overline{\mathrm{MS}}}/(s\mu _{\mathrm{PT}}})$ using Eq. (4) and the perturbative expression for the $\beta$-function, Eq. (6), in the $\overline{\mathrm{MS}}$-scheme. As before, the knowledge of $\mu _\mathrm{{PT}}$ in physical units then gives us $\varLambda _{\overline{\mathrm{MS}}}$.

In order to establish the perturbative uncertainties associated with this second approach, we first recall that the $\beta$-function in the $\overline{\mathrm{MS}}$-scheme is currently known to 5-loop order [28–30]. This introduces a systematic uncertainty in the determination of ${\varLambda _{\overline{\mathrm{MS}}}/\mu _{\mathrm{PT}}}$ of $\mathrm{O}\big ({\bar{g}}^8_{\overline{\mathrm{MS}}}(s\mu _{\mathrm{{PT}}})\big ) \approx \mathrm{O}\big ({\bar{g}}_{\mathrm{PT}}^{8}\big )$ (cf. Eqs. (10) and (9)). Secondly, using Eq. (4) it is easy to show that the perturbative matching at *M*-loop order between ${\bar{g}}_\mathrm{PT}$ and ${\bar{g}}_{\overline{\mathrm{MS}}}(s\mu _{\mathrm{{PT}}})$ translates into a systematic uncertainty in ${\varLambda _{\overline{\mathrm{MS}}}/\mu _{\mathrm{PT}}}$ of $\mathrm{O}\big ({\bar{g}}_{\mathrm{{PT}}}^{2M}\big )$.

For all schemes ${\bar{g}}_{{{\mathcal {O}}}}$ used in lattice QCD determinations of $\varLambda _{\overline{\mathrm{MS}}}$ we have that $M\le 3$ (see e.g. Ref. [3]). The systematic errors coming from the matching between couplings is therefore parametrically larger than the one from the truncation of $\beta _{\overline{\mathrm{MS}}}$. Moreover, note that for all these schemes we have that $M=N-1$, where *N* is the loop order at which the corresponding perturbative $\beta$-function, $\beta ^{\mathrm{PT}}_{{\mathcal {O}}}$, is known (cf. Eq. (6)).^9^ This means that, for the schemes ${\bar{g}}_{{\mathcal {O}}}$ commonly used, **Strategy 1.** and **2.** result in the *same* parametric uncertainties of $\mathrm{O}\big ({\bar{g}}_\mathrm{{PT}}^{2N-2}\big )$. Clearly, although parametrically the same, the actual size of the corrections might be different. In this second strategy, in particular, when matching the couplings we have the freedom to choose the parameter *s* (cf. Eq. (9)). Different choices can result in different perturbative corrections to ${\varLambda _{\overline{\mathrm{MS}}}/\mu _{\mathrm{PT}}}$.

Devising different strategies like the ones above and comparing their outcome can help us assessing the systematic uncertainties in $\varLambda _{\overline{\mathrm{MS}}}$ coming from the use of perturbation theory at $\mu _{\mathrm{{PT}}}$. A truly systematic study, however, requires to compare the determination of $\varLambda _{\overline{\mathrm{MS}}}/\mu _{\mathrm{ref}}$, where $\mu _{\mathrm{ref}}$ is a common reference scale, for several different values of ${\bar{g}}_{\mathrm{PT}}$ as ${\bar{g}}_{\mathrm{PT}}\rightarrow 0$. Only if agreement is found among all determinations, possibly including different strategies, one may be reassured that $\mathrm{O}\big ({\bar{g}}_{\mathrm{PT}}^{2N-2}\big )$ terms, as well as non-analytic terms in the coupling, are negligible within the statistical uncertainties. In the case where, instead, the results for $\varLambda _{\overline{\mathrm{MS}}}/\mu _{\mathrm{ref}}$ show a clear dependence on ${\bar{g}}_{\mathrm{PT}}$, one should first confirm that this is actually compatible with the expected $\mathrm{O}\big ({\bar{g}}_\mathrm{PT}^{2N-2}\big )$ corrections. If this is the case, one may be confident that the asymptotic regime of the perturbative expansion is reached, no “non-perturbative corrections” are relevant, and one can therefore take as final estimate for $\varLambda _{\overline{\mathrm{MS}}}/\mu _{\mathrm{ref}}$ the extrapolated result for ${\bar{g}}_\mathrm{PT}\rightarrow 0$.

Clearly, the program above is ambitious. The running of the coupling ${\bar{g}}_{\mathrm{PT}}$ at high energies is only logarithmic in $\mu _\mathrm{PT}/\varLambda _{{\mathcal {O}}}$. Reducing the size of the perturbative truncation errors by a given factor hence requires an *exponentially* larger change in the energy scale $\mu _{\mathrm{{PT}}}$. In order to accurately estimate the systematic uncertainties coming from the use of perturbation theory one therefore needs to cover, non-perturbatively, a wide range of energies, reaching up to very high scales.

If the chosen strategy to determine $\varLambda _{\overline{\mathrm{MS}}}$ does not allow for this and the accessible range of ${\bar{g}}_\mathrm{PT}$ is quite limited, one might be tempted to estimate the uncertainties due to the application of perturbation theory in more simplistic ways. For example, one might opt for simply adding to the final result an uncertainty $\delta \varLambda _{\overline{\mathrm{MS}}}/\varLambda _{\overline{\mathrm{MS}}}= k\,{\bar{g}}_{\mathrm{PT}}^{2N-2}$, where *k* is estimated in some way from the available perturbative information. Alternatively, one might estimate these uncertainties based on the spread of the results obtained at the smallest available ${\bar{g}}_{\mathrm{{PT}}}$ from different strategies (e.g. **Strategy 1.** vs **Strategy 2.**). Given the asymptotic nature of the perturbative expansion, however, these practices cannot be considered reliable in general. From the very definition of asymptotic series the only reliable way to assess its accuracy is to compare the series with the full function as ${\bar{g}}_\mathrm{PT}\rightarrow 0$.^10^ In order to do so, the coupling ${\bar{g}}_{\mathrm{PT}}$ must be varied by a sensible amount reaching down to small values.

For the same reasons, it is not advisable to estimate the size of “non-perturbative corrections” using some model assumption, or use some model to extrapolate the results for $\varLambda _{\overline{\mathrm{MS}}}/\mu _{\mathrm{ref}}$ to ${\bar{g}}_{\mathrm{PT}}\rightarrow 0$. Our knowledge of the form of non-perturbative effects is rather limited and the separation between what is perturbative and non-perturbative is all but well defined. Hence, it is always debatable whether any model that tries to capture non-analytic terms in the coupling is really adequate to describe the data within the given accuracy. Moreover, if the coupling ${\bar{g}}_{\mathrm{PT}}$ cannot be varied much, it is difficult to really distinguish, e.g. a power correction, from some higher-order term in ${\bar{g}}_{\mathrm{PT}}$, when statistical errors and other uncertainties are present. A more reliable practice is thus to avoid regions of large ${\bar{g}}_{\mathrm{PT}}$ where the $\mathrm{O}\big ({\bar{g}}_{\mathrm{PT}}^{2N-2}\big )$ behavior has not clearly set in.

### The accuracy of perturbation theory at high energy

In this section we want to review some recent determinations of $\varLambda _{\overline{\mathrm{MS}}}$ which paid particular attention to the issue of the accuracy of perturbation theory in extracting $\varLambda _{\overline{\mathrm{MS}}}$ [31–35]. As we shall see, the concerns exposed in the previous sections are legitimate once the precision goals become competitive. A robust analysis of perturbative truncation errors is essential to reach high accuracy.

#### The high-energy regime of ${N_{\mathrm{f}}}=3$ QCD

**Fig. 1:**
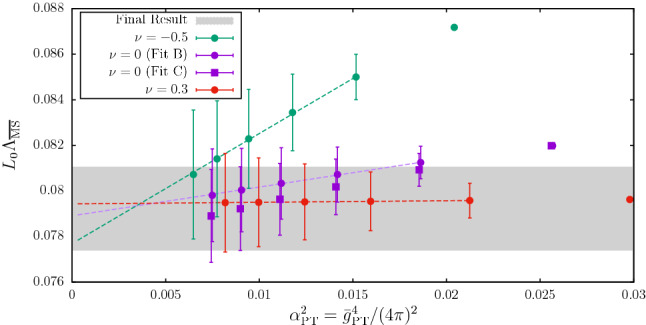
Determination of $\varLambda _{\overline{\mathrm{MS}}}^{(3)}$ in units of $\mu _{\mathrm{ref}}=L_0^{-1}$ for different values of $\alpha _{\mathrm{PT}}$ [32]. The extraction in different $\mathrm{SF}_\nu$-schemes $(\nu =-0.5,0,0.3)$ is shown, as well as a comparison with the final result $\varLambda ^{(3)}_{\overline{\mathrm{MS}}}/ \mu _\mathrm{ref}=0.0791(19)$ [32]. As the reader can see, when the extraction is performed at high-enough energies $(\alpha _{\mathrm{PT}}\approx 0.1)$, all schemes nicely agree

We begin with the high-energy studies of Refs. [31, 32] in ${N_{\mathrm{f}}}=3$ QCD. In Fig. 1, we show the results from these references for $L_0\varLambda ^{({N_{\mathrm{f}}}=3)}_{\overline{\mathrm{MS}}}$ as a function of $\alpha ^2_{\mathrm{PT}}\equiv {\bar{g}}^4_{\mathrm{PT}}/(4\pi )^2$. In this plot, $\mu _{\mathrm{ref}}\equiv L_0^{-1}\approx 4.3\,\mathrm{GeV}$, is a convenient high-energy reference scale and, as in previous sections, ${\bar{g}}_{\mathrm{PT}}\equiv {\bar{g}}_{{{\mathcal {O}}}}(\mu _{\mathrm{{PT}}})$ is the value of the coupling in the given scheme at which perturbation theory is applied to extract $\varLambda ^{(3)}_{\overline{\mathrm{MS}}}/\mu _\mathrm{ref}$. The ratio $\varLambda ^{(3)}_{\overline{\mathrm{MS}}}/\mu _{\mathrm{ref}}$ is obtained following **Strategy 1.** of Sect. 2.2.3. The scales at which perturbation theory is used correspond to $\mu _\mathrm{{PT}}=2^n\mu _{\mathrm{ref}}$, with $n=0,\ldots ,5$, and range from about $4\,\mathrm{GeV}$ to $140\,\mathrm{GeV}$. The couplings considered in this study, ${\bar{g}}_{{\mathcal {O}}}(\mu )={\bar{g}}_{\mathrm{SF}_\nu }(L^{-1})$, belong to a family of finite-volume renormalization schemes based on the QCD Schrödinger functional (SF) [36–38]. Different schemes within the family are identified by different values of the parameter $\nu$. The precise definition of the schemes is not important and can be found in Refs. [31, 32, 39].^11^

In order to estimate $\varLambda ^{(3)}_{\overline{\mathrm{MS}}}/\mu _\mathrm{ref}$ the 3-loop approximation to the relevant $\beta$-functions, $\beta _{\mathrm{SF}_\nu }$, is used. The results for $\varLambda ^{(3)}_{\overline{\mathrm{MS}}}/\mu _{\mathrm{ref}}$ are therefore expected to show corrections of $\mathrm{O}(\alpha _{\mathrm{PT}}^2)$ as $\alpha _{\mathrm{PT}}\rightarrow 0$. It is important to note at this point that the 3-loop coefficients of the $\beta$-functions in the different $\mathrm{SF}_\nu$-schemes are given for ${N_{\mathrm{f}}}=3$ by [32, 42, 43]^12^:

11 $$\begin{aligned} (4\pi )^3 b_{2}^{\mathrm{SF}_\nu }= -(0.064(27) +\nu \times 1.259(10))\,. \end{aligned}$$

Hence, from the perturbative point of view all schemes with $|\nu |\lesssim 1$ appear to be on similar footing and the perturbative expansion of their $\beta$-functions is well behaved.

From this observation, one might naively expect that the $\mathrm{O}(\alpha _{\mathrm{PT}}^2)$ corrections to $\varLambda ^{(3)}_{\overline{\mathrm{MS}}}/ \mu _{\mathrm{ref}}$ obtained from different intermediate schemes are similar too. Going back to Fig. 1, we see that in all cases the results are well described by a $\mathrm{O}(\alpha _{\mathrm{PT}}^2)$ dependence over the whole range of investigated couplings. This is compatible with the expectation from the known leading non-analytic term in the expansion which is expected to be quite small at these couplings, i.e. $\mathrm{O}(e^{-2.6/\alpha })$ [32]. However, we clearly see a substantial difference in the size of the $\mathrm{O}(\alpha _\mathrm{PT}^2)$ corrections depending on the $\mathrm{SF}_\nu$-scheme that is considered. While one can find cases ($\nu =0.3$) where the $\mathrm{O}(\alpha _{\mathrm{PT}}^2)$ corrections are insignificant within errors, other schemes ($\nu =-0.5$) show significant corrections. The results for $\nu =-0.5$ at $\alpha _{\mathrm{PT}}\approx 0.12$, for instance, show a $7{-}8\%$ deviation from the final result $\varLambda ^{(3)}_{\overline{\mathrm{MS}}}/\mu _{\mathrm{ref}}=0.0791(19)$ quoted in Ref. [32], which corresponds to the gray band in the plot.

**Fig. 2:**
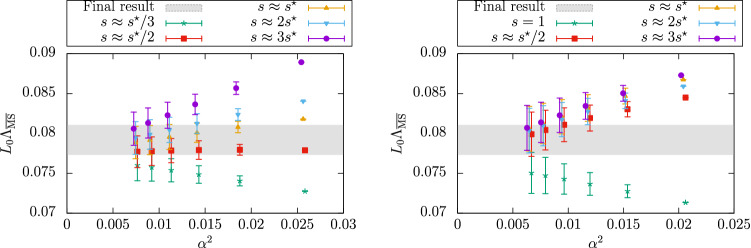
Determination of $L_0\varLambda _{\overline{\mathrm{MS}}}^{(3)}= \varLambda _{\overline{\mathrm{MS}}}^{(3)}/\mu _{\mathrm{ref}}$ at different values of $\alpha \equiv \alpha _{\mathrm{PT}}$, and using different renormalization scales (values of *s*) to match with the $\overline{\mathrm{MS}}$-scheme [32]. The left (right) panel uses the $\mathrm{SF}_\nu$-scheme with $\nu = 0$ ($\nu =-0.5$), cf. text

As the value of the coupling at which perturbation theory is applied becomes smaller, any significant difference among the different determinations of $\varLambda ^{(3)}_{\overline{\mathrm{MS}}}/\mu _{\mathrm{ref}}$ steadily fades away. In particular, once $\alpha _{\mathrm{PT}}\approx 0.1$ is reached, any difference is well below the statistical uncertainties on $\varLambda ^{(3)}_{\overline{\mathrm{MS}}}/\mu _{\mathrm{ref}}$, which at these couplings are at the level of $2-3\%$. A robust estimate for $\varLambda ^{(3)}_{\overline{\mathrm{MS}}}/\mu _{\mathrm{ref}}$ can therefore be obtained by taking its value at $\alpha _\mathrm{PT}\approx 0.1$ from one of the schemes that show milder perturbative corrections. The result $\varLambda ^{(3)}_{\overline{\mathrm{MS}}}/\mu _\mathrm{ref}=0.0791(19)$ given above, for instance, corresponds to $\mu _\mathrm{PT}=2^4\mu _{\mathrm{ref}}\approx 70\,\mathrm{GeV}$ from the $\nu =0$ scheme (cf. $\nu =0$ (fit C) in Fig. 1) [32].

The first important message from this study is that it is in fact impossible to predict the actual size of perturbative truncation errors only from the available perturbative information. To reliably assess these errors, perturbation theory must be tested against non-perturbative data over a wide range of energy scales. From the study we presented, in particular, we conclude that in order to be able to quote in full confidence the competitive precision of $2-3\%$ on $\varLambda ^{(3)}_{\overline{\mathrm{MS}}}/ \mu _{\mathrm{ref}}$, one must reach non-perturbatively $\alpha _{\mathrm{PT}}\approx 0.1$. At these couplings perturbative truncation errors are fully under control and the error on $\varLambda ^{(3)}_{\overline{\mathrm{MS}}}/\mu _{\mathrm{ref}}$ is entirely dominated by the statistical uncertainties coming from the non-perturbative running of the coupling.

It is now instructive to look at the result of the analysis of the same data according to **Strategy 2** of Sect. 2.2.3. The corresponding estimates for $\varLambda ^{(3)}_{\overline{\mathrm{MS}}}/ \mu _{\mathrm{ref}}$ are shown in Fig. 2 for the two cases, $\nu =0,-0.5$ [32]. The different determinations in each plot are obtained by varying the parameter *s* entering the perturbative matching between the $\overline{\mathrm{MS}}$-coupling and the $\mathrm{SF}_\nu$-couplings (cf. Eq. (9)). The values of *s* considered vary by about a factor $2-3$ around the value of *fastest apparent convergence*, $s^*$.^13^ In phenomenological determinations of the QCD coupling the spread of the results obtained by varying *s* around some “optimal” value, typically by a factor 2 or so, is commonly used to get an estimate of perturbative truncation errors (see e.g. Ref. [5]). Our intention is to test how this approach works in the present case.

As one can see from Fig. 2, for all choices of *s* the data show the expected $\mathrm{O}(\alpha _{\mathrm{PT}}^2)$ scaling. The slope of the data, however, can vary significantly depending on the choice of the parameter *s*. As expected, the significance of these differences is reduced as $\alpha _\mathrm{PT}\rightarrow 0$, and the different determinations come together once $\alpha _{\mathrm{PT}}\lesssim 0.1$.

What is clear from the results of Fig. 2 is that the procedure of assigning a systematic error based on the spread of the results with *s* at some fixed coupling is not always reliable. In the case of the $\nu =0$ scheme (left panel), the spread in the results between, say, $s^*/2$ and $2s^*$, encloses the final estimate (gray band in the plot) for all coupling values in the range. If this uncertainly was added to the statistical errors, it would give a conservative estimate for the total uncertainly. On the other hand, in the case of the $\nu =-0.5$ scheme (right panel), the procedure significantly underestimates the actual size of the $\mathrm{O}(\alpha _{\mathrm{PT}}^2)$ corrections. Again $\alpha _\mathrm{PT}\approx 0.1$ has to be reached for the perturbative uncertainties to be small compared to the statistical ones.

From this second analysis we reaffirm the conclusion that it is very difficult to reliably estimate perturbative truncation errors if the coupling $\alpha _{\mathrm{PT}}$ cannot be varied much, and if this is confined to values significantly larger than $\alpha _\mathrm{PT}\approx 0.1$.

#### The case of the pure Yang-Mills theory

**Fig. 3:**
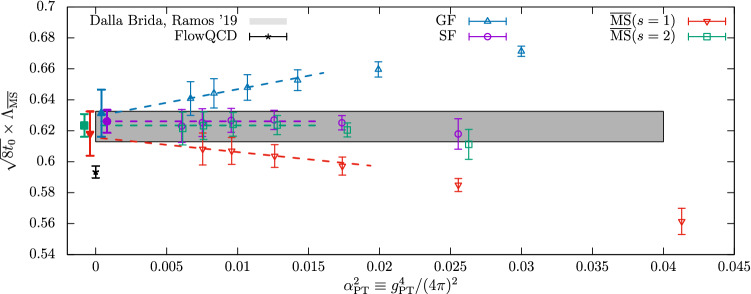
The dimensionless product $\sqrt{8t_0}\varLambda _{\overline{\mathrm{MS}}}^{(0)}$ as a function of $\alpha _{\mathrm{PT}}$ [35]. The empty symbols represent the data at the given $\alpha _{\mathrm{PT}}$, while the filled symbols are extrapolations to $\alpha _{\mathrm{PT}}\rightarrow 0$ (shifted for better readability) of the different approaches to the perturbative matching (see text for more details). The gray band is the result of Ref. [33], while the data point labeled FlowQCD is the result of Ref. [46]

**Finite-volume schemes.** The second example that we consider is taken from the recent study of Ref. [35] in the pure Yang–Mills (YM) theory. This work presents an independent analysis of the results from a previous study [33], using novel techniques. Before entering the discussion, we care to stress that the case of the pure Yang–Mills theory is not just a curious example. As we shall see in the following section, through the strategy of renormalization by decoupling precise results for $\varLambda ^{(5)}_{\overline{\mathrm{MS}}}$ can be obtained from the accurate knowledge of $\varLambda ^{(0)}_{\overline{\mathrm{MS}}}$. From this perspective, a robust determination of the $\varLambda$-parameter of the pure YM theory is very relevant.

In Fig. 3 we show the results for $\sqrt{8t_0}\varLambda ^{(0)}_{\overline{\mathrm{MS}}}$ from Ref. [35] as a function of $\alpha _{\mathrm{PT}}^2$. The scale $\mu _{\mathrm{had}}=1/\sqrt{8t_0}$ is defined in terms of the flow time $t_0$ [47], while $\alpha _{\mathrm{PT}}$ is once again the value of the relevant coupling at the renormalization scale $\mu _{\mathrm{{PT}}}$ where perturbation theory is applied. Similarly to the case of ${N_{\mathrm{f}}}=3$ QCD discussed above, different schemes and strategies have been considered in order to extract $\varLambda ^{(0)}_{\overline{\mathrm{MS}}}/\mu _{\mathrm{had}}$ and study the perturbative truncation errors. In all cases, the non-perturbative RG running from $\mu _{\mathrm{had}}$ up to $\mu _{\mathrm{{PT}}}$ is obtained using a finite-volume scheme based on the YM gradient flow (GF) [47–50]. The interested reader can find more details about the scheme in the main Refs. [33, 35] (see also Sect. 4.3).

Once $\mu _{\mathrm{{PT}}}$ is reached, $\varLambda ^{(0)}_{\overline{\mathrm{MS}}}/\mu _{\mathrm{had}}$ is estimated in a number of ways. For the case labeled as (GF) in the plot, **Strategy 1.** of Sect. 2.2.3 is employed using the 3-loop $\beta$-function in the GF-scheme of choice [51]. In the other cases, the GF-scheme is first non-perturbatively matched to the $\mathrm{SF}_{\nu =0}$-scheme introduced in the previous section. Perturbation theory is then applied either following **Strategy 1.** based on the $\mathrm{SF}_{\nu =0}$-scheme (SF label in the plot), or by following **Strategy 2.** and matching the $\mathrm{SF}_{\nu =0}$- and $\overline{\mathrm{MS}}$-coupling ($\overline{\mathrm{MS}}(s=1,2)$ in the figure). In the latter case, two values of the *s*-parameter, $s=1,2$, are studied; note that $s^*\approx 2$ in this case. In all cases, the leading parametric uncertainties in $\varLambda ^{(0)}_{\overline{\mathrm{MS}}}/\mu _{\mathrm{had}}$ from the truncation of the perturbative expansion are of $\mathrm{O}(\alpha _\mathrm{PT}^2)$.

Going back to Fig. 3 we see how two out of the four strategies ((SF) and $\overline{\mathrm{MS}}(s=2)$)) give results which are essentially independent on $\alpha _{\mathrm{PT}}$ over the whole range of couplings considered for the extraction of $\varLambda ^{(0)}_{\overline{\mathrm{MS}}}/\mu _{\mathrm{had}}$. Note that in going from the largest to the smallest couplings the energy scale varies by a factor 32 while $\alpha _{\mathrm{PT}}$ changes by about a factor 2. On the other hand, the other two types of determinations ((GF) and $\overline{\mathrm{MS}}(s=1)$)) show a significant $\alpha _\mathrm{PT}$ dependence, roughly compatible with the expected $\mathrm{O}(\alpha _{\mathrm{PT}}^2)$ scaling. What is remarkable is that even considering values of $\alpha _{\mathrm{PT}}\approx 0.08$ the different strategies give estimates for $\varLambda ^{(0)}_{\overline{\mathrm{MS}}}/\mu _{\mathrm{had}}$ which vary up to $\approx 3\%$. This is about twice as large as the statistical errors on the points (cf. Table 3 of Ref. [35]). In the case of the (GF) and ($\overline{\mathrm{MS}}(s=1)$) determinations, it is clear that a trustworthy estimate for $\varLambda ^{(0)}_{\overline{\mathrm{MS}}}/ \mu _{\mathrm{had}}$ can be quoted only by extrapolating the results for $\alpha _{\mathrm{PT}}\rightarrow 0$. In general, perturbative truncation errors are large also in the pure YM theory given the precision one can reach.

The results above show us once again the importance of an explicit non-perturbative calculation of the running of the coupling over a significant range of values, reaching down to small couplings, in order to assess the actual size of the perturbative corrections. We join the authors of Ref. [35] and conclude that only by studying non-perturbatively the limit $\alpha _{\mathrm{PT}}\rightarrow 0$ one can avoid the dangerous game of estimating perturbative uncertainties at some finite (potentially large) value of $\alpha _{\mathrm{PT}}$. Without studying this limit, the determinations can easily be affected by perturbative truncation errors, even at surprisingly small values of the coupling.

**Large-volume schemes.** A precise determination of the $\varLambda$-parameter in the pure YM theory is certainly very much facilitated from the computational point of view with respect to the case of QCD. However, as we have seen in the previous example, it is yet a non-trivial challenge to control perturbative truncation errors once a $1{-}2\%$ precision in $\varLambda _{\overline{\mathrm{MS}}}$ is reached.

The disagreement among some recent determinations of $\varLambda ^{(0)}_{\overline{\mathrm{MS}}}$ is a clear signal that these difficulties should not be underestimated. The issue is well illustrated in Fig. 3, where the very precise results labeled (FlowQCD) from Ref. [46] show a net tension with the determinations of Refs. [33, 35]. We recall that the former result is based on extracting $\varLambda ^{(0)}_{\overline{\mathrm{MS}}}/\mu _{\mathrm{had}}$ from the plaquette expectation value calculated in large-volume simulations. Bare lattice perturbation theory at couplings $\alpha _{\mathrm{PT}}\approx 0.095-0.12$ is used, with parametric uncertainties of O($\alpha _{\mathrm{PT}}^2$). We refer the reader to the given reference for the details. Here we just note that all the above determinations satisfy the most stringent criteria set by FLAG (cf. Ref. [3]). Yet, one or more of these results have underestimated uncertainties.

**Fig. 4:**
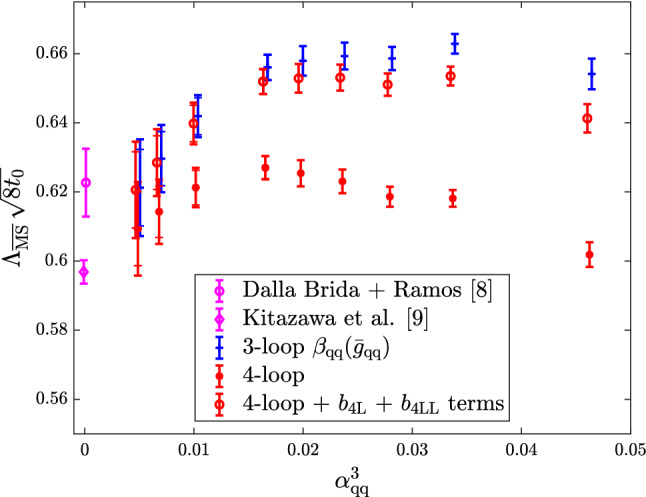
$\varLambda _{\overline{\mathrm{MS}}}^{(0)}$ in units of $\sqrt{8t_0}$ determined at various values of $\alpha _\mathrm{qq}\equiv \alpha _{\mathrm{PT}}$ [34]. The results using different orders of perturbation theory for $\beta _{\mathrm{qq}}$ are shown, as well as a comparison with the determinations of Refs. [33] and [46]. (The reference numbers in the plot are those of Ref. [34] from which the plot is taken.)

Other groups have recently engaged in a precision determination of $\varLambda ^{(0)}_{\overline{\mathrm{MS}}}$, also with the intent of resolving the disagreement above. The recent results of Ref. [34] based on the $\mathrm{qq}$-coupling, $\alpha _{\mathrm{qq}}$, defined from the static potential [52], are particularly interesting in this respect.^14^ We report them in Fig. 4. In the case of $\alpha _{\mathrm{qq}}$ the corresponding $\beta$-function, $\beta _{\mathrm{qq}}$, is known up to 4-loop order, and some partial information is available also at 5-loops (see e.g. Ref. [55]). Determinations of $\varLambda ^{(0)}_{\overline{\mathrm{MS}}}/\mu _{\mathrm{had}}$ from $\alpha _\mathrm{qq}$ are hence expected to have asymptotically $\mathrm{O}(\alpha _\mathrm{qq}^3)$ corrections. In the plot, $\alpha _{\mathrm{qq}}$ refers to the coupling at which perturbation theory is used according to **Strategy 1.** of Sect. 2.2.3, i.e. it corresponds to $\alpha _{\mathrm{PT}}$ in our previous discussions.

Despite the accurate perturbative knowledge there are a few challenges when using the $\mathrm{qq}$-scheme for precision determinations of $\varLambda _{\overline{\mathrm{MS}}}^{(0)}$ [34]. The most relevant ones for our discussion are, first of all, that the scheme is conventionally defined in an infinite space-time volume. In order to measure the coupling at small lattice spacings one therefore needs large lattice sizes to maintain the physical extent of the lattice large. In the computation of Ref. [34] lattice spacings down to $a\approx 0.01\,\mathrm{fm}$ are reached while keeping the lattice extent $L\approx 2\,\mathrm{fm}$. This means simulating lattices with up to $L/a\approx 200$. Secondly, the perturbative expansion of $\alpha _{\mathrm{qq}}$ displays some infrared divergences starting at 3-loop order in $\beta _{\mathrm{qq}}$. When resummed these give rise to terms of the form, $\alpha _{\mathrm{qq}}^n\log (\alpha _{\mathrm{qq}})^m$, $n\ge 3$, $1\le m\le n-2$, which are enhanced at small couplings (cf. Ref. [34]).

Figure 4 shows the results for $\varLambda ^{(0)}_{\overline{\mathrm{MS}}}/\mu _{\mathrm{had}}$ as a function of $\alpha _{\mathrm{qq}}^3$. The range of couplings covered by the data is $\alpha _{\mathrm{qq}}\approx 0.16-0.35$. As we can see from the plot, for couplings $\alpha _{\mathrm{qq}} > rsim 0.21$ the results for $\varLambda ^{(0)}_{\overline{\mathrm{MS}}}/\mu _{\mathrm{had}}$ have good precision, but perturbative uncertainties are large. This can be seen by looking at the difference between the 3-loop and 4-loop results (or analogously between the 4-loop and 4-loop + 5-loop log-terms results). At these large couplings, the perturbative expansion seems to have reached its limit of applicability. This severely limits the precision one can aim at for $\varLambda ^{(0)}_{\overline{\mathrm{MS}}}/\mu _{\mathrm{had}}$ if one is restricted to this range of couplings. For couplings $\alpha _\mathrm{qq}\lesssim 0.21$, the different orders of perturbation theory seem to start converging. On the other hand, the errors on the data become large. This is due to the difficulties in extrapolating the results to the continuum limit [34]. In fact, the errors are too large to make definite conclusions for the relevant limit $\alpha _{\mathrm{qq}}\rightarrow 0$.

All in all, we see from this last example that a precise determination of $\varLambda _{\overline{\mathrm{MS}}}^{(0)}$ is a challenge. Finite-volume renormalization schemes allow us to cover a wide range of couplings, reaching down to rather small values. Yet, having control on perturbative truncation errors requires care. When using large-volume schemes the situation is further complicated by controlling continuum limit extrapolations at the smallest (most relevant) couplings. Small couplings require small lattice spacings, which require large lattice sizes in order to keep the physical volume large. As a result, even in the computationally simpler case of the pure YM theory, one might have precise data confined for the most part to a region of couplings too large to have perturbative uncertainties fully under control, while at smaller couplings the data is not precise enough for a competitive determination of $\varLambda$.

### The tricky business of continuum extrapolations

Having discussed the difficulties of estimating perturbative truncation errors in precision determinations of the $\varLambda$-parameters, we now want to touch on the issue of systematic uncertainties related to the continuum limit extrapolations of the relevant couplings.

To give the reader a feeling of the pitfalls that these continuum extrapolations can conceal, we first consider the ${N_{\mathrm{f}}}=3$ results of Ref. [50]. The relevant quantity to look at in this case is the step-scaling function (SSF) of the finite-volume GF-coupling ${\bar{g}}^2_{\mathrm{GF}}(\mu )$ with SF boundary conditions (see Refs. [49, 50] and Eq. (53) for the definition of this scheme). We recall that the SSF, $\sigma (u)$, encodes the change in the coupling *u* when the renormalization scale is varied by a factor of 2 [11]. Specifically, having set $\mu =L^{-1}$,

12 $$\begin{aligned} \sigma (u)\equiv {\bar{g}}^2_{\mathrm{GF}}(\mu /2)|_{{\bar{g}}^2_\mathrm{GF}(\mu )=u}, \quad \sigma (u)=\lim _{a/L\rightarrow 0} \varSigma (a/L,u)\,.\nonumber \\ \end{aligned}$$

It is clear from its definition that the SSF is a discrete version of the $\beta$-function. The latter can in fact be obtained once the SSF is known in a range of couplings (cf. Ref. [50]).

On the lattice, the SSF is determined by extrapolating to the continuum limit its discrete approximations, $\varSigma (a/L,u)$. In order to compute the latter one must first identify a set of lattice sizes *L*/*a* and corresponding values of the bare coupling $g_0$ for which ${\bar{g}}^2_{\mathrm{GF}}(L^{-1})=u$, with *u* a specific value. The lattice SSFs $\varSigma (a/L,u)$ are then given by the couplings ${\bar{g}}^2_{\mathrm{GF}}((2L)^{-1})$ measured at the values of $g_0$ previously determined but on lattices with sizes 2*L*/*a*.

**Fig. 5:**
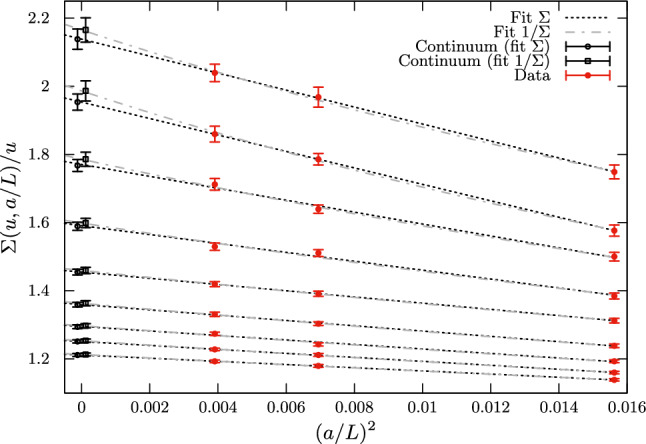
Continuum extrapolations of $\varSigma (a/L,u)/u$ for 9 different values of $u\in [2.1,6.5]$ and 3 lattice sizes $L/a=8,12,16$

The results for the lattice SSFs of the GF-coupling of Ref. [50] are shown in Fig. 5. They correspond to 9 values of the coupling $u_i\in [2.1,6.5]$, $i=1,\ldots ,9$. As one can see from the figure, the lattice data are very precise. On the other hand, discretization effects are in general large, particularly so at the largest couplings. The results for $\varSigma (u,a/L)$ vary in fact by up to 20% in the range of *L*/*a* considered, which is quite a significant change compared to the statistical errors on the points.

Given the results in Fig. 5, we may expect that a simple fit of the data linear in $(a/L)^2$ is all that is needed to extrapolate these to the continuum limit. In particular, we may consider individual continuum extrapolations for each $u_i$ value using the functional form

13 $$\begin{aligned} \varSigma (u_i,a/L)=\sigma ^{(A)}_i+{r}^{(A)}_i\times (a/L)^2 \quad \text {(fit A)}\,, \end{aligned}$$

where $\sigma ^{(A)}_i$, ${r}^{(A)}_i$ are fit parameters. Within the uncertainties, linearly in $(a/L)^2$ is in fact excellent and the above fits are very good ($\chi ^2/\mathrm{dof}\approx 0.7$). One is thus tempted to take the precise values for $\sigma ^{(A)}_i$ as estimates for the continuum SSF.

The continuum results so obtained are well described by the simple relation: $\varDelta \sigma _i^{(A)}\equiv 1/\sigma ^{(A)}_i-1/u_i\approx -0.082$. Note that this is the functional form expected from the perturbative expansion of $\sigma (u)$ at 1-loop order, although the coefficient predicted by perturbation theory is slightly different, i.e. $\approx -0.079$.^15^ This observation suggests us to perform alternative fits to the data in Fig. 5 considering the functional form

14 $$\begin{aligned} 1/\varSigma (u_i,a/L)=1/\sigma ^{(B)}_i+r^{(B)}_i\times (a/L)^2 \quad \text {(fit B)}\,, \end{aligned}$$

with $\sigma ^{(B)}_i$, ${r}^{(B)}_i$ new fit parameters. The quality of these fits is as good as for the fits A of Eq. (13). Distinguishing between the two fit forms would require significantly higher statistical precision than the present one.

It is important to note at this point that any functional form that we consider for the continuum extrapolations, necessarily comes with assumptions. Both fits A and B above, for instance, assume discretization errors of O($a^n$), $n\ge 3$, to be negligible. Moreover, even focusing only on the leading O($a^2$) effects, we know from Symanzik effective theory (SymEFT) [56–58] that these are not simply given by a “classical” term $\propto (a/L)^2$. They are in fact a non-trivial combination of different terms which in the limit $a/L\rightarrow 0$ are asymptotically $\propto (a/L)^2\ln (L/a)^{-\varGamma _i}$, or rather $\propto (a/L)^2 [{\bar{g}}^2(a^{-1})]^{\varGamma _i}$, where $\varGamma _i\in {\mathbb {R}}$, $i=1,2,\ldots ,$ and ${\bar{g}}^2(a^{-1})$ is the given renormalized coupling of the effective theory evaluated at a scale $\mu =a^{-1}$ (cf. Refs. [59–62]).

If terms of higher order than $a^2$ as well as logarithmic corrections to pure $a^2$ scaling were completely negligible in the data, the fit parameters $\sigma ^{(A)}_i$ and $\sigma ^{(B)}_i$ should perfectly agree. From the results in Fig. 5 we see that there is in fact agreement within one standard deviation. However, the difference between the results from the two fits is clearly systematic, with the results from fit B being always larger than those from fit A.

The issue becomes more evident if one tries to obtain a smooth parameterization for the continuum SSF from the fitted continuum values $\sigma _i$. As noticed earlier, a fit of $\varDelta \sigma _i=1/\sigma _i-1/u_i$ to a constant $\varDelta \sigma$ provides a good description of the continuum data $\sigma _i$ in the whole range of $u\in [2.1,6.5]$; this is the case for both $\varDelta \sigma ^{(A)}_i$ and $\varDelta \sigma ^{(B)}_i$ ($\chi ^2/\mathrm{dof}<1$). Given the 9 independent values of $\varDelta \sigma _i$ for each fit, the results for the corresponding $\varDelta \sigma$ are 3 times more precise than the individual $\varDelta \sigma _i$. The systematic effect then becomes clearly noticeable as one finds: $\varDelta \sigma ^{(A)}=-0.0823(4)$ and $\varDelta \sigma ^{(B)}=-0.0832(4)$, for the constant fits to $\varDelta \sigma ^{(A)}_i$ and $\varDelta \sigma ^{(B)}_i$, respectively.

The previous considerations show that the description of discretization errors as pure $(a/L)^2$ effects is in this case not accurate enough for the level of precision claimed in the continuum limit. Even though the different functional forms in Eqs. (13) and (14) fit the data well and perfectly agree with each other at finite *L*/*a*, the corresponding extrapolations for $a/L\rightarrow 0$ are clearly affected by some systematics. In Ref. [50], more conservative error estimates and robust central values for the continuum results are eventually obtained by carefully accounting as systematic uncertainties in the data the not entirely negligible effects of the higher-order terms neglected in Eqs. (13) and (14) (cf. Ref. [50] for the details).

The example above might not seem too pessimistic. However, it should come as a warning for the more general situation. Estimating properly the systematic uncertainties related to continuum limit extrapolations of high-precision data can easily become a challenge, particularly so if discretization errors are not small.

As recalled earlier, the leading asymptotic dependence of renormalized lattice quantities on the lattice spacing as $a\rightarrow 0$ is given by a combination of terms $\propto a^n [{\bar{g}}^2(a^{-1})]^{\varGamma _i}$, where the number and values of the $\varGamma _i$, as well as *n*, depend on the chosen discretization and set-up. The $\varGamma _i$ are in fact inferred from the anomalous dimensions of the fields defining the O($a^n$) counterterms in the SymEFT, and the order of perturbative improvement that has been possibly implemented (cf. Ref. [61]). Hence, when one considers a pure $a^{n}$ dependence for the discretization errors, one is implicitly assuming that all $\varGamma _i\approx 0$. This, however, cannot be taken for granted.

For most cases of interest, the leading discretization effects have $n=2$, i.e. they are of O($a^2$).^16^ The results of Refs. [61, 62] then show that in the case of QCD we have O(10) different terms that contribute in general, and $\varGamma _i\approx [-0.1,3]$ for several common discretizations and values of ${N_{\mathrm{f}}}=2-4$.^17^,^18^ Having all $\varGamma _i > rsim 0$ is certainly positive. In particular, the contributions relevant in the massless theory all have $\varGamma _i>0$, which implies a faster approach to the continuum limit with respect to pure $a^2$ terms. However, the large number of terms contributing makes for a complicated pattern of discretization errors in the general case, with no clear contribution(s) dominating. As a result, it may be difficult in practice to identify the terms that are actually relevant. Moreover, terms of the form $a^2[{\bar{g}}^2(a^{-1})]^{\varGamma _i}$ with $\varGamma _i\approx 2-3$ can be hard to distinguish from $a^3$ or $a^4$ terms in a limited range of lattice spacings when statistical uncertainties are present.^19^ The continuum estimates obtained by including different contributions, on the other hand, may vary appreciably. In this situation, precise and robust final estimates are not easily achieved.

We stress that it is particularly important to take these considerations into account when aiming for precise determinations of short-distance quantities like the couplings. As discussed in previous sections, in the most interesting region of high energy, $\mu \gg \varLambda$, $a\mu$ may not be so small. Continuum extrapolations are thus likely to be difficult and require special attention. Following the lines of Refs. [61, 62] one should take the non-trivial *a*-dependence predicted by SymEFT into account, provided the information is available. If this is not the case, one should try at least to estimate the uncertainties associated with neglecting logarithmic corrections to classical scaling, e.g. by considering terms $\propto a^2[{\bar{g}}^2(a^{-1})]^{\varGamma _i}$, with $\varGamma _i\approx 1-3$, in the fit ansätze. Ideally, one would like to be in the situation where within the uncertainties the continuum estimates do not sensibly depend on whether these terms are considered or not.

Given the observations above, we want to bring the reader’s attention to a recent study where the non-trivial *a*-dependence of discretization effects was found to be a relevant issue. Specifically, we consider the computations of Refs. [51, 63] of the GF-coupling in the pure Yang-Mills theory using Numerical Stochastic Perturbation Theory.^20^ In this framework, the lattice theory is numerically solved through a Monte Carlo simulation up to a finite order in the bare coupling $g_0$ [64, 65]. From expectation values in this “truncated theory” one can obtain the perturbative coefficients of the expansion of lattice quantities in $g_0$.

**Fig. 6:**
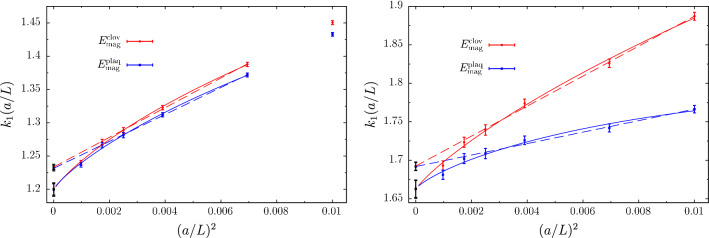
Continuum extrapolations for $k_1(a/L)$. Results for $c=0.3$ (left panel) and $c=0.4$ (right panel) are shown, for two different discretizations of the relevant observable. Different fits to the data are considered, cf. text

In Refs. [51, 63], the GF-coupling with SF boundary conditions ${\bar{g}}^2_{\mathrm{GF}}(\mu )$ has been computed up to two-loop order in $g_0^2$. Using the relation between $\alpha _0\equiv g_0^2/(4\pi )$ and $\alpha _{\overline{\mathrm{MS}}}\equiv \alpha ^{({N_{\mathrm{f}}}=0)}_{\overline{\mathrm{MS}}}$ [66, 67], one can thus infer the relation

15 $$\begin{aligned} \alpha _{\mathrm{GF}}(\mu )= & {} \alpha _{\overline{\mathrm{MS}}}(\mu ) + k_1(a/L)\alpha ^2_{\overline{\mathrm{MS}}}(\mu )\nonumber \\&\quad + k_2(a/L)\alpha ^3_{\overline{\mathrm{MS}}}(\mu )+\ldots . \end{aligned}$$

The coefficients $k_1(a/L)$, $k_2(a/L)$ are functions of the resolution *a*/*L* considered for the lattice. In order to obtain the matching relation between the couplings in the continuum limit the coefficients must be extrapolated for $a/L\rightarrow 0$. Focusing on the 1-loop coefficient, $k_1(a/L)$, from SymEFT we expect that (see Refs. [51, 63])

16 $$\begin{aligned} k_1(a/L)\overset{a/L\rightarrow 0}{\sim } k_1(0)+ \sum _{m=2}^\infty \sum _{n=0}^1 r_{mn} (a/L)^m \ln (L/a)^n\,, \end{aligned}$$

with $r_{mn}$ some constants. Note that the coefficient $r_{21}$ of the leading term $\propto \ln (L/a)$ implicitly depends on the $\varGamma _i$ predicted by SymEFT (cf. Sect. 5.2. of Ref. [61] and also Ref. [62]). Compared to the case of the full theory, the results from the truncated theory have a simpler (yet non-trivial) cutoff dependence. Given the high precision reached in these calculations, this allows for a clean illustration of the difficulties in continuum extrapolations.

In Fig. 6 we show the results from Ref. [63] for $k_1(a/L)$ for two different values of the parameter *c*, 0.3 and 0.4, that specifies the GF-scheme (cf. Refs. [51, 63] and Eq. (53)). Two different discretizations of the observable defining the coupling ($E^{\mathrm{clov}}_{\mathrm{mag}}$, $E^\mathrm{plaq}_{\mathrm{mag}}$) are also considered. The simulated lattices have sizes $L/a=10-32$.

Starting from the results for $c=0.3$ (left panel of Fig. 6), we see how the data is very precise but discretization effects are sizable. In the plot we then show two types of extrapolations to the continuum limit. For the first type (solid lines), lattices with $L/a=12-32$ are fitted to the asymptotic form, Eq. (16), considering the leading terms $m=2,n=0,1$. The fits are good, $\chi ^2/\mathrm{dof}\sim 1$, and the extrapolated results for the two discretizations agree well. The $m=2,n=1$, term is in fact crucial to obtain good fits. For the second set of extrapolations (dashed lines), we consider instead lattices with $L/a=12-24$. In this case the data can be very well described by a pure $(a/L)^2$ term ($m=2, n=0$) over the whole range of lattice sizes. The continuum extrapolated values obtained from these fits have significantly smaller statistical errors than the ones from the previous fits, and yet there is perfect agreement between the two discretizations. On the other hand, the results deviate from the previous estimates by several of their standard deviations.

The results for $c=0.4$ exhibit qualitatively the same features, although the statistical errors on $k_1(a/L)$ are about a factor 2 larger and the two discretizations now show rather different lattice artifacts. On the other hand, cutoff effects are generally smaller than for $c=0.3$, and we thus include $L/a=10$ in the fits. It is clear that in both cases, $c=0.3,0.4$, a reliable continuum extrapolation for $k_1(a/L)$ is challenging due to the non-trivial *a*-dependence of the data. In particular, larger lattices than the ones considered here are clearly needed in order to obtain accurate continuum results (cf. Ref. [51] for the final determination).

In conclusion, through these examples we saw how assessing the systematics related to the continuum limit extrapolations of couplings can be challenging. This is especially true when one wants to maintain the high precision reached on the lattice data also in the continuum limit, but discretization errors are large. It then becomes hard to avoid systematic biases in the final determinations. To this end, it is crucial to test all the assumptions that enter the functional forms chosen for the extrapolations. In particular, we must keep in mind that good fits do not necessarily mean good results for parameters, especially for extrapolations outside the range covered by the data.

## Heavy-quark decoupling

So far we focused on the main challenges that stand on the way of a precise determination of $\varLambda ^{({N_{\mathrm{f}}})}_{\overline{\mathrm{MS}}}$ and discussed in detail the cases of ${N_{\mathrm{f}}}=0,3$. The interesting quantity for phenomenology, however, is $\varLambda ^{(5)}_{\overline{\mathrm{MS}}}$. At present, lattice estimates of $\varLambda ^{(5)}_{\overline{\mathrm{MS}}}$ are for the most part based on determinations of $\varLambda ^{(3)}_{\overline{\mathrm{MS}}}$, while just a handful are obtained from $\varLambda ^{(4)}_{\overline{\mathrm{MS}}}$ (cf. Ref. [3]). As we shall recall in the next subsection, the most common strategy to obtain $\varLambda ^{(5)}_{\overline{\mathrm{MS}}}$ is in fact to non-perturbatively compute $\varLambda ^{(3)}_{\overline{\mathrm{MS}}}$ through simulations of the ${N_{\mathrm{f}}}=3$ theory and then rely on perturbative decoupling relations for the heavy quarks to estimate the ratios $\varLambda ^{(4)}_{\overline{\mathrm{MS}}}/ \varLambda ^{(3)}_{\overline{\mathrm{MS}}}$ and $\varLambda ^{(5)}_{\overline{\mathrm{MS}}}/ \varLambda ^{(4)}_{\overline{\mathrm{MS}}}$ (see e.g. Ref. [3]).

The main reason for this is because, as is well-known, simulating the charm quark dynamically is at present challenging, let alone the case of the bottom quark. While the inclusion of the charm quark in the computation of the running of the QCD coupling may be only moderately challenging with a suitable strategy (see e.g. Ref. [68]), it does pose important difficulties in large-volume hadronic simulations. Besides the increased computational cost in simulating an additional quark with respect to ${N_{\mathrm{f}}}=2+1$ simulations, and the more complicated tuning of the bare QCD parameters necessary to define proper lines of constant physics, discretization effects are a serious source of concern. Given the currently most accessible lattice spacings in hadronic simulations, say $a > rsim 0.05\,\mathrm{fm}$, we have that $am_c > rsim 0.3$ and $am_b > rsim 1$, where for definiteness we took $m_c\approx 1.27\,\mathrm{GeV}$ and $m_b\approx 4.2\,\mathrm{GeV}$. In the hadronic regime it is therefore a real challenge to control the discretization effects induced by including the charm quark in simulations, and unrealistic for the case of the bottom quark. This is particularly true for the case of Wilson quarks where the charm quark can potentially introduce large O($am_c$) effects, unless a complete Symanzik O(*a*) improvement programme is carried out, which is certainly no simple task (see e.g. Ref. [69]).

In this situation, it is mandatory to assess the reliability of the strategy presented above for the determination of $\varLambda ^{(5)}_{\overline{\mathrm{MS}}}$. To this end, in the following we shall recall the general theory of decoupling of heavy quarks and critically address its application in lattice determinations of $\varLambda ^{(5)}_{\overline{\mathrm{MS}}}$. This includes both the usage of perturbation theory for the inclusion of heavy-quark loops in the running of the QCD coupling, that is to estimate the ratios $\varLambda _{\overline{\mathrm{MS}}}^{(4)}/\varLambda _{\overline{\mathrm{MS}}}^{(3)}$, $\varLambda _{\overline{\mathrm{MS}}}^{(5)}/\varLambda _{\overline{\mathrm{MS}}}^{(4)}$, as well as the determination of the physical units of $\varLambda ^{(5)}_{\overline{\mathrm{MS}}}$ from scale setting in the ${N_{\mathrm{f}}}=2+1$ theory. As we shall see, given the current precision on $\varLambda ^{(3)}_{\overline{\mathrm{MS}}}$, accounting for heavy-quark effects by means of perturbation theory in the running of the QCD coupling is remarkably accurate, even for the case of the charm quark. In addition, charm-quark effects in (dimensionless) low-energy quantities are found to be quite small, supporting the fact that ${N_{\mathrm{f}}}=2+1$ QCD is accurate enough for establishing the physical scale. As a result, competitive determinations of $\varLambda ^{(5)}_{\overline{\mathrm{MS}}}$ are possible from results in the ${N_{\mathrm{f}}}=3$ flavor theory.

### The effective theory for heavy-quark decoupling and the QCD couplings

In this subsection we introduce the effective theory of heavy quarks and recall how this is conventionally applied in the determination of $\varLambda _{\overline{\mathrm{MS}}}^{(5)}$. We refer the reader to Refs. [70, 71] for a more detailed presentation.

#### The effective theory for heavy-quark decoupling

We begin by considering QCD with ${N_{\mathrm{f}}}$ flavors of quarks, which in short we denote $\mathrm{QCD}_{{N_{\mathrm{f}}}}$. Of these, ${N_{\ell }}$ are considered to be light, while the other ${N_{\mathrm{h}}}\equiv {N_{\mathrm{f}}}-{N_{\ell }}$ are heavy. For simplicity, we assume that the light quarks are degenerate with mass *m*, while the heavy quarks are also degenerate but with a mass $M\gg \varLambda$. The effective theory associated with the decoupling of the heavy quarks is formally obtained by integrating out in the functional integral the fields associated with the heavy quarks [72]. The field theory that results is characterized by having an infinite number of non-renormalizable interactions, which are suppressed at low energies by negative powers of the heavy-quark masses *M*. The couplings of the effective theory can be fixed order by order in $M^{-1}$ by requiring that, at each given order, a finite number of observables is equal to the corresponding ones in the fundamental theory. Once the couplings are fixed up to a certain order $M^{-n}$, the effective theory is said to be matched to the fundamental one at this order, and can be used to describe the effects of the heavy quarks at low energies up to corrections of O($M^{-n-1}$). In this sense, we say that as $M\rightarrow \infty$ the heavy quarks decouple from low-energy physics as their effects eventually fade away [73].

In formulas, the Lagrangian of the effective theory is of the general form (see e.g. Ref. [71])

17 $$\begin{aligned} {\mathcal {L}}_{\mathrm{dec}}={\mathcal {L}}_0 + {1\over M}{\mathcal {L}}_1 + {1\over M^2}{\mathcal {L}}_2+\ldots \,, \end{aligned}$$

where the leading order corresponds to the Lagrangian of $\mathrm{QCD}$ with ${N_{\ell }}$ light quarks, i.e. ${\mathcal {L}}_0={\mathcal {L}}_\mathrm{QCD_{N_{\ell }}}$, while the corrections ${\mathcal {L}}_k$, $k\ge 1$, consist of linear combinations of local fields of mass dimension $4+k$, i.e.

18 $$\begin{aligned} {\mathcal {L}}_k= \sum _i \omega ^{(k)}_i \varPhi ^{(k)}_i\,, \qquad [\varPhi ^{(k)}_i]=4+k\,, \end{aligned}$$

with $\omega ^{(k)}_i$ dimensionless couplings. The fields $\varPhi ^{(k)}_i$ are built from the light-quark and gluon fields, and include possible powers of the light-quark masses. They must respect the symmetries of the fundamental $\mathrm{QCD}_{{N_{\mathrm{f}}}}$ theory, as in particular gauge invariance, Euclidean (or Lorentz) symmetry, and chiral symmetry.

In the case where the light quarks are massless, the leading-order effective theory, $\mathrm{QCD}_{N_{\ell }}$, has a single parameter: the gauge coupling ${\bar{g}}^{({N_{\ell }})}(\mu )$. The effective and fundamental theory are therefore matched at leading order in $M^{-1}$ once ${\bar{g}}^{({N_{\ell }})}(\mu )$ is matched. This requires that ${\bar{g}}^{({N_{\ell }})}(\mu )$ is properly prescribed at a given renormalization scale in a given renormalization scheme in terms of the coupling ${\bar{g}}^{({N_{\mathrm{f}}})}(\mu )$ of the fundamental theory and the heavy-quark masses *M*.^21^ In addition, provided that the fundamental theory is defined on a manifold without boundaries,^22^ it is possible to show that ${\mathcal {L}}_1=0$ and therefore O($M^{-1}$) corrections are absent [71].^23^ In this situation, the leading-order corrections induced by the heavy quarks are suppressed as $M^{-2}$ at low energy.

In the case where the light quarks have a non-vanishing mass, the only mass-dimension 5 fields allowed in ${\mathcal {L}}_1$ are given by the fields of the leading-order Lagrangian ${\mathcal {L}}_\mathrm{QCD_{N_{\ell }}}$ multiplied by the light-quark masses *m* [71]. Their effect can be reabsorbed into a redefinition of the gauge coupling and light-quark masses of O(*m*/*M*). Of course, in the case of massive light quarks the matching of the effective and fundamental theory at leading order also requires the matching of the light-quark masses. In addition, the couplings $\omega _i^{(k)}$ of the corrections ${\mathcal {L}}_{k\ge 2}$ will depend in general on the light-quark masses, too.

#### The effective theories and their couplings

The application of the effective theory for heavy quarks in the determination of the QCD couplings was first advocated by Weinberg in his seminal paper on effective field theories [72]. The idea is based on the observation that for mass-independent renormalization schemes the RG equations of the renormalizable couplings of the effective theory completely decouple from the others. This means that the couplings of the non-renormalizable interactions can be completely ignored when determining the variation of the running coupling ${\bar{g}}^{({N_{\ell }})}(\mu )$ of the effective theory with the energy scale $\mu$. The heavy quarks affect the value of the coupling of the effective theory only through the matching with the coupling of the fundamental theory, ${\bar{g}}^{({N_{\mathrm{f}}})}(\mu )$.

The matching relation between ${\bar{g}}^{({N_{\ell }})}(\mu )$ and ${\bar{g}}^{({N_{\mathrm{f}}})}(\mu )$ can in principle be established in perturbation theory. This is best done at a scale $\mu _{\mathrm{match}} \approx M$ [23, 72]. Assuming the validity of perturbation theory at this scale, if the running coupling ${\bar{g}}^{({N_{\ell }})}(\mu _{\mathrm{match}})$ in the effective theory is known, one can turn tables and obtain ${\bar{g}}^{({N_{\mathrm{f}}})}(\mu _\mathrm{match})$ from inverting the matching conditions.

In phenomenological applications of this strategy (see e.g. Ref. [5]), the value of ${\bar{g}}^{({N_{\ell }})}(\mu _{\mathrm{match}})$ is extracted from the value of the coupling ${\bar{g}}^{({N_{\ell }})}(\mu _{\mathrm{low}})$ at some lower energy scale, $\mu _{\mathrm{low}}\ll M$. The latter is obtained by comparing the perturbative expansion for some process ${{\mathcal {O}}}(q)$ with characteristic energy scale $q\approx \mu _{\mathrm{low}}$, with its experimental results. The effects of the heavy quarks in ${{\mathcal {O}}}(q)$ are expected to be suppressed as O($(q/M)^2$) (cf. Sect. 3.1.1). Hence, assuming that these effects can be neglected, the perturbative expansion of ${{\mathcal {O}}}(q)$ can be considered in the ${N_{\ell }}$-flavor theory, which allows the coupling ${\bar{g}}^{({N_{\ell }})}(\mu _{\mathrm{low}})$ to be extracted. As observed above, the determination of the running of the effective coupling in the ${N_{\ell }}$-flavor theory does not require any input from the fundamental theory: one can thus readily obtain ${\bar{g}}^{({N_{\ell }})}(\mu _\mathrm{match})$ from ${\bar{g}}^{({N_{\ell }})}(\mu _{\mathrm{low}})$. Clearly, for this strategy to work in practice the energy scale $\mu _{\mathrm{low}}\ll M$ must be yet sufficiently high for perturbation theory to apply.

In non-perturbative applications on the lattice, the strategy presented above is realized in the following way (see e.g. Ref. [3]). Firstly, as discussed in Sect. 2.2.3, through the study of the non-perturbative running of a given massless coupling within the effective ${N_{\ell }}$-flavor theory, one determines the ratio $\varLambda _{\overline{\mathrm{MS}}}^{({N_{\ell }})}/\mu _{\mathrm{had}}$, where $\mu _\mathrm{had}$ is a convenient (not necessarily physical) low-energy scale. Assuming that the effects of the heavy quarks can be neglected in the ratio of low-energy scales $\mu _{\mathrm{had}}/\mu _{\mathrm{phys}}$, where $\mu _{\mathrm{phys}}$ is an experimentally accessible hadronic quantity, this can also be computed within ${N_{\ell }}$-flavor QCD. The physical units of $\mu _{\mathrm{had}}$ and therefore $\varLambda _{\overline{\mathrm{MS}}}^{({N_{\ell }})}$ can then be established by taking $\mu _{\mathrm{phys}}$ from experiments. As a second step, as we shall see in detail in the following subsection, the matching relation between the couplings of the effective and fundamental theory is expressed as a relation between their $\varLambda$-parameters. The ratio $\varLambda _{\overline{\mathrm{MS}}}^{({N_{\mathrm{f}}})}/ \varLambda ^{({N_{\ell }})}_{\overline{\mathrm{MS}}}$ is thus estimated using perturbation theory at a scale $\mu \approx M$. From this estimate and the results for $\varLambda _{\overline{\mathrm{MS}}}^{({N_{\ell }})}$, $\varLambda _{\overline{\mathrm{MS}}}^{({N_{\mathrm{f}}})}$ is obtained.

It is important to note that in extracting $\varLambda _{\overline{\mathrm{MS}}}^{({N_{\ell }})}$ from the running of the chosen non-perturbative scheme, perturbation theory can be applied at arbitrarily large energy scales. The perturbative matching between the effective and fundamental theory, instead, is best performed at a scale $\mu \approx M$, where *M* is set by the mass of the given heavy quarks that decouple. Whether perturbation theory is accurate in this step hence depends on how heavy these quarks are. As we shall see, in the $\overline{\mathrm{MS}}$-scheme the perturbative matching is remarkably accurate already for masses *M* close to that of the charm quark.

In the next subsection we describe in detail the perturbative matching of the QCD couplings. We follow the lines of the presentation in Ref. [71], and start by reformulating this matching in terms of $\varLambda$-parameters [23]. After doing so, we investigate the accuracy of a perturbative matching within perturbation theory itself, and later present a discussion on the typical size of non-perturbative corrections one can expect in these relations.

### Perturbative decoupling

#### Definitions

As we are interested only in the QCD coupling we assume for simplicity that the relevant effective theory is given by massless $\mathrm{QCD}_{{N_{\ell }}}$. As discussed above, at leading order in $M^{-1}$ the only parameter of the effective theory that needs to be fixed is therefore the running coupling ${\bar{g}}^{({N_{\ell }})}(\mu )$. The effective theory hence predicts any observable once the coupling in the chosen scheme is specified at some scale.

The general form of the relation between the couplings of the leading-order effective theory ${\bar{g}}^{({N_{\ell }})}$ and of the fundamental theory ${\bar{g}}^{({N_{\mathrm{f}}})}$, reads [23, 24, 72]

19 $$\begin{aligned} {[}{\bar{g}}^{({N_{\ell }})}(\mu /\varLambda ^{({N_{\ell }})})]^2= F_{{\mathcal {O}}}\big ([{\bar{g}}^{({N_{\mathrm{f}}})}(\mu /\varLambda ^{({N_{\mathrm{f}}})})]^2,M/\mu \big )\,, \end{aligned}$$

where for later convenience we explicitly wrote the dependence of the couplings on their corresponding $\varLambda$-parameter. The function $F_{{\mathcal {O}}}$ depends in principle on the specific observable ${{\mathcal {O}}}$ that is used to establish the matching between the two theories. The dependence on the observable, however, is suppressed by powers of $M^{-1}$. In perturbation theory, these power corrections can be uniquely isolated from the logarithmic terms in *M* and can therefore be dropped. This is consistent with matching the theories at leading order in $M^{-1}$. In doing so, the relation between the couplings becomes universal, i.e independent on the specific matching condition. It only depends on the renormalization schemes chosen for the couplings.

In the $\overline{\mathrm{MS}}$-scheme the matching relation (also referred to as decoupling relation) is known up to 4-loop order. Below, we consider this only for the convenient choice of matching scale $\mu =m_*$, where $m_*$ is implicitly defined by the equation ${\overline{m}}_{\overline{\mathrm{MS}}}(m_*)=m_*$, where ${\overline{m}}_{\overline{\mathrm{MS}}}(\mu )\equiv {\overline{m}}^{({N_{\mathrm{f}}})}_{\overline{\mathrm{MS}}} (\mu /\varLambda _{\overline{\mathrm{MS}}}^{({N_{\mathrm{f}}})})$ are the running masses of the heavy quarks in the fundamental theory in the $\overline{\mathrm{MS}}$-scheme. Given this choice, the matching relation reads:

20 $$\begin{aligned}&\big [{\bar{g}}^{({N_{\ell }})}_{\overline{\mathrm{MS}}}(m_*/\varLambda ^{({N_{\ell }})}_{\overline{\mathrm{MS}}})\big ]^2\!=\! g_*^2 C(g_*),\, \quad g_*\!\equiv \! {\bar{g}}^{({N_{\mathrm{f}}})}_{\overline{\mathrm{MS}}}(m_*/\varLambda ^{({N_{\mathrm{f}}})}_{\overline{\mathrm{MS}}})\,,\nonumber \\&C(g_*)=1+h_2 g_*^4 + h_3 g_*^6 + h_4 g_*^8 + \ldots \,, \end{aligned}$$

where the $h_i$ are pure numbers that depend on both ${N_{\mathrm{h}}}$ and ${N_{\ell }}$ [74–78]. As explained in these references, the particular choice of matching scale makes all contributions $\propto \log ({\overline{m}}_{\overline{\mathrm{MS}}}(\mu )/\mu )$ appearing in the matching relation vanish, and it is considered to be optimal. This implies in particular that the $g_*^2$ term in $C(g_*)$ is absent. In the general case, the scales ${\overline{m}}_{\overline{\mathrm{MS}}}(\mu )$ and $\mu$ should anyway not be chosen too separated. Large coefficients otherwise appear in the perturbative matching relation which can compromise its applicability.

As anticipated, from the perspective of lattice applications it is compelling to recast the matching relation, Eq. (20), in terms of RG-invariant (RGI) quantities. Specifically, this means the $\varLambda$-parameter of the effective theory, $\varLambda ^{({N_{\ell }})}_{\overline{\mathrm{MS}}}$, that of the fundamental theory $\varLambda ^{({N_{\mathrm{f}}})}_{\overline{\mathrm{MS}}}$, and the RGI quark-mass *M* of the heavy quarks. The latter can be defined as

21 $$\begin{aligned} M&\equiv {\overline{m}}^{({N_{\mathrm{f}}})}_{\overline{\mathrm{MS}}}(\mu ) \varphi ^{({N_{\mathrm{f}}})}_{\mathrm{m, \overline{\mathrm{MS}}}}({\bar{g}}^{({N_{\mathrm{f}}})}_{\overline{\mathrm{MS}}}(\mu ))\,,\nonumber \\ \varphi ^{({N_{\mathrm{f}}})}_{\mathrm{m,\overline{\mathrm{MS}}}}({\bar{g}})&\equiv (2b_0({N_{\mathrm{f}}}){\bar{g}}^2)^{-{d_0\over 2b_0({N_{\mathrm{f}}})}}\times \nonumber \\&\times \exp \bigg \{-\int _0^{{\bar{g}}}\mathrm{d}g \bigg [{\tau _{\overline{\mathrm{MS}}}^{({N_{\mathrm{f}}})}({g})\over \beta _{\overline{\mathrm{MS}}}^{({N_{\mathrm{f}}})}({g})} - {d_0\over b_0({N_{\mathrm{f}}})g} \bigg \}\,, \end{aligned}$$

where $b_0({N_{\mathrm{f}}})$ is the 1-loop coefficient of the $\beta$-function, Eq. (7), and the function

22 $$\begin{aligned} \tau ^{({N_{\mathrm{f}}})}_{\overline{\mathrm{MS}}}({\bar{g}})\equiv {\mu \over {\overline{m}}^{({N_{\mathrm{f}}})}_{\overline{\mathrm{MS}}}(\mu )} {\mathrm{d}{\overline{m}}^{({N_{\mathrm{f}}})}_{\overline{\mathrm{MS}}}(\mu )\over \mathrm{d}\mu } \bigg |_{{\bar{g}}} \end{aligned}$$

encodes the scale-dependence of the quark masses in the ${N_{\mathrm{f}}}$-flavor theory (in the $\overline{\mathrm{MS}}$-scheme). It has a perturbative expansion

23 $$\begin{aligned} \tau ^{({N_{\mathrm{f}}})}_{\overline{\mathrm{MS}}}({\bar{g}})\overset{{\bar{g}}\rightarrow 0}{\approx } -{\bar{g}}^2\{d_0+{\bar{g}}^2 d_1+\ldots \}\,, \quad d_0=8/(4\pi )^2\,,\qquad \end{aligned}$$

known up to 5-loops [79–82], where the actual scheme and ${N_{\mathrm{f}}}$-dependence start from $d_i$, $i\ge 1$. Note that even though in Eq. (21) we conveniently defined the RGI mass *M* through the $\overline{\mathrm{MS}}$-scheme, its value is in fact scheme independent, as long as mass-independent schemes are considered for the quark masses. This means in particular that *M* can be non-perturbatively defined through any non-perturbative (massless) renormalization scheme.

Using the above definitions, together with the definitions in Eq. (4), and the matching relation Eq. (20), it is immediate to conclude that [70, 71]

24 $$\begin{aligned} P_{\ell ,\mathrm f}(M/\varLambda _{\overline{\mathrm{MS}}}^{({N_{\mathrm{f}}})})\equiv {\varLambda _{\overline{\mathrm{MS}}}^{({N_{\ell }})}\over \varLambda _{\overline{\mathrm{MS}}}^{({N_{\mathrm{f}}})}}\Bigg |_{\mathrm{matched}}= {\varphi _{\mathrm{g,\overline{\mathrm{MS}}}}^{({N_{\ell }})}(g_*\sqrt{C(g_*)}) \over \varphi _{\mathrm{g,\overline{\mathrm{MS}}}}^{({N_{\mathrm{f}}})}(g_*)}\,.\nonumber \\ \end{aligned}$$

As anticipated by our notation, $P_{\ell ,\mathrm f}$ can be considered as a function of $M/\varLambda _{\overline{\mathrm{MS}}}^{({N_{\mathrm{f}}})}$, since the value of the coupling $g_*$ can be expressed in terms of the RGI parameters through the relation

25 $$\begin{aligned} {M\over \varLambda _{\overline{\mathrm{MS}}}^{({N_{\mathrm{f}}})}}= {\varphi _{\mathrm{m,\overline{\mathrm{MS}}}}^{({N_{\mathrm{f}}})}(g_*) \over \varphi _\mathrm{g,\overline{\mathrm{MS}}}^{({N_{\mathrm{f}}})}(g_*)}\,, \qquad g_*={\bar{g}}^{({N_{\mathrm{f}}})}_{\overline{\mathrm{MS}}}( {M/\varLambda _{\overline{\mathrm{MS}}}^{({N_{\mathrm{f}}})}}). \end{aligned}$$

In the limit where $M/\varLambda ^{({N_{\mathrm{f}}})}_{\overline{\mathrm{MS}}}\rightarrow \infty$, the coupling $g_*$ goes to zero and the function $P_{\ell ,\mathrm f}$ admits an asymptotic perturbative expansion in terms of $g_*^2$.

#### Perturbative uncertainties

We now want to study the behavior of the perturbative expansion of $P_{\ell ,\mathrm f}(M/\varLambda )$. We shall focus our attention on the functions $P_{\mathrm{3,4}}$ and $P_{\mathrm{4,5}}$, which are the relevant ones for estimating $\varLambda ^{(5)}_{\overline{\mathrm{MS}}}$ given $\varLambda ^{(3)}_{\overline{\mathrm{MS}}}$.

**Fig. 7:**
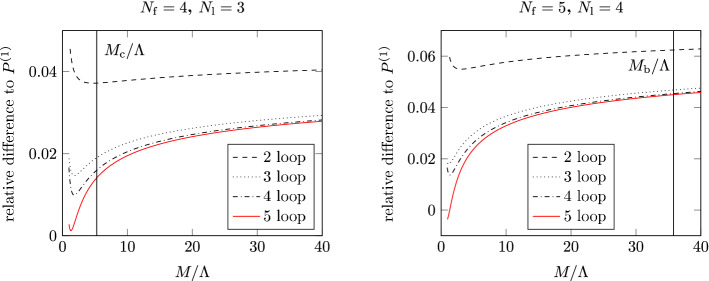
Relative differences from the “1-loop approximation” $P^{(1)}_{\ell , \mathrm f}(M/\varLambda )=(M/\varLambda )^{\eta _0}$, $\eta _0=2{N_{\mathrm{h}}}/(33-2{N_{\ell }})$, for different orders of the perturbative expansion of $P_{\ell , \mathrm f}(M/\varLambda )$ as a function of $M/\varLambda$ [71]. The results for ${N_{\ell }}=3$, ${N_{\mathrm{f}}}=4$ (${N_{\ell }}=4$, ${N_{\mathrm{f}}}=5$) are given in the left (right) panel. The values for the RGI charm ($M_c$) and bottom ($M_b$) quark masses in units of the proper $\varLambda$-parameters are marked by vertical lines

In Fig. 7 we present the results taken from Ref. [71] for the relative deviation

26 $$\begin{aligned} {(P_{\ell ,\mathrm f}-P_{\ell ,\mathrm f}^{(1)})\over P^{(1)}_{\ell ,\mathrm f}} \end{aligned}$$

of $P_{\mathrm{3,4}}$ (left plot) and $P_{\mathrm{4,5}}$ (right plot), from their *unsystematic* 1-loop approximation $P^{(1)}_{\ell , \mathrm f}(M/\varLambda )=(M/\varLambda )^{\eta _0}$, where $\eta _0=2{N_{\mathrm{h}}}/(33-2{N_{\ell }})$ [71].^24^ The results are shown as a function of $M/\varLambda$ and for different orders of perturbation theory. The order in perturbation theory refers to the order at which the $\beta$-functions entering Eqs. (24)–(25) are considered. The $\tau$-function, Eq. (23), as well as the matching function $C(g_*)$ of Eq. (20) are considered to a consistent order in the expansion (see Ref. [71] for the details). The values of the RGI masses of the charm and bottom quarks ($M_c$ and $M_b$, respectively) in units of the relevant $\varLambda$-parameter are also shown. These are inferred from the results of the PDG [5].

Starting from the case of $P_{4,5}$, we see how the difference between the 3-loop and 2-loop results is around 2% at the *b*-quark mass. The 4- and 5-loop results then differ only by very tiny corrections from the 3-loop approximation. Looking at the behavior of the perturbative series alone, the perturbative prediction for the decoupling of the *b*-quark appears to be very reliable and accurate. Similar conclusions can be drawn for the decoupling of the charm quark. Also in the case of $P_{3,4}$, the difference between the 3- and 2-loop results is about 2% at the charm-quark mass, and higher-order corrections are all much smaller.

In conclusion, the perturbative results suggest that a perturbative treatment of the matching between the relevant effective and fundamental theories introduces only errors at the sub-percent level in the functions $P_{\ell ,\mathrm f}$, and therefore in the connection of their $\varLambda$-parameters.

### Non-perturbative decoupling

#### Definitions

Judging from perturbation theory alone, the perturbative description of the decoupling of heavy quarks seems to work remarkably well. The rapid convergence of the different orders of the expansion of $P_{\ell ,\mathrm f}$ at the values of both the charm- and bottom-quark masses, seems to suggest that the series is well within its regime of applicability and higher-order corrections are small. On the other hand, the perturbative expansion cannot tell us anything about the size of non-perturbative corrections to the decoupling relations and whether perturbation theory actually applies at all. Whether non-perturbative effects are significant within the target precision at the relevant quark masses can only be established through a non-perturbative investigation.

In order to set the grounds for estimating the error that one makes when using a perturbative approximation for $P_{\ell ,\mathrm f}$ to extract $\varLambda _{\overline{\mathrm{MS}}}^{({N_{\mathrm{f}}})}$ from $\varLambda _{\overline{\mathrm{MS}}}^{({N_{\ell }})}$, let us begin by recasting the matching of the effective and fundamental theory in more non-perturbative terms.^25^

The leading-order effective theory describes the fundamental theory at low energy only when the corresponding $\varLambda$-parameter $\varLambda ^{({N_{\ell }})}$ is a properly chosen function of the scale $\varLambda ^{({N_{\mathrm{f}}})}$ of the fundamental theory and of the RGI mass *M* of the heavy quarks. To be more precise, consider a low-energy mass-scale ${\mathcal {S}}\ll M$. This could be, for instance, a hadronic mass or any of the popular technical scales, $t^{-1/2}_0,r^{-1}_0,w^{-1}_0$ [47, 52, 83]. Matching then means fixing the scale $\varLambda ^{({N_{\ell }})}$ through the condition [71]

27 $$\begin{aligned} {\varLambda ^{({N_{\ell }})}\over {\mathcal {S}}^{({N_{\ell }})}}= P_{\ell ,\mathrm f}^{{\mathcal {S}}}(M/\varLambda ^{({N_{\mathrm{f}}})}) {\varLambda ^{({N_{\mathrm{f}}})}\over {\mathcal {S}}^{({N_{\mathrm{f}}})}(M)}\,. \end{aligned}$$

In these equations ${\mathcal {S}}^{({N_{\ell }})}$ and ${\mathcal {S}}^{({N_{\mathrm{f}}})}(M)$ refer to the given low-energy scale computed in the effective and fundamental theory, respectively. Note in particular that the matching function $P_{\ell ,\mathrm f}^{{\mathcal {S}}}(M/\varLambda ^{({N_{\mathrm{f}}})})$ depends on the scale ${\mathcal {S}}$ that is considered in the matching relation. We also stress again that while the value of $\varLambda ^{({N_{\mathrm{f}}})}$ does not depend on *M* the one of $\varLambda ^{({N_{\ell }})}$ does depend through the matching condition.

Once $\varLambda ^{({N_{\ell }})}$ is properly fixed through Eq. (27) in terms of ${\mathcal {S}}$, for any other low-energy quantity ${\mathcal {S}}'$ we expect that

28 $$\begin{aligned} {\mathcal {S}}'^{({N_{\ell }})}={\mathcal {S}}'^{({N_{\mathrm{f}}})}(M) +\mathrm{O}\big (\big ({\varLambda ^{({N_{\mathrm{f}}})}/ M}\big )^2\big )\,. \end{aligned}$$

Note that ratios of low-energy scales, instead, do not depend on the value of the $\varLambda$-parameters and are therefore insensitive to their matching. For these, it readily holds that

29 $$\begin{aligned} {{\mathcal {S}}^{({N_{\ell }})}\over {\mathcal {S}}'^{({N_{\ell }})}}= {{\mathcal {S}}^{({N_{\mathrm{f}}})}(M)\over {\mathcal {S}}'^{({N_{\mathrm{f}}})}(M)} +\mathrm{O}\big (\big ({\varLambda ^{({N_{\mathrm{f}}})}/ M}\big )^2\big )\,, \end{aligned}$$

with ${\mathcal {S}}$ and ${\mathcal {S}}'$ any two low-energy scales. Given this observation, from Eq. (27) we conclude that

30 $$\begin{aligned} P_{\ell ,\mathrm f}^{{\mathcal {S}}}= P_{\ell ,\mathrm f}^{{\mathcal {S}}'}+\mathrm{O}(({\varLambda ^{({N_{\mathrm{f}}})}/ M})^2)\,. \end{aligned}$$

In other words, at the non-perturbative level the function $P_{\ell ,\mathrm f}^{{\mathcal {S}}}$ intrinsically comes with O($M^{-2}$) ambiguities. For this reason we shall often simply write it as $P_{\ell ,\mathrm f}$, keeping the dependence on ${\mathcal {S}}$ and the related ambiguities understood.

An interesting consequence of the above relations follows from multiplying Eq. (27) by ${\mathcal {S}}^{({N_{\mathrm{f}}})}(0)/\varLambda ^{({N_{\mathrm{f}}})}$, where ${\mathcal {S}}^{({N_{\mathrm{f}}})}(0)$ stands for the low-energy quantity ${\mathcal {S}}$ computed in the chiral limit of the ${N_{\mathrm{f}}}$-flavor theory. Through this simple manipulation we find that [70, 71]

31 $$\begin{aligned} {{\mathcal {S}}^{({N_{\mathrm{f}}})}(M)\over {\mathcal {S}}^{({N_{\mathrm{f}}})}(0)}&= Q^{{\mathcal {S}}}_{\ell ,\mathrm f} \times P^{{\mathcal {S}}}_{\ell ,\mathrm f}(M/\varLambda ^{({N_{\mathrm{f}}})}) \nonumber \\&= Q^{{\mathcal {S}}}_{\ell ,\mathrm f} \times P_{\ell ,\mathrm f}(M/\varLambda ^{({N_{\mathrm{f}}})}) +\mathrm{O}\big (\big ({\varLambda ^{({N_{\mathrm{f}}})}/ M}\big )^2\big )\,, \end{aligned}$$

where the factor

32 $$\begin{aligned} Q^{{\mathcal {S}}}_{\ell ,\mathrm f}\equiv {{\mathcal {S}}^{({N_{\ell }})}/\varLambda ^{({N_{\ell }})}\over {\mathcal {S}}^{({N_{\mathrm{f}}})}(0)/\varLambda ^{({N_{\mathrm{f}}})} }\,, \end{aligned}$$

is defined in terms of the massless ${N_{\mathrm{f}}}$- and ${N_{\ell }}$-flavor theories. The interesting aspect of Eq. (31) is that the ratio on the l.h.s. can be computed within the fundamental theory, while the r.h.s. is a consequence of the decoupling of the heavy quarks. In particular, we see how the mass dependence of the ratio ${{\mathcal {S}}^{({N_{\mathrm{f}}})}(M)/{\mathcal {S}}^{({N_{\mathrm{f}}})}(0)}$ is expressed in terms of the function $P^{{\mathcal {S}}}_{\ell , \mathrm f}$, while the factor $Q^{{\mathcal {S}}}_{\ell , \mathrm f}$ is just an overall constant. In the limit of large mass *M*, the mass dependence of this ratio is therefore expected to be universal and described by perturbation theory. As a result, this relation allows us to put at test the perturbative expansion of $P_{\ell , \mathrm f}$ and estimate the typical size of non-perturbative corrections.

To this end, it is convenient in practice to introduce the mass-scaling function [71]

33 $$\begin{aligned} \eta _{\ell ,\mathrm f}^{M,{\mathcal {S}}}(M) \equiv {M\over P^{{\mathcal {S}}}_{\ell ,\mathrm f}} {\partial P^{{\mathcal {S}}}_{\ell ,\mathrm f}\over \partial M}\bigg |_{\varLambda ^{({N_{\mathrm{f}}})}}\,. \end{aligned}$$

Considering Eq. (31), this can be computed from the mass dependence of any hadronic quantity as^26^

34 $$\begin{aligned} \eta _{\ell ,\mathrm f}^{M,{\mathcal {S}}}(M) = {M\over {\mathcal {S}}^{({N_{\mathrm{f}}})}(M)} {\partial {{\mathcal {S}}}^{({N_{\mathrm{f}}})}(M)\over \partial M}\bigg |_{\varLambda ^{({N_{\mathrm{f}}})}}\,, \end{aligned}$$

with no need for determining ${\mathcal {S}}^{({N_{\mathrm{f}}})}(0)$ or $Q_{\ell ,\mathrm f}$. As for the functions $P^{{\mathcal {S}}}_{\ell ,\mathrm f}$, the $\eta _{\ell ,\mathrm f}^{M,{\mathcal {S}}}$ obtained from different low-energy quantities ${\mathcal {S}}$ are expected to differ by O($(\varLambda ^{({N_{\mathrm{f}}})}/M)^2$) contributions. In the limit $M/\varLambda ^{({N_{\mathrm{f}}})}\rightarrow \infty$, however, the mass-scaling function becomes universal and can asymptotically be estimated in perturbation theory [71]. In the following we shall see how by studying the mass-scaling function we will be able to obtain valuable insight on the applicability of perturbation theory in computing $P_{\ell ,\mathrm f}$.

#### Non-perturbative corrections to decoupling

Non-perturbative effects in the decoupling of heavy quarks have been systematically investigated in a series of recent papers [70, 71, 84–87], which focus on the relevant case of the charm. The main question that these works contribute to answer is how good of an approximation ${N_{\mathrm{f}}}=2+1$ QCD is to the ${N_{\mathrm{f}}}=2+1+1$ flavor theory. From our perspective, we are particularly interested in understanding how precisely we can expect to obtain $\varLambda _{\overline{\mathrm{MS}}}^{(4)}$ (and consequently $\varLambda _{\overline{\mathrm{MS}}}^{(5)}$) from results in ${N_{\mathrm{f}}}=2+1$ QCD. As already mentioned, the first issue is to understand how accurately we can estimate $\varLambda _{\overline{\mathrm{MS}}}^{(4)}$ from $\varLambda _{\overline{\mathrm{MS}}}^{(3)}$ by relying on a perturbative approximation for $P_{3,4}(M_c/\varLambda _{\overline{\mathrm{MS}}}^{(4)})$. Secondly, we must question how much we can rely on setting the physical units of the theory using ${N_{\mathrm{f}}}=2+1$ QCD.

Studying the decoupling of the charm quark through simulations of ${N_{\mathrm{f}}}=2+1+1$ and ${N_{\mathrm{f}}}=2+1$ QCD is very challenging from the computational point of view. As a result, the physical effects one is after can easily end up being masked by the final uncertainties. For this reason, the authors of Refs. [70, 71, 84] have investigated non-perturbatively a model system given by QCD with ${N_{\mathrm{f}}}=2$ degenerate heavy quarks. When the mass of the doublet of quarks becomes large the heavy quarks eventually decouple, and the theory is expected to be described at low energy by the pure Yang-Mills theory, i.e. ${N_{\ell }}=0$ flavor QCD. Studying this model rather than the realistic case, avoids the complications of simulating light quarks. This allows one to reach much finer lattice spacings than typically possible in large-volume simulations with light quarks, because the volumes are similar to those in pure-gauge theory. Fine lattice spacings are essential to have discretization errors under control in the presence of quarks with masses close to that of the charm.

By studying this model one expects to be able to reliably estimate the typical size of the effects induced by the charm in low-energy physics. In particular, the absence of the light quarks is not expected to change the picture very much and their effect is likely more than compensated by the additional heavy quark present in the model. In fact, as we shall report below, some first results have been recently obtained for the more realistic situation where a single charm quark decouples in the presence of 3 mass-degenerate lighter quarks [87]. The results collected so far confirm the findings of the model study.

**Fig. 8:**
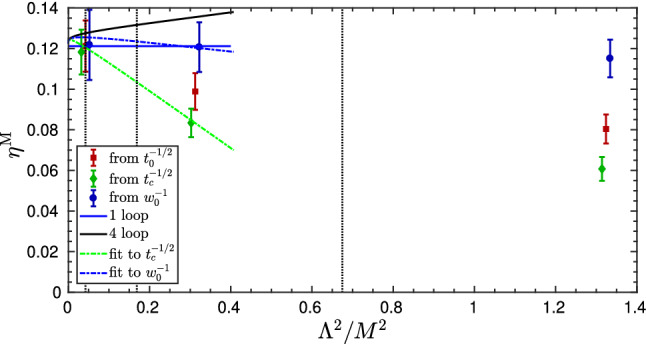
Mass dependence of the mass-scaling functions $\eta ^{M,{\mathcal {S}}}_{0,2}$ obtained from the hadronic scales ${\mathcal {S}}=t_0^{-1/2},t_c^{-1/2},w_0^{-1}$ [71]. The data for a given mass *M* are slightly displaced horizontally for better clarity. The non-perturbative results are compared to the perturbative estimates at 1- and 4-loop order. The dash-dotted lines are fits to the data for ${\mathcal {S}}=t_c^{-1/2}$ and $w_0^{-1}$ used to estimate the size of the non-perturbative corrections to $\eta _{0,2}^{M,{\mathcal {S}}}$ [71]. The vertical dotted lines mark the values of the quark mass $M_c,M_c/2$, and $M_c/4$

**Fig. 9:**
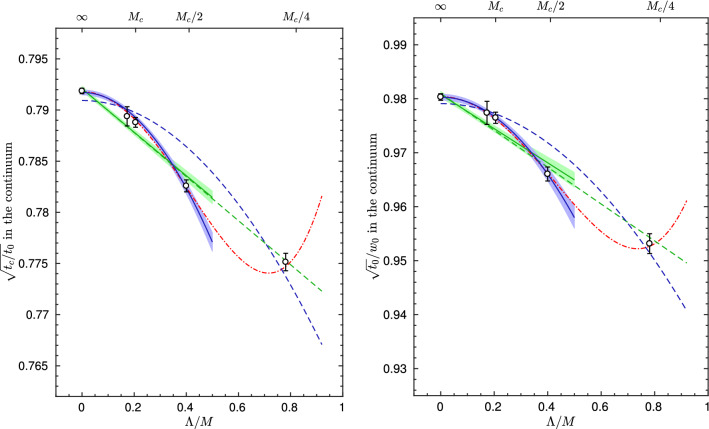
Continuum extrapolated values for $\sqrt{t_c/t_0}$ (left) and $\sqrt{t_0}/w_0$ (right) as a function of $\varLambda /M$ [84]. The line in the blue band is the leading-order effective theory prediction: $R(M) =R(\infty ) +k_R\times \varLambda ^2/M^2$, $R=\sqrt{t_c/t_0}, \sqrt{t_0}/w_0$, with $k_R$ a free parameter, fitted through points from $M=\infty$ down to $M/\varLambda =2.5$. The line in the green band is instead a fit linear in $\varLambda /M$. For comparison the dashed lines represent the quadratic (blue) and linear (green) fit through points from $M=\infty$ down to $M/\varLambda = 1.28$. Also shown by the dashed-dotted red line is a fit in this range adding to the leading-order prediction a next-to-leading term proportional to $\varLambda ^4/M^4$

**Ratios of** $\varLambda$**-parameters** The first set of results that we want to discuss are from Ref. [71] and are shown in Fig. 8. They correspond to the determination of the mass-scaling functions $\eta ^{M,{\mathcal {S}}}_{0,2}$ based on the gluonic low-energy scales ${\mathcal {S}}=t_0^{-1/2},t_c^{-1/2},w_0^{-1}$ (cf. Eq. (34)). The precise definition of these scales can be found in the given reference. The results refer to the continuum limit of the model system introduced above, i.e. ${N_{\mathrm{f}}}=2$ QCD with two heavy quarks. The RGI mass *M* of the heavy quarks is varied from about $M_c/8$ to $1.2M_c$, where $M_c$ is the RGI mass of the charm quark.^27^ The plot also includes the results for $\eta ^{M}_{0,2}$ at 1- and 4-loop order in perturbation theory [71].

As one can see from the figure, the results for the mass-scaling functions corresponding to different low-energy observables significantly differ at the smaller values of *M* in the range. As expected, however, they consistently approach each other as $M\rightarrow \infty$. In particular, for values of the mass close to the charm-quark mass all determinations well agree within errors, indicating the smallness of the O($M_c^{-2}$) corrections.

The results for $\eta ^{M}_{0,2}$ are about 1/10 for $M\approx M_c$, both in perturbation theory and non-perturbatively. In fact, within the uncertainties of roughly 10% the perturbative and non-perturbative results perfectly agree. Note that even though the relative precision on $\eta ^{M}_{0,2}$ might not seem impressive, it corresponds to an absolute error on $\eta ^{M}_{0,2}$ of about 0.01. This means, in particular, that it is reasonable to assume that the difference, $\varDelta \eta ^{M}_{0,2}$, between the non-perturbative $\eta ^{M}_{0,2}$ and its (4-loop) perturbative approximation is bounded by this error for masses $M > rsim M_c$. The scaling of the non-perturbative corrections $\varDelta \eta ^{M}_{0,2}$ to $\eta ^{M}_{0,2}$ as a function of *M* can then be assessed by studying the *M*-dependence seen in Fig. 8 and comparing the results for the different observables ${\mathcal {S}}$ [71].

Putting all this information together, the authors of Ref. [71] obtain a conservative estimate for the size of the non-perturbative contributions $\varDelta P_{0,2}$ to $P_{0,2}$. We shall not report their detailed discussion here and refer the interested reader to Ref. [71].^28^ Summarizing their conclusions, given the results for $\varDelta \eta ^{M}_{0,2}$ obtained in the model, one can safely state that the non-perturbative contributions to $P_{0,2}(M_c/\varLambda _{\overline{\mathrm{MS}}}^{(2)})$ are *at most* 2% and quite likely at the level of 0.4%. This translates into at least a 2% precision of perturbation theory in the conversion of $\varLambda$-parameters for the investigated case.

What can we conclude from this about the phenomenologically interesting case of $P_{3,4}(M_c/\varLambda _{\overline{\mathrm{MS}}}^{(4)})$? We first note that the dependence in perturbation theory of $\eta ^{M}_{\ell ,\mathrm f}$ on ${N_{\ell }}$ at fixed ${N_{\mathrm{h}}}$ is very mild. At leading order it amounts to about a 20% effect in going from ${N_{\ell }}=0$ to ${N_{\ell }}=3$ [71]. For this reason, the authors of Ref. [71] include an additional 50% contribution to their estimate for the non-perturbative corrections $\varDelta \eta ^{M}_{0,2}$ to account for the missing light-quark effects. Secondly, our intuition from both perturbative and non-perturbative considerations suggests that most likely the effects of the decoupling of a single charm quark are about half of those of two quarks. From these observations, one concludes that one can safely neglect non-perturbative effects in connecting $\varLambda _{\overline{\mathrm{MS}}}^{(3)}$ and $\varLambda _{\overline{\mathrm{MS}}}^{(4)}$ down to a precision of 1.5% or better [71].

**Ratios of hadronic quantities** The second important category of effects that we address are non-perturbative contributions from heavy quarks to dimensionless ratios of low-energy quantities. For us these are relevant in the context of setting the physical scale of the theory (see e.g. Ref. [90]). As well-known, in fact, besides the technical difficulties of computing the relevant ratios, an important issue that one faces in setting the scale in lattice QCD is the fact that one never really simulates the “real world”, where experiments are conducted. Hence, when comparing the lattice results with experiments in order to set the scale of the theory, one must “correct” the experimental quantities for effects that are not taken into account in the lattice simulations, or at least verify that these are not relevant once compared with the rest of the uncertainties. The most common examples of effects to be considered are the difference in the *u*- and *d*-quark masses, electromagnetic effects, and the unphysical number of quark flavors.

In the following we focus on the issue of estimating the effects of the charm quark in low-energy quantities, and to which extent this can be omitted in lattice simulations. From the point of view of determining $\varLambda _{\overline{\mathrm{MS}}}^{(4)}$ from the results of $\varLambda _{\overline{\mathrm{MS}}}^{(3)}$, thus, the relevant question is how accurate the determination of the physical scale is from simulations in the 3-flavor theory. This amounts to quantify how accurate it is to compute in ${N_{\mathrm{f}}}=2+1$ rather than ${N_{\mathrm{f}}}=2+1+1$ QCD, the ratios of low-energy quantities that are used to set the physical scale.

As presented in Eq. (29), the effects of heavy quarks in dimensionless ratios of low-energy quantities are expected to be of O($M^{-2}$), provided that the mass of the heavy quarks is large enough compared to the energy scales of the observables considered. In Ref. [84] a systematic study was conducted in order to assess the range of heavy-quark masses for which the O($M^{-2}$) scaling of the heavy-quark effects predicted by the effective theory actually sets in. In addition, the authors estimated the typical size of these contributions when $M\approx M_c$. For their computations, they considered the very same model of QCD with two degenerate heavy quarks previously introduced, and the same range of masses, $M\approx M_c/8-1.2M_c$. The results for several different ratios of low-energy quantities obtained in the fundamental ${N_{\mathrm{f}}}=2$ QCD theory and in the effective pure-gauge theory were compared. Note that since the effective theory is purely gluonic only gluonic quantities were considered. These, however, are all relevant observables entering realistic scale-setting determinations.

In Fig. 9 we show two examples given by the ratios $\sqrt{t_c/t_0}$ (left panel) and $\sqrt{t_0}/w_0$ (right panel), evaluated in the continuum limit. The results from ${N_{\mathrm{f}}}=2$ QCD for different values of the heavy-quark masses $M > rsim M_c/4$ are shown, as well as those from the effective pure-gauge theory which correspond to $M=\infty$. Several fits to the data are proposed. Let us focus first on the quadratic (blue) and linear (green) fits in $\varLambda /M$. Among these four fit types, the data seem to favor the leading-order effective theory predictions: $R(M) =R(\infty ) +k_R\times \varLambda ^2/M^2$, $R=\sqrt{t_c/t_0},\sqrt{t_0}/w_0$, with $k_R$ free parameters, restricted to masses $M/\varLambda > rsim 2.5$, i.e. $M > rsim M_c/2$ (blue bands). Both linear fits in $\varLambda /M$, either excluding (green bands) or including (green dashed lines) the point at $M\approx M_c/4$ have significantly larger $\chi ^2$ per degree of freedom than the previous fits (cf. Table 3 in Ref. [84]). The pure $(\varLambda /M)^2$ fits which include the $M\approx M_c/4$ results (blue dashed lines), instead, are clearly off.

Although the results do not completely exclude a linear $\varLambda /M$ dependence, the fits obtained by adding to the leading-order prediction a next-to-leading term $\propto (\varLambda /M)^{4}$ and including data down to $M\approx M_c/4$ (red dashed lines), further support the findings from the previous fits. Indeed, the close agreement for $M > rsim M_c/2$ of these fits and the leading-order predictions restricted to this range, reinforce the conclusion that the O($M^{-2}$) scaling sets in for masses $M > rsim M_c/2$, while for smaller masses higher-order contributions become relevant, or the expansion has broken down entirely.

In general the corrections induced by the heavy quarks are small. They amount to $2-3\%$ at the smallest masses in the plot, around $M_c/4$, but they are reduced significantly below the percent level, to about $0.4\%$, once $M\approx M_c$. As discussed above, we expect that this result provides a reliable estimate for the magnitude of these effects in the realistic case of the decoupling of the charm quark in ${N_{\mathrm{f}}}=2+1+1$ QCD. The simultaneous decoupling of two charm-like quarks rather than just one, likely compensates the missing effects from the light quarks; this at least in the purely gluonic quantities under consideration.

**Fig. 10:**
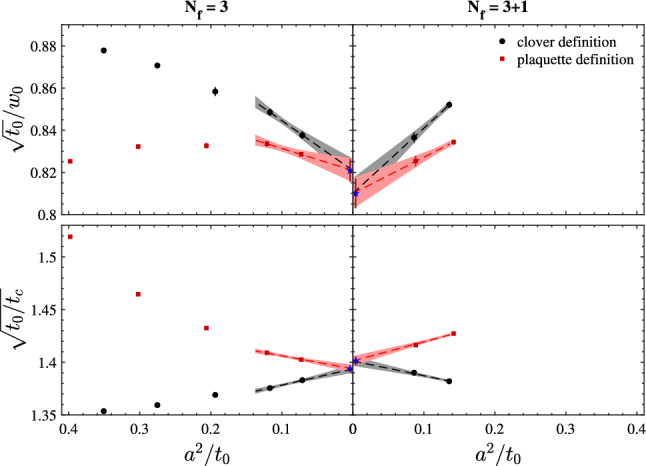
Comparison of continuum limit extrapolations of $\sqrt{t_0/t_c}$ (bottom) and $\sqrt{t_0}/w_0$ (top) of the ${N_{\mathrm{f}}}= 3 + 1$ data from Ref. [87] (right) with corresponding ${N_{\mathrm{f}}}= 3$ CLS results (left) from Refs. [20, 91] including the finest ensemble J500, cf. Ref. [92]

These expectations are confirmed by the recent results of Ref. [87]. In Fig. 10 we show their continuum limit extrapolations for different discretizations of the ratios $\sqrt{t_0/t_c}$ and $\sqrt{t_0}/w_0$. In this case, the results from ${N_{\mathrm{f}}}=3$ and ${N_{\mathrm{f}}}=3+1$ QCD are compared. Both simulations include 3 mass-degenerate quarks with a mass around the physical average mass of the *u*-, *d*- and *s*-quarks. The ${N_{\mathrm{f}}}=3+1$ simulations include in addition a fourth quark with a mass set to the physical value of the charm. By comparing the results of the two set of simulations one can directly test decoupling in a close-to-real situation. As one can see from the figure, the differences in these very precise ratios of low-energy gluonic quantities are far below the percent level and of the order of magnitude found in the model. Interestingly, the ensembles generated in Ref. [87] open the possibility to study systematically the effects of the charm quark also in fermionic low-energy observables.

**Conclusions** Let us summarize what we have learned from the studies above. 1) The ratio $\varLambda _{\overline{\mathrm{MS}}}^{(4)}/ \varLambda _{\overline{\mathrm{MS}}}^{(3)}$ can be safely computed in perturbation theory with a precision of *at least* 1.5%, and realistically much better. In fact, it is important to stress that the estimate for non-perturbative corrections to the function $P_{3,4}(M_c/\varLambda _{\overline{\mathrm{MS}}}^{(4)})$ is *very* conservative and the actual size of these effects is much likely quite smaller, i.e. at the level of $0.3\%$ or so [71]. 2) Power corrections of O($M^{-2}_c$) in low-energy observables are also found to be very small, i.e. well-below the percent level [84]. All in all, this means that $\varLambda _{\overline{\mathrm{MS}}}^{(5)}$ can be accurately predicted at the 1–2% level from $\varLambda _{\overline{\mathrm{MS}}}^{(3)}$. In this respect, we note that the competitive precision of about 0.7% on the $\alpha _s(M_Z)$ determination of Ref. [13] (to be discussed below), corresponds to an uncertainty of 3.5% on $\varLambda _{\overline{\mathrm{MS}}}^{(3)}$. This uncertainty is also conservative. In conclusions, there is plenty of room for improvement within ${N_{\mathrm{f}}}=3$ QCD.

## Renormalization by decoupling

### The QCD coupling from ${N_{\mathrm{f}}}=3$ QCD

In the previous section we established that by relying on perturbative decoupling relations for the charm and bottom quarks, precise determinations of $\varLambda _{\overline{\mathrm{MS}}}^{(5)}$ are possible from results in the ${N_{\mathrm{f}}}=3$ flavor theory. In order to be able to make this statement the systematic studies on the non-perturbative effects induced by the charm quark in low-energy quantities have been instrumental.

Having this settled, as discussed in detail in Sect. 2, the main challenges for an accurate determination of $\alpha _{\mathrm{s}}$ on the lattice are: (1) controlling discretization errors in continuum limit extrapolations of the chosen non-perturbative coupling(s), and (2) estimating the uncertainties associated with the use of perturbation theory in extracting the $\varLambda$-parameter. In this respect, the combined application of finite-volume renormalization schemes and finite-size scaling techniques has proven to be extremely effective in dealing with these difficulties, paving the way for robust and precise lattice determinations of the QCD coupling.

The determination of Ref. [13], in particular, relies on these techniques to compute $\varLambda _{\overline{\mathrm{MS}}}^{(3)}$. The calculation reaches a final precision on $\varLambda _{\overline{\mathrm{MS}}}^{(3)}$ of about $3.5\%$, which translates into a $0.7\%$ uncertainty on $\alpha _{\mathrm{s}}(M_Z)$. The strength of this result lies in the fact that all systematic uncertainties are carefully kept under control at this competitive level of accuracy. The calculation is therefore a prominent example of the current state-of-the-art determinations of $\alpha _{\mathrm{s}}$ from the lattice [3]. Below we want to briefly recall the main steps that led to this result in order to understand what the challenges are in improving on it. For a more detailed presentation we refer the interested reader to the original references [13, 31, 32, 50] and reviews [93–97].

The $\varLambda _{\overline{\mathrm{MS}}}^{(3)}$ determination of Ref. [13] was obtained from the study of the non-perturbative running of some convenient finite-volume schemes from a scale of about $0.2\,\mathrm{GeV}$ to roughly $70\,\mathrm{GeV}$. The very high energies reached non-perturbatively allowed for a systematic and robust assessment of the uncertainties related to the application of perturbation theory. This study has been presented in Sect. 2.3.1, where the high-energy end of the running in the SF-schemes and the result for $\varLambda _{\overline{\mathrm{MS}}}^{(3)}/\mu _{0}$ with $\mu _{0}\approx 4.3\,\mathrm{GeV}$, have been discussed in detail. The rest of the determination is built on the following steps.

**Fig. 11:**
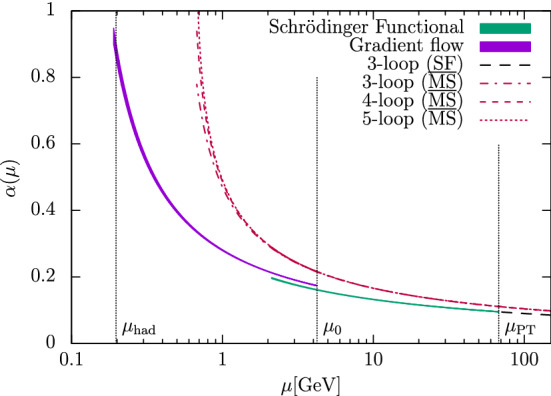
Running couplings of ${N_{\mathrm{f}}}= 3$ QCD from integrating the non-perturbative $\beta$-functions in the SF- and GF-schemes [31, 50]. They are matched non-perturbatively at the scale $\mu _0$ defined by ${\bar{g}}^2_{\mathrm{SF_{\nu =0}}}(\mu _0) = 2.012$ by computing ${\bar{g}}^2_{\mathrm{GF}}(\mu _0/2)=2.6723(64)$ [50]. The scales $\mu _{\mathrm{PT}}=16\mu _0$ and $\mu _{\mathrm{had}}$ defined by ${\bar{g}}^2_{\mathrm{GF}}(\mu _{\mathrm{had}})=11.31$ are also shown, as well as the perturbative prediction for the $\mathrm{SF}_{\nu =0}$-coupling for $\mu >\mu _{\mathrm{PT}}$ using the 3-loop $\beta$-function. The red curves correspond to the results for $\alpha ^{(3)}_{\overline{\mathrm{MS}}}(\mu )$ obtained from $\varLambda _{\overline{\mathrm{MS}}}^{(3)} =341(12)\,\mathrm{MeV}$ [13], considering different perturbative orders for the $\beta$-function in the $\overline{\mathrm{MS}}$-scheme

Firstly, we have the computation of the lower end of the running and corresponding determination of the ratio of finite-volume scales $\mu _{0}/\mu _{\mathrm{had}}$ with $\mu _{\mathrm{had}}\approx 200\,\mathrm{MeV}$ [50]. For this step, a novel finite-volume coupling defined in terms of the Yang-Mills gradient flow was employed. This allowed for reaching much greater precision than otherwise possible using the schemes considered at high energy [98]. Note that a non-perturbative matching between the finite-volume schemes at $\mu _0$ was performed in order to continue the running at lower energies. For illustration, we show in Fig. 11 the non-perturbative running of the finite-volume couplings over the energy range covered, together with the results for the coupling in the $\overline{\mathrm{MS}}$-scheme obtained from the corresponding determination of $\varLambda _{\overline{\mathrm{MS}}}^{(3)}$ (see below).

In a second step, the relation $\mu _{\mathrm{had}}/f_{\pi K}$ was established passing through the intermediate technical scale $\mu _{\mathrm{ref}}^*=1/\sqrt{8t^*_0}$ [13]. Here, $f_{\pi K}\equiv {1 \over 3} (2f_K+f_\pi )$ is a convenient combination of the pion and kaon decay constants, while the scale $\mu _\mathrm{ref}^*$ is given in terms of the flow time $t^*_0$ defined in the SU(3) flavor-symmetric limit [91]. This step involved a combination of small-volume and large-volume simulations in the hadronic regime [13]. From the experimental value of $f_{\pi K}$ the precise physical units for $\mu _{\mathrm{had}}$ could be inferred and hence those of $\varLambda _{\overline{\mathrm{MS}}}^{(3)}$. Finally, perturbation theory was used for the functions $P_{3,4}(M_c/\varLambda _{\overline{\mathrm{MS}}}^{(4)})$ and $P_{4,5}(M_b/\varLambda _{\overline{\mathrm{MS}}}^{(5)})$ to obtain $\varLambda _{\overline{\mathrm{MS}}}^{(5)}$ and from this $\alpha _{\mathrm{s}}(M_Z)$. Splitting the determination of $\varLambda _{\overline{\mathrm{MS}}}^{(5)}$ over the above steps was the key to keep all systematic errors under control. With the proper choice of observables and techniques the hard multi-scale problem of relating the low- and high-energy sectors of QCD could be solved in full confidence.

**Fig. 12:**
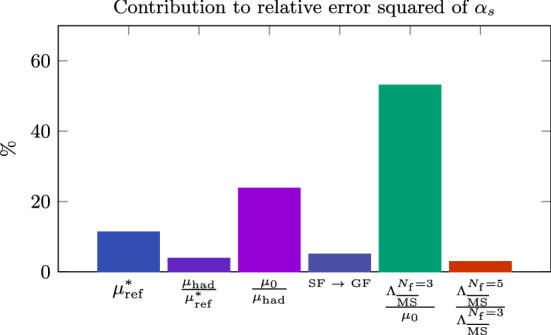
Contribution in percentage to the relative error squared of $\alpha _{\mathrm{s}}(M_Z)$ from the different steps of the determination of Ref. [13] (cf. text for more details). As the reader can see, the dominant source of uncertainty is the non-perturbative running at high energy $\mu \approx 4-70\,\mathrm{GeV}$ ($\varLambda _{\overline{\mathrm{MS}}}^{({N_{\mathrm{f}}}=3)}/\mu _0$) [31, 32], followed by the running at low energy $\mu \approx 0.2-4\,\mathrm{GeV}$ ($\mu _0/\mu _{\mathrm{had}}$) [50], and by scale setting ($\mu _{\mathrm{ref}}^*$) [91]. Note that the error from decoupling ($\varLambda _{\overline{\mathrm{MS}}}^{(5)}/ \varLambda _{\overline{\mathrm{MS}}}^{(3)}$) is only perturbative. However, even adding the very conservative uncertainty estimated in Sect. 3.3.2 for the non-perturbative contributions to the decoupling of the charm quark, the total error is still dominated by the one from the running

It is now instructive to look at the error budget of this $\alpha _{\mathrm{s}}$ determination. This is given in Fig. 12 which shows the contribution in percentage to the relative error squared on $\alpha _{\mathrm{s}}(M_Z)$ from the different steps described above [93]. As it is clear from the figure, the main source of uncertainty comes from the determination of the non-perturbative running from $\mu _{\mathrm{had}}\approx 0.2\,\mathrm{GeV}$ up to $\mu _{\mathrm{PT}}\approx 70\,\mathrm{GeV}$, where perturbation theory is applied to extract $\varLambda _{\overline{\mathrm{MS}}}^{(3)}/\mu _{\mathrm{PT}}$ (cf. Sect. 2.3.1). In particular, the error accumulated by running from $\mu _{0}$ to $\mu _{\mathrm{PT}}$ (labeled as $\varLambda _{\overline{\mathrm{MS}}}^{({N_{\mathrm{f}}}=3)}/\mu _0$ in the plot) contributes roughly 60% of the total budget.

It is important to recall at this point that the error coming from the running is completely dominated by statistical uncertainties. In particular, thanks to the fact that $\mu _{\mathrm{PT}}\approx 70\,\mathrm{GeV}$ was reached non-perturbatively, the uncertainties due to the use of perturbation theory are well below the statistical errors (cf. Sect. 2.3.1). In this respect, we want to stress the important difference between this and the majority of other lattice determination of $\varLambda _{\overline{\mathrm{MS}}}^{(3)}$, where perturbation theory is applied at scales $\mu _{\mathrm{PT}}\lesssim 2-3\,\mathrm{GeV}$. In these cases, a large fraction of the final error comes from the *systematic* uncertainties related to the truncation of the perturbative series and possible remnants of non-perturbative contributions (cf. Ref. [3]). As we have seen, estimating these systematics reliably is very challenging, particularly so when precision is desired but the accessible range of scales is limited to low energy. In this situation, a reduction of the final uncertainties is highly non-trivial, and can eventually come only from reaching significantly higher energy scales. Without a step-scaling approach this is in practice extremely demanding in QCD given the present computational and algorithmic capabilities.^29^

In the case of the step-scaling method, on the other hand, reducing the uncertainties on the current $\alpha _{\mathrm{s}}(M_Z)$ determination is a question of reducing the statistical uncertainties coming from the running of the coupling(s) in ${N_{\mathrm{f}}}=3$ QCD. This is in principle a straightforward task. However, reducing the total error by an *important* factor, say a factor 2 or so, is yet a non-trivial challenge from the computational point of view.

Rather than following a brute force approach for the reduction of the error in the computation of the running in ${N_{\mathrm{f}}}=3$ QCD, in the following we shall discuss a novel strategy which promises the desired error reduction in a significantly cheaper way [14]. It is based on the ideas presented in the previous section on heavy-quark decoupling. The distinct feature of the approach is that one can replace the non-perturbative computation of the running in ${N_{\mathrm{f}}}=3$ QCD needed to determine $\varLambda _{\overline{\mathrm{MS}}}^{(3)}$ with the running in the pure Yang–Mills theory.^30^

### The coupling from decoupling

#### General strategy and master formula

Let us begin by considering QCD with ${N_{\mathrm{f}}}$ flavors of heavy quarks of RGI mass *M*. In this theory all quarks are massive and there are no light quarks. As we have seen in Sect. 3.1.1, as the mass *M* becomes larger and larger this theory is expected to be approximated better and better by an effective theory given by the pure Yang-Mills theory. In particular, once the $\varLambda$-parameters of the fundamental and effective theory are properly matched, dimensionless low-energy observables can be computed in the effective theory up to corrections of O($M^{-n}$), where, in general, $n=2$ if the theories are matched at leading order.

The decoupling of heavy quarks is also valid for the interesting case of couplings defined in massive renormalization schemes [23, 73]. This is a direct consequence of the fact that such couplings are defined in terms of dimensionless observables in the massive theory. Following the notation of Sect. 2.2.2, we indicate the generic renormalized massive coupling in the ${N_{\mathrm{f}}}$-flavor theory as ${\bar{g}}^{({N_{\mathrm{f}}})}_{{{\mathcal {O}}}}(\mu ,M)$, where ${{\mathcal {O}}}$ denotes the short-distance observable used to define the coupling (cf. Eq. (3)), while $\mu$ is the renormalization scale. From the decoupling of heavy quarks applied to the observable ${{\mathcal {O}}}$ it follows that,

35 $$\begin{aligned} {\bar{g}}^{({N_{\mathrm{f}}})}_{{\mathcal {O}}}(\mu ,M)= {\bar{g}}^{(0)}_{{\mathcal {O}}}(\mu )+\mathrm{O}(M^{-2})\,, \end{aligned}$$

where ${\bar{g}}^{(0)}_{{\mathcal {O}}}(\mu )$ refers to the corresponding coupling in the pure Yang-Mills theory evaluated at the *same* physical scale $\mu$. Note that here and below we shall loosely denote as $\mathrm{O}(M^{-k})$ terms, contributions that contain terms of $\mathrm{O}((\varLambda /M)^{k})$ as well as $\mathrm{O}((\mu /M)^{k})$.

The decoupling relation, Eq. (35), can be equivalently recast in terms of the renormalization scales $\mu$ implicitly defined by the couplings. Specifically, given a numerical value for the coupling $g_M$, we define the scales $\mu _\mathrm{dec}^{({N_{\mathrm{f}}})}$ and $\mu _{\mathrm{dec}}^{(0)}$ through

36 $$\begin{aligned} {\bar{g}}^{({N_{\mathrm{f}}})}_{{\mathcal {O}}}(\mu _{\mathrm{dec}}^{({N_{\mathrm{f}}})},M)= g_M ={\bar{g}}^{(0)}_{{\mathcal {O}}}(\mu _{\mathrm{dec}}^{(0)})\,. \end{aligned}$$

From the theory of decoupling it then follows that

37 $$\begin{aligned} \mu _{\mathrm{dec}}^{(0)}= \mu _{\mathrm{dec}}^{({N_{\mathrm{f}}})}+\mathrm{O}(M^{-2})\,. \end{aligned}$$

We stress again that we assume that the $\varLambda$-parameters in the two theories are properly matched.

From this basic observation the master formula proposed in Ref. [14] follows. We start by considering the relation in Eq. (27), and take for the low-energy scale ${\mathcal {S}}$ the renormalization scale $\mu _{\mathrm{dec}}$ defined above in terms of the given couplings. In formulas,

38 $$\begin{aligned} {\varLambda ^{(0)}_{\overline{\mathrm{MS}}}\over \mu _{\mathrm{dec}}^{(0)}}= P_{0,\mathrm f}(M/\varLambda ^{({N_{\mathrm{f}}})}_{\overline{\mathrm{MS}}}) {\varLambda ^{({N_{\mathrm{f}}})}_{\overline{\mathrm{MS}}}\over \mu _\mathrm{dec}^{({N_{\mathrm{f}}})}}+\mathrm{O}(M^{-2})\,, \end{aligned}$$

where for later convenience we took the $\varLambda$-parameters in the $\overline{\mathrm{MS}}$-scheme.^31^ Now, rather than interpreting the above equation as a matching relation for the $\varLambda$-parameters that defines the function $P_{0,\mathrm f}$, we shall turn tables and use it to *predict* the ratio ${\varLambda ^{({N_{\mathrm{f}}})}_{\overline{\mathrm{MS}}}/\mu _{\mathrm{dec}}^{({N_{\mathrm{f}}})}}$ in terms of ${\varLambda ^{(0)}_{\overline{\mathrm{MS}}}/ \mu _{\mathrm{dec}}^{(0)}}$.

To this end, we first replace the function $P_{0,\mathrm f}$ with its perturbative expansion $P_{0,\mathrm f}^{\mathrm{PT}}$ in the ${\overline{\mathrm{MS}}}$-scheme to some order *n* (cf. Eqs. (24), (10) and Sects. 3.2.2, 3.3.1),

39 $$\begin{aligned} P_{0,\mathrm f}(M/\varLambda ^{({N_{\mathrm{f}}})}_{\overline{\mathrm{MS}}})= P^{\mathrm{PT}}_{0,\mathrm f}(M/\varLambda ^{({N_{\mathrm{f}}})}_{\overline{\mathrm{MS}}})+ \mathrm{O}(g_*^{2n-2},M^{-2})\,. \end{aligned}$$

We therefore have that,

40 $$\begin{aligned} {\varLambda ^{({N_{\mathrm{f}}})}_{\overline{\mathrm{MS}}}\over \mu _{\mathrm{dec}}^{({N_{\mathrm{f}}})}} P^{\mathrm{PT}}_{0,\mathrm f}(M/\varLambda ^{({N_{\mathrm{f}}})}_{\overline{\mathrm{MS}}})= {\varLambda ^{(0)}_{\overline{\mathrm{MS}}}\over \mu _{\mathrm{dec}}^{(0)}} +\mathrm{O}(g_*^{2n-2},M^{-2}). \end{aligned}$$

As a second step, using Eq. (4) and the definition in Eq. (36) we write,

41 $$\begin{aligned} {\varLambda ^{(0)}_{\overline{\mathrm{MS}}}\over \mu _{\mathrm{dec}}^{(0)}} = {\varLambda ^{(0)}_{\overline{\mathrm{MS}}}\over \varLambda ^{(0)}_{{\mathcal {O}}}}\,\varphi ^{(0)}_{\mathrm{g,{{\mathcal {O}}}}}(g_M)\,, \end{aligned}$$

which only involves quantities in the pure-gauge theory. We recall that ${\mathcal {O}}$ appearing here is the observable used to calculate the coupling in Eq. (36). Moreover, as discussed in Sect. 2.2.2, the change of scheme given by the ratio ${\varLambda ^{(0)}_{\overline{\mathrm{MS}}}/ \varLambda ^{(0)}_{{\mathcal {O}}}}$ can be computed exactly through a 1-loop calculation (cf. Eq. (8)).

Finally, introducing the dimensionless variables,

42 $$\begin{aligned} \rho \equiv {\varLambda ^{({N_{\mathrm{f}}})}_{\overline{\mathrm{MS}}}/ \mu _\mathrm{dec}^{({N_{\mathrm{f}}})}}\,, \qquad z\equiv {M/\mu _{\mathrm{dec}}^{({N_{\mathrm{f}}})}}\,, \end{aligned}$$

using Eqs. (40) and (41) we arrive at the master equation,

43 $$\begin{aligned} \rho \,P^{\mathrm{PT}}_{0,\mathrm f}(z/\rho ) = {\varLambda ^{(0)}_{\overline{\mathrm{MS}}}\over \varLambda ^{(0)}_{{\mathcal {O}}}}\,\varphi ^{(0)}_{\mathrm{g,{{\mathcal {O}}}}}(g_M) +\mathrm{O}(g_*^{2n-2},M^{-2}), \end{aligned}$$

which can be solved for $\rho$ once the pure-gauge function $\varphi ^{(0)}_{\mathrm{g,{{\mathcal {O}}}}}(g_M)$ is known. As promised, the master formula allows us to replace the non-perturbative computation of the running of the coupling from the low-energy scale $\mu _{\mathrm{dec}}$ up to infinite energy in ${N_{\mathrm{f}}}$-flavor QCD with the corresponding running in the pure-gauge theory. A few remarks are in order at this point.

First of all, it is important to stress the fact that Eq. (43) is *exact* in the limit where $M\rightarrow \infty$. In this limit both the perturbative O($g_*^{2n-2}$) corrections and the non-perturbative O($M^{-2}$) contributions vanish. The basic idea that is applied in this strategy is in fact similar to when we extract $\varLambda _{\overline{\mathrm{MS}}}^{(4)}$ from $\varLambda _{\overline{\mathrm{MS}}}^{(3)}$ by replacing the non-perturbative function $P_{3,4}(M_c/\varLambda ^{(4)}_{\overline{\mathrm{MS}}})$ with its perturbative approximation to some order, neglecting both higher-order terms and non-perturbative corrections. The crucial difference in the present case is that the approximation can be made systematically better by considering larger values of *M*, which, at least in principle, is a free parameter of the strategy. In this respect, note that the perturbative corrections to $P_\mathrm{0, f}(M/\varLambda ^{({N_{\mathrm{f}}})}_{\overline{\mathrm{MS}}})$ only depend on the value of $M/\varLambda ^{({N_{\mathrm{f}}})}_{\overline{\mathrm{MS}}}$, while the choice of scale $\mu _{\mathrm{dec}}^{({N_{\mathrm{f}}})}$ does not matter. From the results presented in Sect. 3.2.2, we infer that the perturbative errors due to the truncation of the perturbative series for $P_{\mathrm{0, f}}(M/\varLambda ^{({N_{\mathrm{f}}})}_{\overline{\mathrm{MS}}})$ are already small for $M/\varLambda _{\overline{\mathrm{MS}}}^{({N_{\mathrm{f}}})}\approx 5$, for the relevant values of ${N_{\mathrm{f}}}$. On the other hand, controlling the non-perturbative O($M^{-2}$) terms requires to have both O($(\varLambda ^{({N_{\mathrm{f}}})}_{\overline{\mathrm{MS}}}/M)^2$) and O($(\mu _\mathrm{dec}^{({N_{\mathrm{f}}})}/M)^2$) terms under control. Whether this is possible in practice must be assessed by carefully studying the limit $M\rightarrow \infty$ with the accessible values of *M*.

In comparing Eqs. (27) and (38), the attentive reader might have noticed that we omitted the *M*-dependence on the scale $\mu ^{({N_{\mathrm{f}}})}_{\mathrm{dec}}$. This was intentional. As we shall see in the later subsection, in practice it is convenient in fact to define a single scale, $\mu ^{({N_{\mathrm{f}}})}_{\mathrm{dec}}$, common to all the ${N_{\mathrm{f}}}$-flavor theories defined by the different values of *M*.^32^ This means that, as anticipated by our notation in Eq. (36), we will have different values for $g_M$ depending on the value of *M* considered. The value of $g_M$ is found by computing ${\bar{g}}^{({N_{\mathrm{f}}})}_{{\mathcal {O}}}(\mu ^{({N_{\mathrm{f}}})}_{\mathrm{dec}},M)$ for the given *M* at the common scale $\mu ^{({N_{\mathrm{f}}})}_{\mathrm{dec}}$. In lattice QCD, setting a common scale among the theories with different *M* values can be achieved via the bare parameters. This amounts to consider the very same lattice discretization and establish a line of constant physics along which $\mu _\mathrm{dec}^{({N_{\mathrm{f}}})}$ is kept fixed. The massive couplings are then evaluated at matching values of the bare coupling along this line of constant physics for the different bare quark masses corresponding to the target RGI masses. From their continuum limit extrapolations we find the values $g_M$.

Finally, in order to extract $\varLambda _{\overline{\mathrm{MS}}}^{({N_{\mathrm{f}}})}$ from the results for $\rho$ determined from Eq. (43) it is necessary to know the value of $\mu ^{({N_{\mathrm{f}}})}_{\mathrm{dec}}$ in physical units. This is obtained by establishing the relation $\mu ^{({N_{\mathrm{f}}})}_{\mathrm{dec}}/\mu ^{({N_{\mathrm{f}}})}_\mathrm{phys}$, where $\mu ^{({N_{\mathrm{f}}})}_{\mathrm{phys}}$ denotes a convenient low-energy scale computed in ${N_{\mathrm{f}}}$-flavor QCD at *physical* values of the quark masses. The scale $\mu ^{({N_{\mathrm{f}}})}_{\mathrm{phys}}$ can thus be related to its experimental counterpart. Of course, it goes without saying that in order to be able to set the scale accurately in terms of experimentally measurable quantities, as well as to perturbatively match $\varLambda _{\overline{\mathrm{MS}}}^{({N_{\mathrm{f}}})}$ and $\varLambda _{\overline{\mathrm{MS}}}^{(5)}$, we must consider ${N_{\mathrm{f}}}=3$ or 4.

#### Another hard multi-scale problem?

The general strategy presented above is certainly very compelling. As any other strategy, though, in practical implementations it comes with its challenges. In particular, in order to have all systematic effects under control, it is necessary to carefully address how to accommodate in lattice simulations the different scales that enter the problem. As we shall see, a naive approach can easily end up facing severe limitations.

In the general situation, first of all, the space-time volume has to be large enough for finite-volume effects to be under control in all relevant observables. This means that the infrared cutoff set by the linear extent *L* of the lattice must be much smaller than all other scales. Secondly, in order to have small decoupling corrections in Eq. (43) we must have that the heavy-quark mass *M* is larger than all other physical scales, as in particular $\mu ^{({N_{\mathrm{f}}})}_{\mathrm{dec}}$ and $\varLambda _{\overline{\mathrm{MS}}}^{({N_{\mathrm{f}}})}$. Note that although $\mu ^{({N_{\mathrm{f}}})}_{\mathrm{dec}}$ is in principle arbitrary, in practice it is not convenient to take this scale to be much larger than $\varLambda _{\overline{\mathrm{MS}}}^{({N_{\mathrm{f}}})}\sim \mu ^{({N_{\mathrm{f}}})}_{\mathrm{phys}}$. Last but not least, all scales have to be well below the ultraviolet cutoff set by the lattice spacing. Putting all these constraints together we find (cf. Eq. (2)),

44 $$\begin{aligned} L^{-1}\ll \mu ^{({N_{\mathrm{f}}})}_{\mathrm{phys}} \sim \varLambda _{\overline{\mathrm{MS}}}^{({N_{\mathrm{f}}})}\sim \mu ^{({N_{\mathrm{f}}})}_{\mathrm{dec}} \ll M \ll a^{-1}\,. \end{aligned}$$

It is clear from this series of inequalities that having all these scales comfortably resolved on a *single* lattice is challenging and requires a very large *L*/*a*. Just to give an example, if one attempts the calculations using a state-of-the-art large-volume ensemble with, say, $L/a=100$, $m_\pi L=4$, $m_\pi =140\,\mathrm{MeV}$, which results in $a\approx 0.056\,\mathrm{fm}$, Eq. (44) translates into $M\ll 3.5\,\mathrm{GeV}$.

#### Finite-volume couplings rescue us again

Some of the constraints encoded in Eq. (44) can be lifted by considering for the coupling in Eq. (35) a finite-volume scheme, i.e. ${\bar{g}}_{{\mathcal {O}}}(\mu ,M)\equiv {\bar{g}}_{{\mathcal {O}}}(L^{-1},M)$ (cf. Sect. 2.2.1). With this choice, the determination of ${\bar{g}}_{{\mathcal {O}}}(\mu ^{({N_{\mathrm{f}}})}_{\mathrm{dec}},M)$ does not require the physical volume to be large. Furthermore, if the decoupling scale $\mu ^{({N_{\mathrm{f}}})}_{\mathrm{dec}}$ is also defined in terms of a finite-volume coupling, i.e. $\mu ^{({N_{\mathrm{f}}})}_{\mathrm{dec}}\equiv L^{-1}_{\mathrm{dec}}$, one has some additional freedom in the choice of the value of $\mu ^{({N_{\mathrm{f}}})}_{\mathrm{dec}}$. Any sizable scale separation between $\mu ^{({N_{\mathrm{f}}})}_{\mathrm{dec}}$ and the hadronic scale $\mu ^{({N_{\mathrm{f}}})}_\mathrm{phys}$ can in fact be bridged through step-scaling within the ${N_{\mathrm{f}}}$-flavor theory (cf. Sect. 2.2.1). Taking $\mu ^{({N_{\mathrm{f}}})}_{\mathrm{dec}}$ larger at fixed $z=M/\mu ^{({N_{\mathrm{f}}})}_{\mathrm{dec}}$, allows us to reach larger values of $M/\varLambda _{\overline{\mathrm{MS}}}^{({N_{\mathrm{f}}})}$, as well as to profit from simulating at smaller values of *a*. On the other hand, the larger $\mu ^{({N_{\mathrm{f}}})}_{\mathrm{dec}}$ is, the smaller is the range of energy scales for which, through Eq. (43), the running of the coupling in the ${N_{\mathrm{f}}}$-flavor theory is replaced by that in pure-gauge. In practice, choosing $\mu ^{({N_{\mathrm{f}}})}_{\mathrm{dec}}=\mathrm{O}(1\,\mathrm{GeV})$ is a good compromise.

By employing finite-volume couplings and scales the only constraints that we have to meet are to have small decoupling corrections in Eq. (43), and to keep discretization errors under control. The first condition requires $z=ML_{\mathrm{dec}}\gg 1$, while discretization effects are small once $aM\ll 1$ and $a/L_\mathrm{dec}\ll 1$. Putting these inequalities together we find the conditions

45 $$\begin{aligned} L_{\mathrm{dec}}/a\gg z\gg 1. \end{aligned}$$

If we take, say, $\mu ^{({N_{\mathrm{f}}})}_{\mathrm{dec}}=1\,\mathrm{GeV}$ and $L_\mathrm{dec}/a=50$, then $M\ll 50\,\mathrm{GeV}$. In summary, using finite-volume schemes, for a given set of lattice sizes *L*/*a* we can reach much finer lattice spacings *a*, as the extent of the lattice *L* does not have to be large in physical units. Smaller *a* values allow us to consider larger *M* values, while having *aM* reasonably small. Larger values of *M* make for a more precise approximation $P_\mathrm{0,f}^{\mathrm{PT}}(M/\varLambda )$. Still, large *z* values have to be reached in order to control non-perturbative decoupling corrections.

**Heavy-quark decoupling in a finite volume** As pointed out for the case of computing the running through a step-scaling procedure (cf. Sect. 2.2.1), the choice of finite-volume coupling is dictated by several technical aspects, as for instance, statistical precision and discretization errors. For the strategy based on decoupling an additional factor becomes relevant which is the size of non-perturbative contributions in the decoupling of heavy quarks (cf. Eq. (35)). In a finite volume, the situation can be quite different from one coupling definition to another, as even the leading power in $M^{-1}$ may be different.

Most finite-volume couplings that are used in practice are based on the QCD Schrödinger functional (SF) [36–38]. In the SF the quark and gluon fields satisfy Dirichlet boundary conditions at the space-time boundaries located at $x_0=0$ and *T*, where *T* is the temporal extent of the space-time volume (cf. Eqs. (47)–(48)). These boundary conditions guarantee many compelling features [36, 37]. However, they come with the price of having, for instance, additional discretization effects of O(*a*) [36, 101]. Using Symanzik effective theory these can be understood as dimension 4 counterterms localized at the space-time boundaries [36, 101]. In close analogy with Symanzik effective theory an analysis of the effective Lagrangian for heavy quarks in the presence of SF boundary conditions shows that the same boundary fields appear as O($M^{-1}$) counterterms. More precisely, considering the relevant case ${N_{\ell }}=0$, the Lagrangian ${\mathcal {L}}_1$ in Eq. (17) has the form (cf. Eq. (18))

46 $$\begin{aligned} {\mathcal {L}}_1= & {} \omega _b(g) \big [ {\mathcal {B}}(0)+{\mathcal {B}}(T)\big ]\,, \nonumber \\ {\mathcal {B}}(x_0)= & {} -{1\over g^2}\int \mathrm{d}{\varvec{x}}\, \mathrm{tr}\{F_{0k}(x)F_{0k}(x)\}|_{x_0}\,, \end{aligned}$$

where $F_{\mu \nu }(x)$ is the gluon-field strength tensor, while $\omega _b$ is a coefficient function that can be fixed by matching with the fundamental ${N_{\mathrm{f}}}$-flavor theory. Note that for simplicity we listed the only gluonic operator that is relevant for the class of the SF boundary conditions normally employed (cf. Eq. (47)). As a result, if couplings based on the SF are considered, the decoupling relations Eqs. (35), (43) must be corrected to have leading O($M^{-1}$) corrections rather than O($M^{-2}$) [14, 41, 102].^33^ On the other hand, finite-volume couplings defined through some variant of periodic boundary conditions, as for instance regular periodic [48] or twisted [105, 106] boundary conditions, have leading decoupling corrections of O($M^{-2}$), as observables in infinite space-time.

There are several possibilities to deal with the issue of O($M^{-1}$) corrections in SF-based couplings. A straightforward one is to consider a sufficiently large time extent *T*, and take for the observable ${{\mathcal {O}}}$ that defines the couplings fields which are localized in the middle of the space-time manifold, i.e. at $x_0=T/2$. This maximizes the distance from the boundaries and therefore the correlation between ${{\mathcal {O}}}$ and the fields responsible for O($M^{-1}$) effects. In fact, at low energy (i.e. relatively large physical *L* and *T*) the O($M^{-1}$) contaminations are expected to be exponentially suppressed with the distance of ${{\mathcal {O}}}$ from the boundaries.

More elegant solutions have been proposed which eliminate entirely the issue. For example, one could consider for the heavy quarks a twisted rather than a standard mass [107–110]. In this case, one can show that ${\mathcal {L}}_1=0$ and the decoupling in the SF is realized with O($M_{\mathrm{tw}}^{-2}$) corrections, where $M_{\mathrm{tw}}$ is the heavy twisted mass of the quarks [102]. Equivalent in the continuum is the situation where the heavy quarks have a standard mass but the SF boundary conditions are chirally rotated [102, 111, 112] (see also Refs. [113–118]). The issue with these solutions is that they require an even number of flavors ${N_{\mathrm{f}}}$. They are therefore a promising approach for the case of ${N_{\mathrm{f}}}=4$ QCD. For ${N_{\mathrm{f}}}=3$, one may consider having a doublet of twisted-mass quarks and a regular massive quark (or equivalently a doublet of chirally rotated quarks and a regular SF quark (see e.g. Ref. [118])). This would reduce the O($M^{-1}$) contributions to those of only a single flavor.

Another possibility that we want to mention, which is valid for any choice of ${N_{\mathrm{f}}}$, is to match the effective and fundamental theory at O($M^{-1}$) [119]. In other words, by equating the results for some convenient observable in the effective and fundamental theory one can determine the coefficient $\omega _b$ appearing in Eq. (46). Once this is determined, the O($M^{-1}$) terms can be taken into account in the effective theory by computing the insertion of the counterterm in Eq. (46) in the relevant observables. This guarantees that the decoupling corrections are of O($M^{-2}$).

### $\varLambda _{\overline{\mathrm{MS}}}^{(3)}$ from the decoupling of heavy quarks

#### Definitions

In this subsection we present the results of Ref. [14] where the master formula, Eq. (43), was first applied for the computation of $\varLambda _{\overline{\mathrm{MS}}}^{(3)}$. The study considers ${N_{\mathrm{f}}}=3$ QCD which is set on the lattice in terms of non-perturbatively O(*a*)-improved Wilson quarks and the tree-level Symanzik O($a^2$)-improved gauge action [120].^34^

The theory is defined in a finite volume with time extent *T* and spatial size *L*. The quark fields $\psi ,{\overline{\psi }}$ and the gauge field $A_\mu$ satisfy Dirichlet boundary conditions in the time direction, specifically $P_\pm \equiv {1 \over 2} (1\pm \gamma _0)$ [36, 37, 101],

47 $$\begin{aligned}&P_+\psi (x)|_{x_0=0}=0 = P_-\psi (x)|_{x_0=T}\,, \nonumber \\&{\overline{\psi }}(x)P_-|_{x_0=0}=0= {\overline{\psi }}(x)P_+|_{x_0=T}\,, \nonumber \\&A_{k}(x)|_{x_0=0,T}=0\,, \end{aligned}$$

$k=1,2,3$, while in the spatial directions we have

48 $$\begin{aligned}&\psi (x+{\hat{k}}L)=e^{i\over 2}\psi (x)\,, \quad {\overline{\psi }}(x+{\hat{k}}L)={\overline{\psi }}(x)e^{-i\over 2}\,, \nonumber \\&A_\mu (x+{\hat{k}}L)=A_\mu (x)\,. \end{aligned}$$

The finite-volume couplings that we consider in the following are constructed in terms of the gradient flow field $B_\mu (t,x)$ which is defined by the equations [47, 121],

49 $$\begin{aligned} \partial _t B_\mu (t,x)=D_\nu G_{\nu \mu }(t,x)\,, \quad B_\mu (0,x)=A_\mu (x)\,, \end{aligned}$$

where $t>0$ is the flow time and

50 $$\begin{aligned} G_{\mu \nu }=\partial _\mu B_\nu - \partial _\nu B_\mu +[B_\mu ,B_\nu ]\,, \end{aligned}$$

is the flow-field strength tensor. On the lattice several discretizations of the GF equations have been considered, here we employ the Symanzik O($a^2$)-improved definition proposed in Ref. [122], also known as Zeuthen flow.

Gauge-invariant composite fields made out of the flow field $B_\mu (t,x)$ are automatically finite [123], and are thus ideal quantities to define renormalized couplings [47–49]. The definition to which we apply decoupling (cf. Eq. (35)) is the massive finite-volume scheme given by

51 $$\begin{aligned}{}[{\bar{g}}^{(3)}_{\mathrm{GFT}}(\mu ,M)]^2\!\equiv \! {t^2\over {\mathcal {N}}} \langle E_{\mathrm{mag}}(t,x)\rangle _{\mathrm{SF},Q=0}\big |_{T=2L}^{x_0=L,\mu \!=\!L^{-1},\sqrt{8t}=cL}\,, \end{aligned}$$

where ${\mathcal {N}}$ is a normalization constant [49] and

52 $$\begin{aligned} E_\mathrm{mag}(t,x)=-{1\over 2}\mathrm{tr}\{G_{kl}(t,x)G_{kl}(t,x)\} \end{aligned}$$

is the magnetic component of the energy density of the flow field $B_\mu (t,x)$. On the lattice, we define $E_{\mathrm{mag}}$ in terms of the O($a^2$)-improved definition given in Ref. [122].

Note how in Eq. (51) the renormalization scale $\mu$ is set in terms of the finite spatial extent *L*, as appropriate for a finite-volume coupling. In order for the coupling to depend on a single scale (apart from *M*), the flow time *t* is also linked to *L* through the constant *c* [48, 49]. The value of this constant is in principle arbitrary, but experience suggests that $c=0.3$ is a good compromise between statistical precision and discretization errors [49]. From the point of view of decoupling, we note that the larger the value of *c* is, the more sensitive the coupling is to the O($M^{-1}$) counterterms located at $x_0=0,T$. This is so because for larger values of *t* the footprint of the flow field $B_\mu (t,x)$ extends closer to the boundaries.

In order to attenuate the sensitivity to the O($M^{-1}$) terms, in Eq. (51) we consider a space-time volume with $T=2L$, and place the energy density $E_{\mathrm{mag}}(t,x)$ at $x_0=T/2$, in order to maximize the distance from the boundaries (cf. Sect. 4.2.3). Taking only the magnetic part of the flow energy density also helps in reducing the O($M^{-1}$) contaminations.^35^ Lastly, we note that the expectation value $\langle \cdots \rangle _\mathrm{SF,Q=0}$ in Eq. (51) is meant to be considered in the presence of the SF boundary conditions, Eqs. (47)–(48), and restricted to gauge fields in the trivial topological sector [50, 124]. The latter constraint is imposed in order to circumvent issues related to topology freezing at small lattice spacings [125, 126].

Having introduced the massive scheme of choice, we now move to the definition of the decoupling scale $\mu ^{(3)}_{\mathrm{dec}}$. We define this in terms of a *massless* finite-volume coupling. Its definition slightly differs from that of Eq. (51). Specifically, we take [49, 50],

53 $$\begin{aligned}{}[{\bar{g}}^{(3)}_{\mathrm{GF}}(\mu )]^2\equiv {t^2\over {\mathcal {N}}'} \langle E_{\mathrm{mag}}(t,x)\rangle _{\mathrm{SF},Q=0}\big |_{T=L,M=0}^{x_0=L/2,\mu =L^{-1},\sqrt{8t}=cL}\,, \end{aligned}$$

where as before we set $c=0.3$. The main differences with respect to the definition in Eq. (51) are that the coupling is evaluated at vanishing (renormalized) quark masses and the temporal extent is shorter, i.e. $T=L$. The reason for considering this specific definition is because its non-perturbative running in the ${N_{\mathrm{f}}}=3$ theory is known very precisely in the range of scales $\mu \approx 0.2-4\,\mathrm{GeV}$ (see Ref. [50] and Sect. 4.1). This gives us the freedom to choose for $\mu ^{(3)}_{\mathrm{dec}}$ the most convenient value in this range. Its physical units can in fact be inferred from combining the knowledge of the non-perturbative $\beta$-function of ${\bar{g}}^{(3)}_{\mathrm{GF}}(\mu )$ [50] and the physical scales $\mu _{\mathrm{phys}}^{(3)}$ determined in large-volume hadronic simulations [13, 91].^36^

In this study, the decoupling scale is specified by the condition

54 $$\begin{aligned}{}[{\bar{g}}^{(3)}_{\mathrm{GF}}(\mu ^{(3)}_{\mathrm{dec}})]^2= 3.95\equiv u_0\,, \end{aligned}$$

which using the information mentioned above is found to correspond to

55 $$\begin{aligned} \mu _{\mathrm{dec}}^{(3)}=789(15)\,\mathrm{MeV}\equiv L_{\mathrm{dec}}^{-1}. \end{aligned}$$

This scale is convenient in practice as, given our choice of lattice resolutions (see below), it allows us to simulate at values of the lattice spacing sensibly smaller than those typically accessible to large-volume simulations. As we shall see, this enables us to simulate quark masses up to $M\approx 4M_c$, while having *aM* effects under good control. Additionally, we can profit from some perturbative information in the treatment of O(*aM*) effects, which is expected to be accurate enough at the values of the bare coupling corresponding to the relevant lattice spacings. Lastly, the value of $\mu _{\mathrm{dec}}^{(3)}$ is low enough in energy that only a very limited part of the running in ${N_{\mathrm{f}}}=3$ QCD is needed in order to connect it to the scales $\mu ^{(3)}_{\mathrm{phys}}$ and set its physical units.

#### Determinations of the massive coupling

The next step in the strategy is to determine the value of the coupling ${\bar{g}}^{(3)}_{\mathrm{GFT}}(\mu ^{(3)}_{\mathrm{dec}},M)$ for some large quark masses. To this end, we must evaluate the coupling ${\bar{g}}^{(3)}_{\mathrm{GFT}}(\mu ^{(3)}_{\mathrm{dec}},M)$ for several values of the lattice spacing at fixed $\mu ^{(3)}_{\mathrm{dec}}$ and given *M*, and extrapolate it to the continuum limit.

The condition in Eq. (54) defines the line of constant physics along which $\mu _{\mathrm{dec}}^{(3)}$ is constant. Given a set of lattice sizes *L*/*a*, by tuning the bare coupling $g_0$ so that the massless GF-coupling has the prescribed value $u_0$, we can identify the values of *a* for which $L=L_{\mathrm{dec}}$ is fixed in physical units as $a/L\rightarrow 0$. In this respect, note that in order to compute the massive coupling ${\bar{g}}^{(3)}_{\mathrm{GFT}}(\mu ^{(3)}_\mathrm{dec},M)$ and the massless coupling ${\bar{g}}^{(3)}_\mathrm{GF}(\mu ^{(3)}_{\mathrm{dec}})$ at matching values of the lattice spacing up to O($(aM)^2$) corrections, the two must be evaluated at the same value of the O(*a*)-improved bare coupling ${\tilde{g}}_0$ [101]. The latter, we recall, is defined as [101],

56 $$\begin{aligned} {\tilde{g}}_0^2\equiv g_0^2(1+b_\mathrm{g}({\tilde{g}}_0)am_{\mathrm{q}})\,, \quad m_{\mathrm{q}}=m_0-m_\mathrm{cr}({\tilde{g}}_0)\,, \end{aligned}$$

where $m_0$ is the bare quark mass, $m_{\mathrm{cr}}$ is its critical value at which the (renormalized) quark masses vanish, and $b_\mathrm{g}({\tilde{g}}_0)$ is a function of the bare coupling to be determined. In the massless theory $m_{\mathrm{q}}=0$, and the improved bare coupling coincides with $g_0$. The values of $g_0$ determined from the condition (54) in terms of the massless coupling therefore specify the values of ${\tilde{g}}_0$ at which the massive couplings should be evaluated. According to the Symanzik improvement programme, the coefficient $b_{\mathrm{g}}({\tilde{g}}_0)$ can be tuned in order to remove O($am_{\mathrm{q}}$) effects in the matching between the massless and massive renormalization schemes. At present, however, this is only known to 1-loop order in lattice perturbation theory, where $b_{\mathrm{g}}(g_0)=0.012\,g_0^2\times {N_{\mathrm{f}}}+\mathrm{O}(g_0^4)$ [41, 101].

**Fig. 13:**
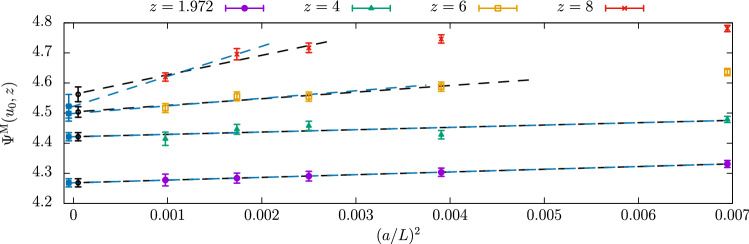
Continuum limit extrapolations of the massive couplings $\varPsi ^M(u_0,z)$ for $z=1.972,4.0,6.0,8.0$ (cf. Eq (59)) [14]. Two cuts $(aM)^2 < 1/8,1/4$ are applied in order to estimate the systematic uncertainties in the extrapolations

Together with the bare coupling, the bare quark masses must be set in order to guarantee a given value for the RGI mass *M* in the continuum limit. This is achieved by considering a value of $z=ML_{\mathrm{dec}}$, and by solving for a given lattice size $L_\mathrm{dec}/a$ the following equation for the bare quark masses,

57 $$\begin{aligned} z={L_{\mathrm{dec}}\over a} {M\over {\overline{m}}^{(3)}_\mathrm{SF}(\mu ^{(3)}_{\mathrm{dec}})} Z_{\mathrm{m}}({\tilde{g}}_0,a/L_\mathrm{dec})a{\tilde{m}}_{\mathrm{q}}\,. \end{aligned}$$

In this equation,

58 $$\begin{aligned} {\tilde{m}}_{\mathrm{q}}=m_{\mathrm{q}} (1+b_{\mathrm{m}}({\tilde{g}}_0)am_{\mathrm{q}})\,, \quad m_{\mathrm{q}}=m_0-m_{\mathrm{cr}}({\tilde{g}}_0)\,, \end{aligned}$$

is the O(*a*)-improved definition for the bare quark mass, which replaces the regular bare mass $m_{\mathrm{q}}$ in order to eliminate O($am_{\mathrm{q}}$) effects in massive schemes [101]. To this end, the function $b_{\mathrm{m}}({\tilde{g}}_0)$ must be properly chosen. The function $Z_m({\tilde{g}}_0,a/L)$, instead, refers to the renormalization constant that relates the bare quark mass to the renormalized quark mass ${\overline{m}}_{\mathrm{SF}}(\mu )$ in the SF-scheme of Refs. [127–130]. This, together with the improvement coefficient $b_{\mathrm{m}}$, and the critical mass $m_{\mathrm{cr}}$, are known non-perturbatively for the relevant parameters (see Ref. [14]). The matching factor $M/{\overline{m}}^{(3)}_{\mathrm{SF}}(\mu ^{(3)}_{\mathrm{dec}})$ then allows us to convert the renormalized quark mass in the SF-scheme at the scale $\mu _{\mathrm{dec}}^{(3)}$ to the RGI mass *M*. It can be obtained from the results of Ref. [130]. Once $am_{\mathrm{q}}$ for the given *z* is known from Eq. (57) at the values of ${\tilde{g}}_0$ given by the condition Eq. (54), using the 1-loop results for $b_{\mathrm{g}}$ we can infer from Eq. (56) the values of $g_0$ at which the massive couplings must be computed in simulations [14].

Having set the bare parameters we can finally evaluate the functions,

59 $$\begin{aligned} \varPsi ^M(u_0,z)=\lim _{a/L_{\mathrm{dec}}\rightarrow 0} \big [{\bar{g}}^{(3)}_{\mathrm{GFT}}(\mu ^{(3)}_\mathrm{dec},M)\big ]^2\big |_{[{\bar{g}}^{(3)}_{\mathrm{GF}}(\mu ^{(3)}_\mathrm{dec})]^2=u_0}\,.\nonumber \\ \end{aligned}$$

In Fig. 13 the results for the extrapolations in Eq. (59) are shown. Several values of *z* have been considered, ranging from $z\approx 2-8$, which correspond to RGI masses $M\approx 1.6-6.3\,\mathrm{GeV}$. The different values of *z* will allow us to assess the size of the non-perturbative corrections to decoupling in Eq. (43). The range of lattice sizes considered is $L/a=12-32$.

As one can see from the figure, as expected, the continuum limit extrapolations become more challenging as *z* becomes larger. However, at the smaller values of *a*/*L*, the data seem to be well described by O($a^2$) discretization errors. In order to assess systematic effects in the extrapolations, fits with different cuts in *aM* have been considered, specifically $(aM)^2<1/8,1/4$. The results from the different fits are compatible, with the results for $(aM)^2 < 1/8$ having significantly larger errors at large values of *z*, where fewer points are left after the cut is imposed. We take as final results those with cut $(aM)^2<1/8$.

**Fig. 14:**
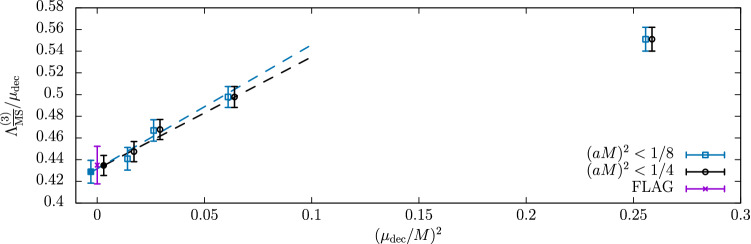
Values for $\rho =\varLambda ^{(3)}_{\overline{\mathrm{MS}}}/ \mu _{\mathrm{dec}}^{(3)}$ determined from the decoupling relation, Eq. (43) [14]. As $z=M/\mu ^{(3)}_{\mathrm{dec}}$ gets larger, the results for $\rho$ approach the FLAG value $\varLambda ^{(3)}_{\overline{\mathrm{MS}}}=343(12)\,\mathrm{MeV}$ [3] in units of $\mu ^{(3)}_\mathrm{dec}=789(15)\,\mathrm{MeV}$ (cf. Eq. (55)). The filled symbols illustrate possible extrapolations for $z\rightarrow \infty$. The results of the extrapolations show significantly smaller statistical uncertainties than the FLAG result. (Note that $\mu _{\mathrm{dec}}^{(3)}$ gives a negligible contribution to the uncertainty of $\varLambda _{\overline{\mathrm{MS}}}^{(3)}/ \mu _{\mathrm{dec}}^{(3)}$ from FLAG.) The uncertainties on $\rho$ may be further reduced with modest computational effort by improving the determination of $\varLambda ^{(0)}_{\overline{\mathrm{MS}}}/\mu _{\mathrm{dec}}^{(0)}$ from Ref. [33]. The data both at finite *z* and the extrapolations for $z\rightarrow \infty$ have been slightly shifted horizontally for better clarity

#### Results for $\varLambda _{\overline{\mathrm{MS}}}^{(3)}$

A last step separates us from applying Eq. (43) to compute $\varLambda _{\overline{\mathrm{MS}}}^{(3)}$. In order to use Eq. (43) directly in the GFT-scheme of Eq. (51) the non-perturbative running of the corresponding coupling in the pure-gauge theory, ${\bar{g}}^{(0)}_\mathrm{GFT}(\mu )$, should be known. This, however, has never been computed. On the other hand, the running of the pure-gauge coupling in the GF-scheme, ${\bar{g}}^{(0)}_{\mathrm{GF}}(\mu )$, is known very precisely [33]. In other to resolve the issue, all we have to do is to match non-perturbatively the GFT- and GF-schemes in the pure Yang-Mills theory. More precisely, we need to determine the values of the coupling $g_M={\bar{g}}^{(0)}_\mathrm{GF}(\mu ^{(0)}_{\mathrm{dec}})$ corresponding to ${\bar{g}}^{(0)}_\mathrm{GFT}(\mu ^{(0)}_{\mathrm{dec}})= \sqrt{\varPsi ^M(u_0,z)}$ for the relevant values of *z*. Given this relation we can compute,

60 $$\begin{aligned} {\varLambda ^{(0)}_{\mathrm{GF}}\over \mu _{\mathrm{dec}}^{(0)}} =\varphi _\mathrm{g,\mathrm GF}^{(0)}(g_M) =\varphi _{\mathrm{g,\mathrm GFT}}^{(0)}(\sqrt{\varPsi ^M})\,, \quad g_M=\sqrt{\chi (\varPsi ^{M})}\,,\nonumber \\ \end{aligned}$$

where $\chi ([{\bar{g}}^{(0)}_{\mathrm{GFT}}(\mu )]^2)= [{\bar{g}}^{(0)}_\mathrm{GF}(\mu )]^2$. The function $\chi$ can easily be obtained in the relevant range of couplings $[{\bar{g}}^{(0)}_\mathrm{GFT}]^2=\varPsi ^M(u_0,z)$, by computing $[{\bar{g}}^{(0)}_{\mathrm{GF}}]^2$ and $[{\bar{g}}^{(0)}_{\mathrm{GFT}}]^2$ at several matching values of *L*/*a* and $g_0$ in this range, and extrapolating their relation to the continuum limit [14].

The results for $\rho =\varLambda _{\overline{\mathrm{MS}}}^{(3)}/\mu ^{(3)}_{\mathrm{dec}}$ as obtained from Eq. (43) for different values of *z* are shown in Fig. 14. For the estimates the function $P^{\mathrm{PT}}_{0,3}(M/\varLambda _{\overline{\mathrm{MS}}}^{(3)})$ is evaluated at 5-loop order. In this respect we note that, as expected from the discussion in Sect. 3.2.2, the perturbative uncertainties in $\rho$ estimated from the effect of the last known terms of $P^{\mathrm{PT}}_{0,3}$ are completely negligible compared to the other sources of uncertainties (cf. Table 2 of Ref. [14]). As one can see from the figure, excluding the point at $z\approx 2$, the non-perturbative corrections to decoupling are small. At larger *z* values they are compatible with O($z^{-2}$) scaling, indicating that the O($z^{-1}$) corrections due to the SF boundary conditions are subdominant. For values of $M\approx 6.3\,\mathrm{GeV}$ (i.e. $z=8$) the estimated $\rho$ agrees well with the fully ${N_{\mathrm{f}}}=3$ flavor theory results for $\varLambda _{\overline{\mathrm{MS}}}^{(3)}/ \mu ^{(3)}_{\mathrm{dec}}$, where $\varLambda _{\overline{\mathrm{MS}}}^{(3)}$ is given by the FLAG average value [3] and $\mu _{\mathrm{dec}}^{(3)}$ is taken from Eq. (55). If one attempts a $z\rightarrow \infty$ extrapolation of the data the agreement becomes even better.

### Summary and miscellaneous remarks

The results of Ref. [14] put on solid grounds the application of the decoupling relation Eq. (43) as a novel strategy to determine the QCD coupling from lattice QCD. The remarkable feature of this approach is that the non-perturbative running of the coupling from the low-energy scale $\mu _{\mathrm{dec}}$ up to high energy is done entirely in the pure-gauge theory. This opens up the possibility to significantly reduce the current error on $\alpha _{\mathrm{s}}$ (cf. Sects. 5 and 4.1).

In order to translate the results for $\varLambda _{\overline{\mathrm{MS}}}^{(0)}/\mu ^{(0)}_{\mathrm{dec}}$ into $\varLambda _{\overline{\mathrm{MS}}}^{({N_{\mathrm{f}}})}/\mu ^{({N_{\mathrm{f}}})}_{\mathrm{dec}}$, the strategy relies on two crucial ingredients. The first ingredient is the computation of a massive coupling ${\bar{g}}^{({N_{\mathrm{f}}})}_{{{\mathcal {O}}}}(\mu ,M)$ at the low-energy scale $\mu ^{({N_{\mathrm{f}}})}_{\mathrm{dec}}$ in an unphysical set-up with ${N_{\mathrm{f}}}$-flavors of degenerate massive quarks of mass $M\gg \mu ^{({N_{\mathrm{f}}})}_{\mathrm{dec}}$. Exploiting the decoupling of the massive quarks, the scales $\mu ^{({N_{\mathrm{f}}})}_{\mathrm{dec}}$ and $\mu ^{(0)}_{\mathrm{dec}}$ can be connected through the massive coupling and so the fundamental ${N_{\mathrm{f}}}$-flavor and effective pure-gauge theories. The second ingredient is the use of high-order perturbation theory for estimating the ratio of the $\varLambda$-parameters of the two theories, $\varLambda _{\overline{\mathrm{MS}}}^{(0)}/\varLambda _{\overline{\mathrm{MS}}}^{({N_{\mathrm{f}}})}$, given by the function $P_{\mathrm{0,f}}(M/\varLambda _{\overline{\mathrm{MS}}}^{({N_{\mathrm{f}}})})$. Control on the determination of the massive coupling can be achieved by employing a suitable finite-volume scheme. Thanks to the fact that the physical volume does not need to be large, small lattice spacings can be simulated, and safe continuum limit extrapolations of ${\bar{g}}^{({N_{\mathrm{f}}})}_{{{\mathcal {O}}}}(\mu _{\mathrm{dec}}^{({N_{\mathrm{f}}})},M)$ with $\mu ^{({N_{\mathrm{f}}})}_{\mathrm{dec}}=\mathrm{O}(1\,\mathrm{GeV})$ can be taken for quark masses up to a few GeV. At these large masses, perturbation theory for the function $P_{\mathrm{0,f}}(M/\varLambda _{\overline{\mathrm{MS}}}^{({N_{\mathrm{f}}})})$ works extremely well and non-perturbative O($M^{-2}$) corrections to the decoupling relations are found to be small.

From the results of figure 14 we can appreciate how the strategy based on decoupling promises great accuracy. The $z\rightarrow \infty$ extrapolations give in fact results for $\rho$ which are about a factor 2 more precise than those obtained using the current FLAG estimate for $\varLambda _{\overline{\mathrm{MS}}}^{(3)}$ and $\mu _{\mathrm{dec}}^{(3)}$ from Eq. (55). In order to set this result on firmer grounds, however, a robust $z\rightarrow \infty$ extrapolation must be performed. To this end, it is important that the continuum limit extrapolations for the massive couplings $\varPsi ^M(u_0,z)$ are made more solid at the largest (most relevant) *z* values by investing some additional computational effort. The lattices used for the computations entering Fig. 13 are in fact rather modest. The largest simulated lattices have $L/a=32$, $T/a=64$. In addition, the large quark masses make these simulations significantly cheaper than the more common massless SF simulations. A factor two finer lattices are hence within reach with affordable computational resources. These lattices will allow us to consider larger quark masses too, and thus improve even further the control on the $z\rightarrow \infty$ extrapolations. All in all, we can expect that after these improvements the final determination for $z\rightarrow \infty$ will be at least as precise as the results in Fig. 14 promise, but will include conservative estimates for all systematics.

It is important to note at this point that a significant fraction of the error on $\rho$ at finite *z* comes from the uncertainty on $\varLambda _{\overline{\mathrm{MS}}}^{(0)}/ \mu _{\mathrm{dec}}^{(0)}$ from Ref. [33], which is about $1.5\%$ (cf. Fig. 14). A reduction of this error down to $0.5\%$ or so is desirable and in principle possible. Given the importance of the result for the determination of $\varLambda _{\overline{\mathrm{MS}}}^{(3)}$, however, it is crucial that this error reduction is achieved robustly. As discussed in Sect. 2.3.2, even though the determination of $\varLambda$ in the pure Yang–Mills theory is very much simplified from the computational point of view compared to QCD, the problem is yet non-trivial and care must be taken, especially if such a high precision is desired. For this reason, it is mandatory that the results for $\varLambda _{\overline{\mathrm{MS}}}^{(0)}$ are corroborated by investigating different strategies where the estimates of systematic uncertainties are put to a stringent test. As we have seen, there is currently tension among different determinations of $\varLambda _{\overline{\mathrm{MS}}}^{(0)}$, some of which quote the desired sub-percent precision (cf. Sect. 2.3.2 and Ref. [33]). Studies as the ones of Refs. [34, 35] are hence encouraged in order to set the actual accuracy at which we currently know $\varLambda _{\overline{\mathrm{MS}}}^{(0)}$.

This corroboration goes hand in hand with the exploration of new strategies for the determination of $\varLambda ^{(0)}_{\overline{\mathrm{MS}}}$. In this respect we point out the results of Ref. [35], where an alternative way to do step-scaling for GF-based couplings was proposed and tested. In short, the change of renormalization scale in the coupling is first achieved by changing the flow time at fixed physical volume and in a second step the physical volume is changed at fixed flow time. This has to be compared with the traditional situation where both flow time and spatial size are changed at once. One of the interesting features of the approach is that it amounts to a reanalysis of data gathered from a traditional step-scaling study. However, the systematics to deal with are quite independent given the different continuum limit extrapolations involved. By comparing the two analysis one can stringently test the assumptions made in one or the other approach.

Furthermore, it would be interesting to employ other definitions of finite-volume schemes based on either different observables and/or set-ups. For instance, the GF-coupling with twisted boundary conditions explored in Refs. [106, 131–133] is promising. Differently from the SF case it enjoys full translational invariance and yet its perturbative expansion in finite volume appears feasible [134]. Of course, standard periodic boundary conditions are also an option [48] despite the difficulties with perturbation theory in this set-up. In fact, in cases where the perturbative information is limited or the relevant perturbative expansion is poorly convergent, a viable option is to non-perturbatively match the given finite-volume scheme to some other scheme for which the perturbative $\beta$-function is known to high-loop order and it is well behaved (see e.g. Refs. [33, 50]). This may allow for a significantly more precise determination of $\varLambda _{\overline{\mathrm{MS}}}^{(0)}$ (cf. Sect. 2.3.2). In this respect, we note that a powerful framework for automated numerical high-loop calculations in finite volume has been recently developed and successfully applied [51, 135].

Another idea that may be interesting to consider is the determination of $\varLambda ^{(0)}_{\overline{\mathrm{MS}}}$ based on the infinite-volume $\beta$-function of GF-based couplings, following the strategy of Refs. [136–138] (see also Ref. [103]). In this approach, the infinite-volume results are obtained by extrapolations from small-volume simulations, which might already be at hand from a conventional step-scaling study. If the (non-trivial) infinite-volume extrapolations can be performed in a controlled way and the convergence to the perturbative regime of the chosen scheme is fast enough, this strategy may allow for interesting crosschecks of the results from step-scaling. In this case, the framework developed in Ref. [139] can be used to obtain the necessary infinite-volume perturbative information to high-loop order.

Besides applying different strategies for the calculation of $\varLambda _{\overline{\mathrm{MS}}}^{(0)}$, different techniques can be considered for the QCD part of the decoupling strategy as well. A simple extension is to consider different schemes for the massive finite-volume coupling. In the case of ${N_{\mathrm{f}}}=3$ QCD, periodic and twisted boundary conditions would avoid entirely the issue with O($M^{-1}$) contaminations. For ${N_{\mathrm{f}}}=4$ QCD twisted boundary conditions cannot be implemented [140], but twisted-mass fermions with SF boundary conditions or regular massive quarks with chirally rotated boundary conditions are available options (cf. Sect. 4.2.3).

A substantially different approach is to avoid entirely finite-volume couplings and rely on heavy-quark decoupling in hadronic quantities. Particularly interesting observables to consider are the popular gluonic scales ${\mathcal {S}}=t_0^{-1/2}, t_c^{-1/2},w_0^{-1}, r_0$ [47, 52, 71, 83]. In this case, the decoupling relation is applied more directly in the form of Eq. (27), specifically,

61 $$\begin{aligned} {\varLambda ^{({N_{\mathrm{f}}})}_{\overline{\mathrm{MS}}}\over {\mathcal {S}}^{({N_{\mathrm{f}}})}(M)} P^{\mathrm{PT}}_{0,\mathrm f}\bigg ({M\over {\varLambda ^{({N_{\mathrm{f}}})}_{\overline{\mathrm{MS}}}}} \bigg ) = {\varLambda ^{(0)}_{\overline{\mathrm{MS}}}\over {\mathcal {S}}^{(0)}}\, +\mathrm{O}(g_*^{2n-2},M^{-2}). \end{aligned}$$

Once the right hand side is known from computing the running in pure-gauge theory, the perturbative approximation $P^{\mathrm{PT}}_{0,\mathrm f}({M/\varLambda ^{({N_{\mathrm{f}}})}_{\overline{\mathrm{MS}}}})$ to the ratio of $\varLambda$-parameters can be used to solve the above equation for $\varLambda _{\overline{\mathrm{MS}}}^{({N_{\mathrm{f}}})}/{\mathcal {S}}^{({N_{\mathrm{f}}})}(M)$. All that is left to do to determine $\varLambda _{\overline{\mathrm{MS}}}^{({N_{\mathrm{f}}})}$ is then to fix the physical units of the low-energy quantity ${\mathcal {S}}^{({N_{\mathrm{f}}})}(M)$ computed in a theory with ${N_{\mathrm{f}}}$ heavy quarks of RGI mass *M*. This can be obtained by relating ${\mathcal {S}}^{({N_{\mathrm{f}}})}(M)$ to some convenient physical scale $\mu _\mathrm{phys}^{({N_{\mathrm{f}}})}$ evaluated at physical quark masses via the bare parameters. As discussed in Sect. 4.2.2, it might be difficult to reach large masses *M* with this approach, while having discretization errors and finite-volume effects under control. Some compromises are likely necessary in order to reach high enough *M* values to be able to control decoupling corrections. On the other hand, the studies of Refs. [71, 84] show that interesting results may be obtained if masses close to that of the charm can be reliably reached. This makes the strategy worth being explored.

## Conclusions

In this contribution we presented a novel strategy for the determination of the QCD coupling using lattice QCD. It exploits the decoupling of heavy quarks at low energy to connect the pure Yang-Mills theory and QCD with ${N_{\mathrm{f}}}$ flavors of quarks. The main result is that the computation of the running of the coupling from a known low-energy scale $\mu _{\mathrm{dec}}=\mathrm{O}(1\,\mathrm{GeV})$ up to high energies can be done entirely in the pure-gauge theory instead of ${N_{\mathrm{f}}}$-flavor QCD. Considering ${N_{\mathrm{f}}}=3$ or 4, this paves the way for unprecedented precision determinations of $\varLambda _{\overline{\mathrm{MS}}}^{({N_{\mathrm{f}}})}$ from which $\varLambda _{\overline{\mathrm{MS}}}^{(5)}$ and $\alpha _{\mathrm{s}}$ can be obtained. In Ref. [14] the potential of these methods was successfully established in the determination of $\varLambda _{\overline{\mathrm{MS}}}^{(3)}$. We now want to put this result into context of a future precision $\alpha _{\mathrm{s}}$ extraction.

As presented in Sect. 4.3, the results for $\varLambda _{\overline{\mathrm{MS}}}^{(3)}/ \mu ^{(3)}_{\mathrm{dec}}$ from decoupling have an uncertainty which is about half the one obtained using the FLAG average $\varLambda _{\overline{\mathrm{MS}}}^{(3)}=343(12)\,\mathrm{MeV}$ [3] and $\mu _\mathrm{dec}^{(3)}=789(15)\,\mathrm{MeV}$ from Eq. (55) (cf. Fig. 14). As discussed in Sect. 4.4, by investing some modest computational effort, this result can be set on very solid grounds by improving the continuum limits of the massive couplings (cf. Fig. 13) and performing a robust $z\rightarrow \infty$ extrapolation (cf. Fig. 14). Considering lattices twice as large as the ones simulated in Ref. [14] is in fact affordable. With such lattices we can expect that the continuum limit extrapolations of $\varPsi ^M(u_0,z)$ in Eq. (59) can be performed with confidence also at the largest masses investigated so far ($M\approx 6\,\mathrm{GeV}$). In addition, we will be able to consider some larger *z* values, too.

The precision on $\varLambda _{\overline{\mathrm{MS}}}^{(3)}/\mu ^{(3)}_\mathrm{dec}$ can be further improved significantly by reducing the uncertainties coming from the pure-gauge determination of $\varLambda _{\overline{\mathrm{MS}}}^{(0)}/\mu ^{(0)}_{\mathrm{dec}}$, which has the (conservative) error of 1.5% [33]. As noticed in Sect. 4.4, this error can in principle be reduced by a substantial factor, e.g. down to 0.5%. However, while it is certainly possible to reach such a high precision in a given computation, it is crucial that the results of different analysis and groups corroborate it. At present, there is in fact tension among determinations of $\varLambda _{\overline{\mathrm{MS}}}^{(0)}$ involving results with sub-percent accuracy (cf. Sect. 2.3.2). It is important to understand the origin of these differences. We hope that the renovated interest in this quantity brought by this new strategy motivates the community to resolve the issue and contribute to a high-precision determination.

Once the above steps are achieved the precision on $\varLambda _{\overline{\mathrm{MS}}}^{(3)}$ will be limited by the present error on $\mu ^{(3)}_{\mathrm{dec}}$, which is about 2%. A reduction of this error down to $1\%$ is however foreseeable. It requires, first of all, to improve the results for the running of the GF-coupling ${\bar{g}}^{(3)}_{\mathrm{GF}}(\mu )$ at energies $\mu <\mu _\mathrm{dec}$ [50]. We recall that this is needed in order to connect $\mu ^{(3)}_{\mathrm{dec}}$ with the hadronic scales $\mu ^{(3)}_{\mathrm{phys}}$ used to set the physical units of the theory (cf. Sect. 4.3). Work in this direction has already started as part of the HQET efforts by the ALPHA Collaboration (cf. Ref. [141]). Secondly, the scale setting in terms of the physical scales $\mu _\mathrm{phys}^{(3)}$ must be improved as well. In practice, this means to obtain a more precise determination for a convenient low-energy reference scale in ${N_{\mathrm{f}}}=3$ QCD in physical units, like for example, $\mu _{\mathrm{ref}}^*=1/\sqrt{8t_0^*}$ (cf. Ref. [91]). A precision of $1\%$ or better on this or similar scales is desirable. This is expected to be possible by exploiting the new CLS ensembles close to the physical point [20, 92, 142]. Also in this case, however, corroboration from different strategies and groups is important.

Through all these steps a determination of $\varLambda _{\overline{\mathrm{MS}}}^{(3)}$ with a final uncertainty of 1-2% appears feasible. As discussed in Sect. 3.3.2, at this level of precision $\varLambda _{\overline{\mathrm{MS}}}^{(5)}$ can yet be obtained from $\varLambda _{\overline{\mathrm{MS}}}^{(3)}$ by relying on the perturbative decoupling of the charm quark, eventually including some conservative estimate for the unaccounted non-perturbative corrections. A determination of $\alpha _{\mathrm{s}}(M_Z)$ at the level of 0.4% is therefore within reach thanks to the novel techniques. To further halve the error on $\alpha _{\mathrm{s}}(M_Z)$, on the other hand, requires several issues to be reconsidered. Non-perturbative decoupling effects might become relevant, and one might need to include electromagnetic and $m_u\ne m_d$ effects in the lattice computations in order to set the physical scale of the theory to a greater level of accuracy (cf. Sect. 3.3.2 and see also discussion in Ref. [15]).

Before concluding we want to note that even though our emphasis was on the determination of $\varLambda _{\overline{\mathrm{MS}}}^{({N_{\mathrm{f}}})}$, the ideas presented can be extended to solve other RG problems. A clear case is that of the quark masses, where one can replace their running in ${N_{\mathrm{f}}}$-flavor QCD with the one in the quenched approximation. In Ref. [86] a similar application was in fact explored in order to study the non-perturbative charm-quark effects in the determination of the charm-quark mass itself. More complicated composite operators, like for instance four-quark operators, require more thought. First of all, a study of the quality of their perturbative decoupling relations is necessary in order to establish whether the strategy has any chance to be applied in the first place. Then, an investigation of the non-perturbative decoupling corrections must follow.

In conclusion, we can affirm that the decoupling of heavy quarks enters at full right in the renormalization toolkit of the lattice field theorist. Many more applications of these powerful ideas in lattice QCD and lattice field theory in general are likely to come.

## Data Availability

This manuscript has no associated data or the data will not be deposited. [Authors’ comment: The contribution is a review of previously published material. No new data has been generated for its preparation.]

## Footnotes

1 We here adopt the common notation $\alpha _{\mathrm{s}}(\mu )\equiv \alpha _{\overline{\mathrm{MS}}}^{(5)}(\mu )$, where $\alpha _{\overline{\mathrm{MS}}}^{(5)}(\mu )$ is the QCD coupling of the 5-flavor theory renormalized in the $\overline{\mathrm{MS}}$-scheme (see e.g. Ref. [5]). Note that for the ease of notation we often omit to write explicitly the $\mu$-dependence of the coupling.

2 Note that in general the coupling $\alpha _{\mathrm{s}}$ to be considered here should be the QCD coupling of the relevant ${N_{\mathrm{f}}}$-flavor theory, i.e. $\alpha _{\mathrm{s}}(\mu )\equiv \alpha _{\overline{\mathrm{MS}}}^{({N_{\mathrm{f}}})}(\mu )$, from which $\alpha _{\overline{\mathrm{MS}}}^{(5)}(M_Z)$ can eventually be extracted (cf. e.g. Ref. [3] and Sect. 3.1.2). At this stage, however, we prefer to keep the discussion simple. We shall return to this point later in detail.

3 Our knowledge of the form of non-perturbative effects is in fact rather limited. In addition, strictly speaking, perturbative and non-perturbative contributions cannot really be separated due to the asymptotic nature of the series (see e.g. Ref. [17]). However, as the discussion is at this point qualitative we simply adopt the simplistic representation of non-perturbative effects as power corrections to the perturbative expansion.

4 Experimental observables are in principle sensitive also to any New Physics. How this affects the extracted value of $\alpha _{\mathrm{s}}$, however, is something hard to assess.

5 Here and in the following we assume that ${{\mathcal {O}}}(q)$ is a properly renormalized lattice quantity with a well-defined continuum limit and free from infrared divergences. Alternative strategies extract $\alpha _{\mathrm{s}}$ from bare lattice quantities by taking $q\propto a^{-1}$ and expressing their expansion in lattice perturbation theory in terms of $\alpha _{\mathrm{s}}$ (see e.g. Ref. [18]). In these cases, discretization errors and scale dependence of ${{\mathcal {O}}}(q)$ are entangled. Addressing systematic uncertainties becomes more subtle and requires a separate discussion (see Ref. [3]).

6 We shall return on these points in more detail in Sect. 3.

7 Interestingly, Eq. (8) provides an indirect non-perturbative definition of $\varLambda _{\overline{\mathrm{MS}}}$ through the $\varLambda$-parameter of any non-perturbative scheme.

8 The issue of fixing the scale of a theory with an unphysical value of ${N_{\mathrm{f}}}$ will be addressed in Sect. 3.

9 This is the case because for all these schemes the *N*-loop $\beta$-function, $\beta ^{\mathrm{PT}}_{{\mathcal {O}}}$, has been inferred from $\beta ^\mathrm{PT}_{\overline{\mathrm{MS}}}$ at *N*-loops using the matching relation Eq. (9) and $\beta ^{\mathrm{PT}}_{{\mathcal {O}}}$ at $(N-1)$-loop order.

10 We recall that a series is said to be asymptotic to the function $f(\lambda )$, $\lambda \in {\mathbb {R}}$, if: $|f(\lambda )-\sum _{j=0}^N a_j\lambda ^j\big | \overset{\lambda \rightarrow 0}{\rightarrow }\mathrm{O}(\lambda ^{N+1})$, $\forall N$. Note in particular that at fixed $\lambda$, larger *N* does not necessarily imply a better approximation of the series to the function.

11 Traditionally only the $\nu =0$ scheme has been considered in applications, see e.g. Refs. [40–45] for some important examples.

12 For comparison the 3-loop coefficient of $\beta _{\overline{\mathrm{MS}}}$ for ${N_{\mathrm{f}}}=3$ is $(4\pi )^3 b_2^{\overline{\mathrm{MS}}}= 0.324$.

13 The scale factor $s^*$ is defined by (cf. Eq. (8)):

$$\begin{aligned} s^*={\varLambda _{\overline{\mathrm{MS}}}\over \varLambda _{{{\mathcal {O}}}}}= \exp \bigg \{{c_1(1)\over 2b_0}\bigg \}\,. \end{aligned}$$

where $b_0$ is the 1-loop coefficient of the $\beta$-function in Eq. (7), and $c_1(1)$ is the 1-loop coefficient of the matching relation between the $\overline{\mathrm{MS}}$-coupling ${\bar{g}}^2_{\overline{\mathrm{MS}}}(\mu )$ and the coupling of interest ${\bar{g}}^2_{{\mathcal {O}}}(\mu )$ (cf. Eq. (9)). With the choice $s=s^*$, the $k=1$ term in Eq. (9) vanishes.

14 A similar earlier study on the challenges of extracting $\varLambda _{\overline{\mathrm{MS}}}$ in both ${N_{\mathrm{f}}}=2$ QCD and the pure-gauge theory using the $\mathrm{qq}$-coupling can be found in Ref. [53]. For a recent application of this scheme for the computation of $\alpha _{\mathrm{s}}$ and a detailed account of the most recent results and developments see Refs. [16, 54].

15 Perturbation theory predicts: $\sigma (u)\overset{u\rightarrow 0}{\approx } u+s_0u^2+\mathrm{O}(u^3)$, with $s_0=2b_0({N_{\mathrm{f}}})\ln (2)$, where $b_0({N_{\mathrm{f}}})$ is the universal 1-loop coefficient of the $\beta$-function, Eq. (7) (see e.g. Ref. [32]). The close agreement between the non-perturbative data for $\sigma (u)$ and 1-loop perturbation theory is quite peculiar, considering the fact that it holds up to $\sigma (u)=\mathrm{O}(10)$. We refer the interested reader to Ref. [50] for a detailed discussion about this point.

16 A relevant exception is the case of the SF, for which the leading discretization errors are parametrically of O(*a*) (cf. Sect. 4.2.3). In applications, however, the O(*a*) effects are subdominant with respect to the O($a^2$) effects, and often also compared to the statistical errors. The precision studies of Refs. [32, 33, 50] thus opt for treating the O(*a*) effects as (small) systematic uncertainties in the data, and perform continuum extrapolations assuming leading O($a^2$) effects. In this respect, we note that in Refs. [61, 62] the $\varGamma _i$ relevant for the O(*a*) effects in the (pure-gauge) SF have been computed. The results support the treatment of O(*a*) effects pursued in Refs. [32, 33, 50] (cf. the given references for the details).

17 The results refer to the contributions to discretization effects coming from the lattice action, considering several popular options (cf. Ref. [62]). If the relevant observable is not a spectral quantity, additional effects originating from the lattice fields that define it are present. These depend on the specific observable and choice of discretization (see, e.g. Refs. [61, 62]).

18 In the case of the pure-gauge theory only two terms from the lattice action contribute to the O($a^2$) effects. The $\varGamma _i$ for different options can be found in Ref. [61]. In all cases, $\varGamma _i > rsim 0.6$.

19 It is clear that even though the SymEFT can predict the form of the leading asymptotic discretization errors, it cannot predict the region where these dominate over formally suppressed contributions. In practice, it may thus be difficult to establish the regime of applicability of the results from SymEFT.

20 A recently expanded discussion in Ref. [35] provides another clear illustration of the difficulties of continuum limit extrapolations of precise coupling data using results from the pure-gauge theory (cf. Figs. 6 and 7 of this reference and related discussion). We strongly recommend the interest reader to consult this reference. We moreover refer to the important pioneering studies of Refs. [59, 60] in the non-linear $\sigma$-model in two dimensions.

21 For ease of notation we do not use any symbol to indicate the generic scheme of renormalized couplings. We however assume that, unless otherwise stated, the couplings are defined in a mass-independent scheme.

22 This means that the theory lives in infinite space-time or in a finite volume with (some variant of) periodic boundary conditions. The special but relevant case of a finite volume with boundaries will be considered in Sect. 4.2.3.

23 Note that for the sake of argument we exclude the uninteresting case of ${N_{\ell }}=1$, for which a proof of this result is to our knowledge missing.

24 The unsystematic approximation $P^{(1)}_{\ell , \mathrm f}$ obtained in Ref. [71] happens to be quite close to the different orders of the perturbative expansion of $P_{\ell ,\mathrm f}$ for the values of ${N_{\mathrm{f}}}$ and ${N_{\ell }}$ considered and $M/\varLambda \lesssim 30$. For this reason it is used here to get the overall magnitude of the function $P_{\ell , \mathrm f}$. On the other hand, the different orders of perturbation theory are not expected to converge to $P^{(1)}_{\ell , \mathrm f}$ as $M/\varLambda \rightarrow \infty$ since this approximation does not have the correct asymptotic limit [71].

25 For simplicity we consider also here the case where the light quarks are massless.

26 Note that the number of light quarks on the r.h.s. of this equation is implicitly given by the difference between ${N_{\mathrm{f}}}$ and the number ${N_{\mathrm{h}}}$ of heavy quarks of which the mass is varied.

27 The value of the charm-quark mass in simulations is set by targeting $M_c/\varLambda _{\overline{\mathrm{MS}}}^{(2)}=4.87$, which is obtained using $\varLambda _{\overline{\mathrm{MS}}}^{(2)}=310\,\mathrm{MeV}$ from Ref. [88] and $M_c=1510\,\mathrm{MeV}$ from Ref. [89] (cf. Ref. [71] for the details).

28 The deviation $\varDelta \log (P_{0,2})$ of the full function $\log (P_{0,2})$ from its perturbative approximation is obtained by integrating $\varDelta \eta ^M_{0,2}$ in $\log (M/\varLambda )$ from the value of $M/\varLambda$ of interest up to $M/\varLambda =\infty$ (cf. Eq. (34) and Ref. [71]). In order to translate to $P_{0,2}$ the estimate for the non-perturbative corrections to $\eta ^M_{0,2}$ made for $M\lesssim M_c$, one therefore needs to make some assumptions on how these approach the limit $M/\varLambda \rightarrow \infty$. Depending on the assumptions, more or less conservative estimates are obtained.

29 We recall that with the current state-of-the-art algorithms, the cost of QCD simulations for reducing the lattice spacing *a* at fixed physical volume and number of independent configurations scales $\propto a^{-7}$ (cf. e.g. Ref. [99]). Hence, reducing the lattice spacing by a factor 2 or so requires a factor of O(100) in the cost.

30 We take the opportunity to point out the study of Ref. [100] where ideas based on heavy-quark decoupling are considered in order to address other RG-related problems in the context of Beyond the Standard Model Physics.

31 Note that the relation is non-perturbatively valid at this point as $\varLambda _{\overline{\mathrm{MS}}}$ can be expressed exactly in terms of any non-perturbative scheme (cf. Sect. 2.2.2).

32 The fact that we can set a unique scale for all theories with different *M* values is a direct consequence of the fact that they all share the very same $\varLambda$-parameter and this is *M* independent.

33 The list of O($M^{-1}$) counterterms for the more general case, including the situation where the effective theory also contains light quarks, can be inferred from the O(*a*) counterterms of the Symanzik effective theory for the SF [101]. It should be clear that the analogy only refers to the fields entering the counterterms and, in general, it does not extend to their coefficient functions $\omega _b$. In particular, one expects different bases of counterterm fields to appear at higher orders in the effective theories, as the two theories have different defining symmetries. From these observations, one concludes that also finite-volume couplings based on open-SF [103] or open [104] boundary conditions are affected by O($M^{-1}$) corrections.

34 For ease of presentation in the following we use continuum notation to introduce the relevant definitions while referring to the literature for the corresponding lattice expressions.

35 These choices made for reducing O($M^{-1}$) effects are also effective in reducing the sensitivity to the O(*a*) effects stemming from the space-time boundaries (cf. Sect. 4.2.3).

36 The latter are given by a combination of pion and kaon decay constants (cf. Refs. [13, 91] and Sect. 4.1 for more details).
